# B-cell DNA methylation signature in response to hepatitis B virus vaccination in females and males

**DOI:** 10.3389/fimmu.2026.1734384

**Published:** 2026-04-10

**Authors:** Katarzyna Malgorzata Kwiatkowska, Simona Anticoli, Stefano Salvioli, Luciano Calzari, Davide Gentilini, Claudia Sala, Gastone Castellani, Christian Albano, Nicoletta Vonesch, Paola Tomao, Rita Carsetti, Paolo Garagnani, Anna Ruggieri

**Affiliations:** 1 Department of Medical and Surgical Sciences (DIMEC), University of Bologna, Bologna, Italy; 2 Center for Gender Specific Medicine, Istituto Superiore di Sanità, Rome, Italy; 3 IRCCS Azienda Ospedaliero-Universitaria di Bologna, Bologna, Italy; 4 Bioinformatics and Statistical Genomics Unit, Istituto Auxologico Italiano IRCCS, Milan, Italy; 5 Department of Brain and Behavioral Sciences, University of Pavia, Pavia, Italy; 6 Immunology Research Area, B cell Unit, Ospedale Pediatrico Bambino Gesù IRCCS, Rome, Italy; 7 Department of Occupational and Environmental Medicine, Epidemiology and Hygiene, Italian Workers’ Compensation Authority (INAIL), Rome, Italy

**Keywords:** B lymphocytes, DNA methylation, epigenetics, health care workers, hepatitis B, vaccine

## Abstract

**Introduction:**

Sex-based differences in immune responses to vaccination are well-documented, yet the underlying epigenetic mechanisms remain poorly understood. This study investigates DNA methylation profiles in B cells following hepatitis B virus (HBV) vaccination, with a focus on sex-specific patterns.

**Methods:**

Using high-resolution genome-wide methylation analysis, we examined post-vaccination samples from healthy male and female health care workers.

**Results:**

Our results reveal distinct methylation signatures associated with vaccine response, with several loci showing sex-dependent differential methylation. Pathway analysis identified immune-related genes and regulatory elements potentially involved in B cell activation and memory formation. Our findings show that DNA methylation levels differ between responders versus non-responders to HBV vaccination and these alterations vary with biological sex.

**Discussion:**

Understanding these epigenetic variations may open new perspective on vaccination practice. Collecting data on B cell epigenetics in different vaccination protocols could improve our knowledge on immunization function and contribute to more personalized vaccination strategies.

## 1 Introduction

The hepatitis B virus (HBV) continues to pose a significant global health threat, with around 257 million individuals living with chronic HBV infection in 2022 and over 820,000 deaths globally attributed to its complications, such as liver cirrhosis and hepatocellular carcinoma, in 2019 (1). Hepatitis B accounts for approximately 50% of liver cancer-related deaths and 33% of liver cirrhosis-related deaths in the world, making it the third leading cause of cancer mortality, following lung and colorectal cancers (2). Vaccination against HBV has demonstrated high effectiveness in preventing infection and reducing disease burden (3). However, about 5-10% of the population doesn’t achieve a protective anti-HBs antibody level (≥ 10 mIU/ml) (4). There is strong evidence that several factors like age, sex, genetic background, and environmental exposure influence HBV vaccine responsiveness (5).

Epigenetic modifications are increasingly understood as crucial regulators of the immune response. In particular, changes in DNA methylation have been found to underlie the differentiation and activation of immune cells, including B cells (6).

Studies analyzing the DNA methylome of human B cells have uncovered the importance of DNA methylation for humoral response to antigens and for memory B cells development (7, 8). Lai and collaborators showed that, following antigen stimulation, DNA methylation alterations contribute to the transition of the naïve B cells to the germinal center (GC) and memory B cells (8). However, the available evidence in the literature on the role of DNA methylation changes in response to vaccination is limited, primarily focusing on influenza vaccination and on methylation patterns of PBMC samples, which are heterogenous since vary across individuals regarding types and frequencies of cell populations (9–12). The epigenetic differences in B cells between responders and non-responders to HBV vaccination were previously found in methylation-based surrogates and stochastic epimutations (13, 14). The present study concentrates on general and sex-specific DNA methylation signatures in B cells post HBV vaccination. While differences in immune responses based on sex have been documented, such as higher antibody titers in females compared to males after HBV vaccination (15, 16), the epigenetic basis for these variations remains poorly understood. By examining DNA methylation patterns in B cells of vaccinated individuals, we aimed to identify sex-specific epigenetic markers linked to vaccine-induced immune responses. These findings could offer valuable insights into the mechanisms behind individual differences in vaccine efficiency and could have implications for improving vaccination strategies based on sex.

## 2 Materials and methods

This study further refines the exploration of DNA methylation and the analysis of response to the HBV vaccine in isolated B cells as described in Kwiatkowska et al. (13).

### 2.1 Participants and response to vaccination

The study cohort was provided by Bambino Gesù Children’s Hospital in Rome (Italy). All sample donors were local healthcare workers of European descent who received the mandatory HBV vaccination, according to the Italian law n. 165/1991 (17), following the vaccination schedule (vaccine brand is not specified by the legislative regulation). Offspring of HbsAg+ mother, HBV positive or unvaccinated individuals were excluded from the study, as well as individuals with immunomodulating drugs and immunological comorbidities. Enrolled participants were subjected to periodic health surveillance with assessment of the post anti-HBV vaccine protection level through anti-HBs titers. There was no specific moment in relation to the date of the primary vaccination when these periodic visits were scheduled. Two phenotypic groups were identified based on the anti-HBs titers: responders (R) had ≥10 mIU/ml and non-responders (NR) had <10 mIU/ml (18). Non-responders received a booster dose with Engerix-B^®^ (GlaxoSmithKline), and serum antibody levels were measured 30 days after immunization. If anti-HBsAg antibody concentration remained <10mIU/ml, HCWs were considered true non-responders and included in this study, in agreement with the European recommendations and the Italian law (19, 20). Blood samples were collected during the periodic ambulatory visit in the case of responders and between 1–5 days after the post-booster anti-HBs titer test results in the case of non-responders. Prior to the enrollment written informed consent was obtained from all the participants.

For all the included participants additional data on chronological age, biological sex and blood cell count in withdrawn samples was collected. The presence of the differences between group characteristics was verified with Pearson’s Chi-squared test (for categorical variables) or Welch’s two sample t-test (for quantitative variables).

### 2.2 Whole-genome DNA methylation assay

PBMC samples were collected from all the donors soon after the measurements of antibody levels (1–5 days after the test results). B cells were isolated by negative selection (RosetteSep™ Human B Cell Enrichment Cocktail, Stemcell Technologies) according to the manufacturer’s instructions. The B-cell purity was between 92% and 99% with contaminating cells being CD45neg. In previous studies we found that the non-responder status was not due to intrinsic B-cell defects in the HCW subjects of our institution (21). Before B-cell separation, however, we performed FACS analysis on 69 HCWs and we found that the size of the naive, memory and plasma blast populations was in the expected range (Geomean of naive 47.67 ± 1.303%, memory 35,48 ± 1.421% and PB 0,7893 ± 2.181% for adults). We performed genomic DNA extraction (QIAamp DNA Blood Mini Kit, Qiagen) and quantification (Qubit dsDNA Broad Range Assay Kit, Thermo Fisher Scientific) according to manufacturers’ instructions. Normalized DNA (1200ng in 50uL) was bisulfite-converted (EZ-96 DNA Methylation Kit Deep-Well, Zymo Research) and subjected to DNA methylation assay (Infinium Human MethylationEPIC BeadChip, Illumina) following standard protocols. All the processing steps were performed with accurate sample randomization. Multidimensional scaling (MDS) analysis was performed to verify effectiveness of sample randomization. Sample batches (**Supplementary Table S1** in **Supplementary Data 1**) related to DNA storage and extraction, methylation array, sex and phenotypic group were included.

### 2.3 DNA methylation analysis

Output *idat* files and collected data were manipulated using R (v3.6.3; *minfi* R Bioconductor package) in Linux environment. Data quality control, Noob normalization and β-values calculation were performed as described previously (13). From the analysis, we excluded probes: located on sex chromosomes, mapping to SNPs, non-specific, cross-reactive, variant-containing, masked from mapping and with multiple alignments, as recommended (22–24). Eventually, for each cohort analysis (joined/female/male) we filtered out probes with bi- or tri-modal distribution of β-values using data clustering algorithm DBSCAN (25). Methylation analysis of probes mapping to chromosomes X and Y was performed using sex-stratified approach (26).

In total, there were 866,238 probes assessed with Illumina EPIC array. Following the steps described above, 203,888 probes were excluded from the analysis and **Supplementary Table S2** in **Supplementary Data S1** delineates the pruning process. **Supplementary Data 2** provides QC report summarizing distribution of β-values, bisulfite conversion efficiency, probe quality, sample detection p-values and sample call rates. Running DBSCAN, we further filtered out the probes with bi- or tri-modal distribution to remove DNA methylation probes mapping on common genetic polymorphisms, eventually leaving 661,899 probes for joined cohort, 662,092 probes for female- and 661,953 for male-specific analysis.

### 2.4 Differential analysis

Differential methylation analysis (DMA) of HBV vaccine responders and non-responders was performed building CpG-wisely linear models with robust regression fitting (*limma* v3.42.2 R package) including chronological age, sex, B cell count and methylation array batch as covariates. Benjamini-Hochberg (BH) procedure was applied as multiple testing correction. CpG sites that reached i) BH-adjusted p-value < 0.05 and ii) the absolute difference between phenotypic group mean β-values (abs(Δβ-values)) above 5%, were considered as differentially methylated positions (DMPs). We performed hierarchical cluster analysis (HCA) with complete-linkage method to assess whether the methylation signature allow to discriminate responder versus non-responder individuals. With dendrogram, we visualized the arrangement of sample clusters and identified the entities with the highest and the lowest similarity. We used principal component analysis (PCA) to evaluate the discriminative/indicative potential of determined DMP signature and to examine its capability to uncover the dissimilarities between R and NR phenotypes. PCA plots were prepared to provide a visual and statistical representation of the weight of the identified differences between groups, beyond the significance of the p-values. We created list of unique differentially methylated genes (DMG) annotating significant CpGs with genes basing on their genomic localization. In order to characterize and functionally profile the DMGs emerged from DMP signature we performed Gene Ontology (GO) enrichment analysis with a public web server g:Profiler (27). We annotated genes with GO terms using *gprofiler2* v0.2.3 R package that accompanies the toolset. In order to summarize and to ease the interpretation of functional results we prepared Manhattan plot with the x-axis denoting the GO pathways and y-axis showing the adjusted enrichment p-values (after negative logarithmic transformation). On the graph, each spot corresponds to a term colored according to the annotation source and the size of the circles reflects the number of respective annotated genes. On the x-axis, the pathways are ordered positioning the related terms (from the same GO subtree) closer to each other. Additionally, we performed pathway enrichment analysis (PEA) in order to identify the biological pathways that were predominantly affected by the epigenetic alterations related to immune response to HBV vaccine. With Enrichr web-based tool (28) we annotated the list of DMGs with frequently occurring pathways using the KEGG database (29) as a reference. We focused on the pathways with Fisher’s exact test p-value < 0.05. We defined as differentially methylated regions (DMRs) those genomic segments which in analysis with Comb-p tool (Python v3.7.6) obtained Sidak-corrected p-values < 0.05 and as extended differentially methylated regions (eDMRs) – the ones with adjusted combined p-value < 0.05 and with at least three significant CpGs per region. We provided methylation box plots to visualize the main results, plotting female non-responders (NR_F) and responders (R_F) next to male non-responders (NR_M) and responders (R_M), in order to evaluate if the trend can be observed in both sexes.

The main outcomes of differential analysis were supplemented with correlation analysis of DNA methylation and anti-HBs titers in studied cohort. We used Pearson’s product-moment correlation approach to measure association between β-values and levels of antibody categorized using thresholds of 10 mIU/ml and 100mIU/ml. Furthermore, principal outcomes were complemented with verification of correlation between DNA methylation and RNA expression in three publicly available datasets (**Table T1** in **Supplementary Data 3**). Results with p-value < 0.05 and absolute value of correlation coefficient > 0.05 were summarized on correlation plots.

## 3 Results

### 3.1 Participants

The median age of the participants at the time of blood collection for B cell isolation was 32 years (interquartile range, IQR 27 – 41), with a median blood cell count of 2.40x10^6^ (IQR 1.60x10^6^ – 3.90x10^6^). Participants were divided into two groups based on their anti-HBs titers: ≥10mIU/ml, defined as responders (R) to the HBV vaccine, and <10mIU/ml, defined as non-responders (NR). The characteristics of each group are summarized in **Table 1**. No statistically significant differences were observed between the groups in terms of sex, average blood cell count, or age when considering both sexes combined or when considering females and males separately. Details on distribution of anti-HBs titers in two responder subgroups, i.e. with low (10-100mIU/ml) and high (10-100mIU/ml) antibody levels, are provided in **Supplementary Table S3** in **Supplementary Data 1**. Results of MDS analysis confirmed that sample randomization was efficient and that possible batch effects were accurately controlled in the studied dataset (**Supplementary Figure S1–S5** in **Supplementary Data 4**).

**Table 1:** Cohort characteristics.

Group characteristics	Anti-HBs titers ≥10 mIU/ml (R)	Anti-HBs titers <10 mIU/ml (NR)	P-value
Number of participants	41	30	n/a
Male and female (%)	14 (34) and 27 (66)	15 (50) and 15 (50)	0.180
Median age	32.12	31.77	0.319
IQR (years)	26.70 – 39.03	26.16 – 45.22	n/a
Median blood cell count	2.10x10^6^	2.75x10^6^	0.333
IQR (cell count)	1.30x10^6^ – 3.40x10^6^	1.88x10^6^ – 4.00x10^6^	n/a
Median anti-HBs titers* (mIU/ml)	143.51	0.00	0.000
IQR (mIU/ml)	52.36 – 546.90	0.00 – 0.00	n/a
Females			
Number of participants	27	15	n/a
Median age	29.61	29.04	0.452
IQR (years)	25.97 – 34.78	24.47 – 42.60	n/a
Median blood cell count	2.35x10^6^	2.35x10^6^	0.708
IQR (cell count)	1.20*10^6^ – 3.43*10^6^	1.83*10^6^ – 3.08*10^6^	n/a
Median anti-HBs titers* (mIU/ml)	137.72	0.00	0.000
IQR (mIU/ml)	49.00 – 480.25	0.00 – 0.00	n/a
Males			
Number of participants	14	15	n/a
Median age	36.92	36.6	0.885
IQR (years)	34.93 – 39.13	29.33 – 44.88	n/a
Median blood cell count	2.10x10^6^	2.80x10^6^	0.277
IQR (cell count)	1.56x106 – 3.40x10^6^	2.23x106 – 4.13x10^6^	n/a
Median anti-HBs titers* (mIU/ml)	260.61	0.00	0.002
IQR (mIU/ml)	69.99 – 705.21	0.00 – 0.00	n/a

* For purpose of this representation antibody levels > 1000mIU/ml were considered as 1000.00mIU/ml.

### 3.2 Analysis of DMPs in R and NR individuals (sex-aggregated analysis)

**Figure 1:**
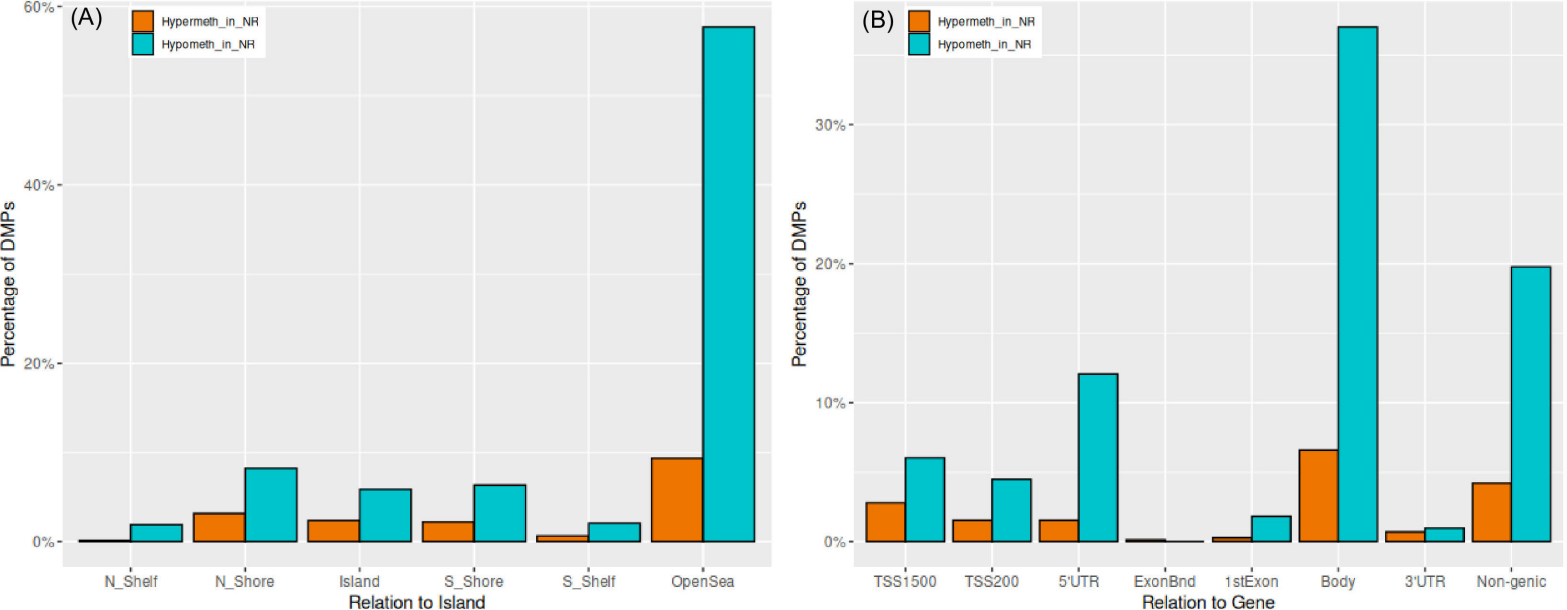
Distribution of hyper and hypo-methylated DMPs in relation to **(A)** CpG islands and **(B)** gene regions. Y-axis indicates percentage of significant CpGs belonging to a particular subregion.

**Figure 2:**
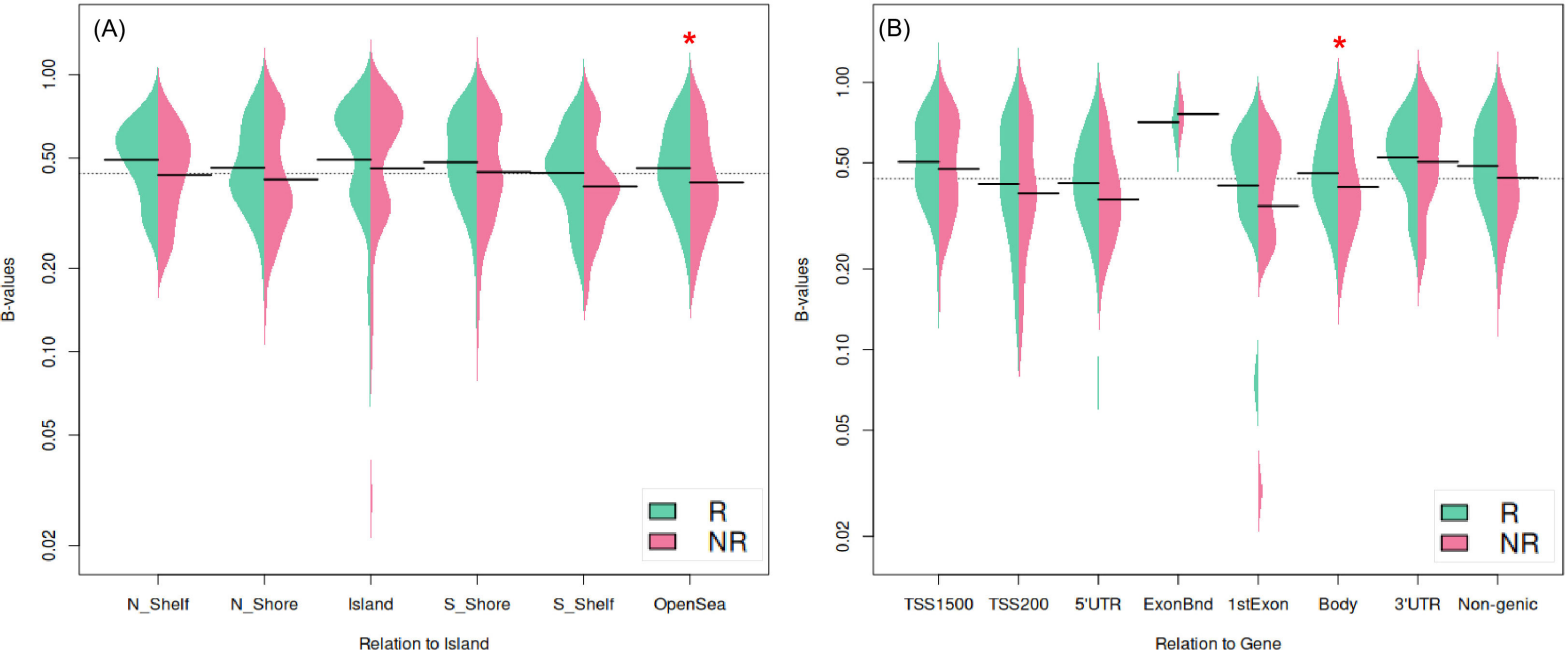
Comparison of methylation β-values distribution of 631 DMPs in R and NR groups represented as split beanplots in relation to **(A)** CpG islands and **(B)** gene regions. Beans’ shape represents density. The Y-axis indicates β-values with the group’s mean shown with short black lines and the overall average for the whole plot - with the dashed black line. Asterisks indicate significant differences between R and NR groups.

Comparing NR and R of the entire study cohort, we found 631 DMPs that were differentially methylated independently of sex (**Supplementary Table S4** in **Supplementary Data 1**; **Supplementary Table S5** in **Supplementary Data 1**). Most (82%) of CpGs were hypomethylated in NR when compared to R as indicated in the volcano plot (**Supplementary Figure S6** in **Supplementary Data 4**). Examination of DMPs distribution demonstrated that the majority (67%) of probes mapped to open sea positions (*i.e.* genomic regions with low CpG density, far from CpG islands and their boundaries, often involved in less explored but potentially important epigenetic mechanisms) with a marked prevalence of hypomethylated DMPs in NR comparing to R (**Figure 1A**). However, a statistically significant difference in DMPs distribution between R and NRs was observed exclusively in the open sea zone (Bonferroni corrected p-value < 0.05; **Figure 2A**).

72% of the DMPs were genic and they mapped to a total of 425 unique DMGs. In relation to gene subregions, the majority of emerged sites was located in gene body including more than 57% of genic DMPs, while remaining 43% were distributed between promoter, 5’UTR and other genomic regions (**Figure 1B**).

Significant DMPs were prevalently hypomethylated in NR group except the border of the exons (*ExonBnd*). The distribution of DMPs between NR and R groups was significantly different in region of gene body (Bonferroni corrected p-value < 0.05; **Figure 2B**), that are often associated with actively transcribed genes, where we found significant hypomethylation in NR compared to R.

None CpG site resulted as significantly associated with unresponsiveness to vaccine in the stratified analyses of probes on X chromosome among females, X chromosome among males and Y chromosome among males.

### 3.3 Analysis of differentially methylated genes and pathways involved

In analyzing the DMPs localized in gene body regions, we identified 333 hypomethylated and 97 hypermethylated DMGs in the NR group compared to the R group.

**Figure 3:**
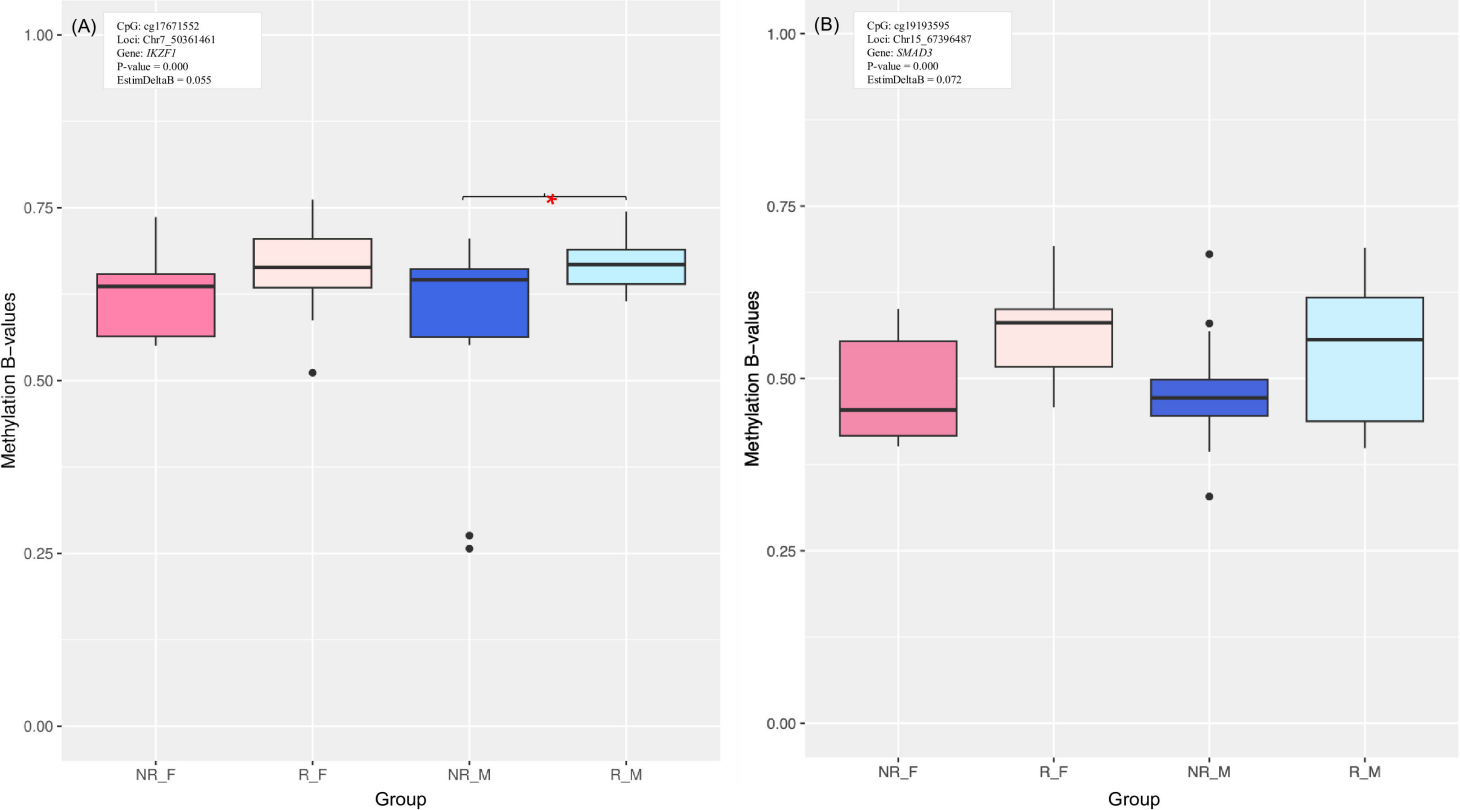
Methylation box plot of **(A)** cg17671552 CpG site within IKZF1 gene and **(B)** cg19193595 CpG site within SMAD3 gene. Asterisks indicate significant differences between R and NR groups.

Top 8 of emerged DMPs are visualized in **Supplementary Figure S7** in **Supplementary Data 4**. All of these CpGs were hypomethylated in NR group. Interestingly, among them, we found *IKZF1* (**Figure 3A**; results of complementary correlation analysis in public datasets are provided in **Supplementary Data 3: Figures T1 – T2**), which was recently reported as a key protein for lymphoid differentiation and protection against pathogens (30, 31). The significant cg17671552 was located in the gene body, in a CpG poor region. It was hypomethylated in NR group of studied cohort and it showed positive correlation between DNA methylation and RNA expression levels in public dataset GSE181647. This DMP remained significant in male- but not in female-specific analysis. Methylation at *IKZF1* (cg17671552) was correlated with anti-HBs titers in joined and female cohort (**Supplementary Table S6** in **Supplementary Data 1**).

Moreover, DMG analysis indicated that the *SMAD3* gene (*SMAD* Family Member 3), more precisely cg19193595 position, was significantly hypomethylated in NR compared to R in our cohort whilst the data on correlation between DNA methylation and RNA expression retrieved from public datasets was not available for this CpG site (**Figure 3B**; results of complementary correlation analysis in public datasets are provided in **Supplementary Data 3: Figure T3**). Methylation at *SMAD3* (cg19193595) was correlated with anti-HBs titers in joined and female cohort (**Supplementary Table S6** in **Supplementary Data 1**).

*SMAD3* encodes a protein that plays a crucial role in the *TGF-β* (transforming growth factor-beta) signaling pathway, which regulates a wide variety of cellular processes, including proliferation, differentiation and apoptosis, that impact the immune system functions (32). Methylation box plots for aggregated sexes are provided in **Supplementary Figure S8** in **Supplementary Data 4**.

**Figure 4:**
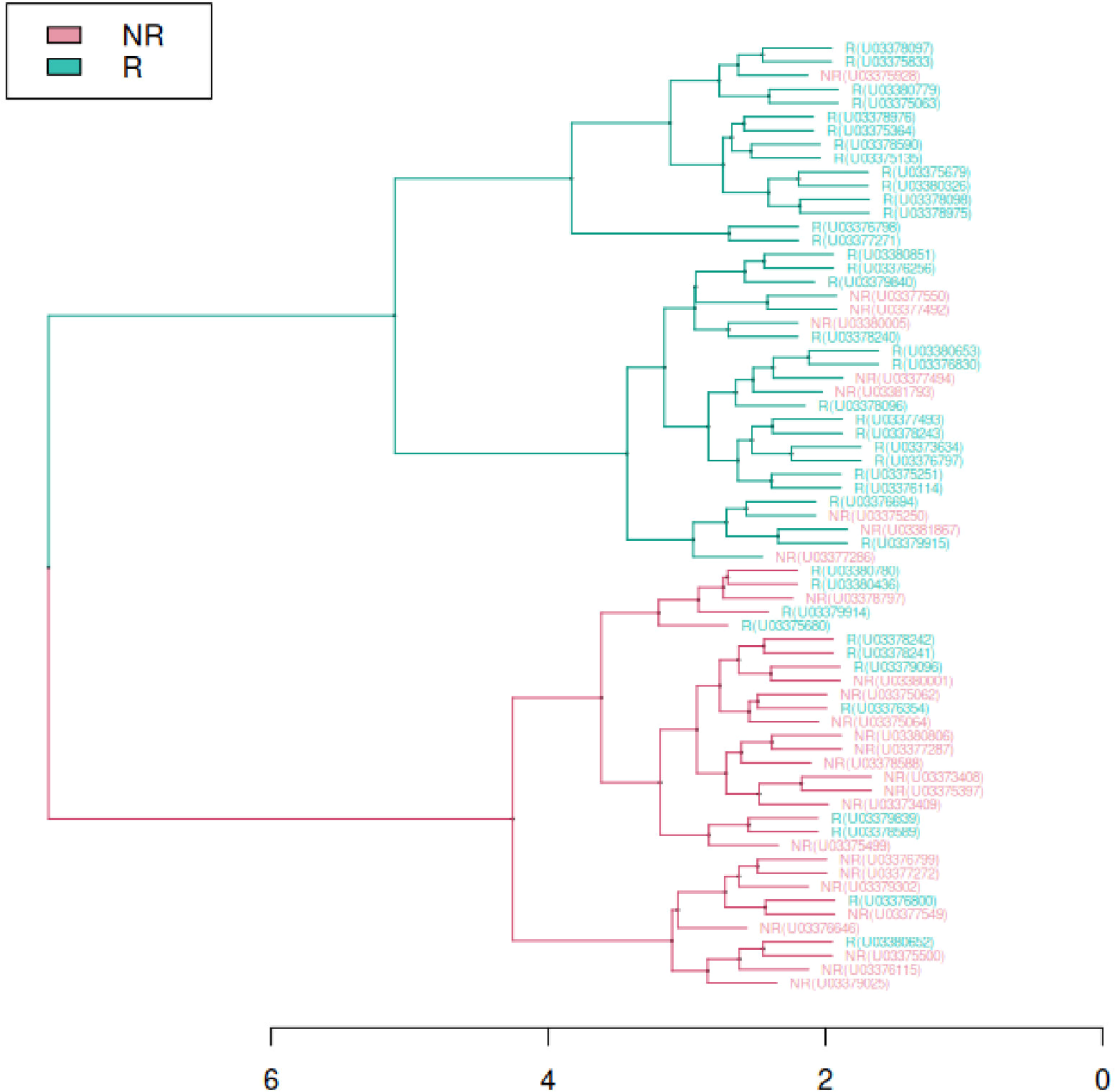
Clustering with complete agglomeration method using normalized β-values of 631 DMPs emerged as significantly differentially methylated between R (green labels) and NR (red labels) in sex-joined cohort.

Cluster analysis using differentially methylated CpGs resulted in two principal branches that divided samples according to the phenotype (**Figure 4**). The first dendrogram branch grouping R individuals correctly agglomerated 70% of samples, mislabeling 9 NR, and the second branch corresponding to the NR class correctly identified 68% of samples. Data exploration with PCA uncovered the presence of two outliers (two NR males) that showed values more than 3 standard deviations away from the mean in at least one of the first two components. These samples were excluded from further PCA visualization. Using DMP signature, we obtained a moderate separation of two phenotypes (**Supplementary Figure S9** in **Supplementary Data 4**), with the first component (PC1) explaining slightly more than 52% of data variability allowed to group NR samples on more right side of the plot and shift R samples to the left. The contribution of a second component (PC2) was much more reduced since it captured only around 3% of data variability therefore the separation along y axis resulted much less pronounced. This analysis suggests that R and NR distribute as two separated groups according to the CpG methylation pattern.

**Figure 5:**
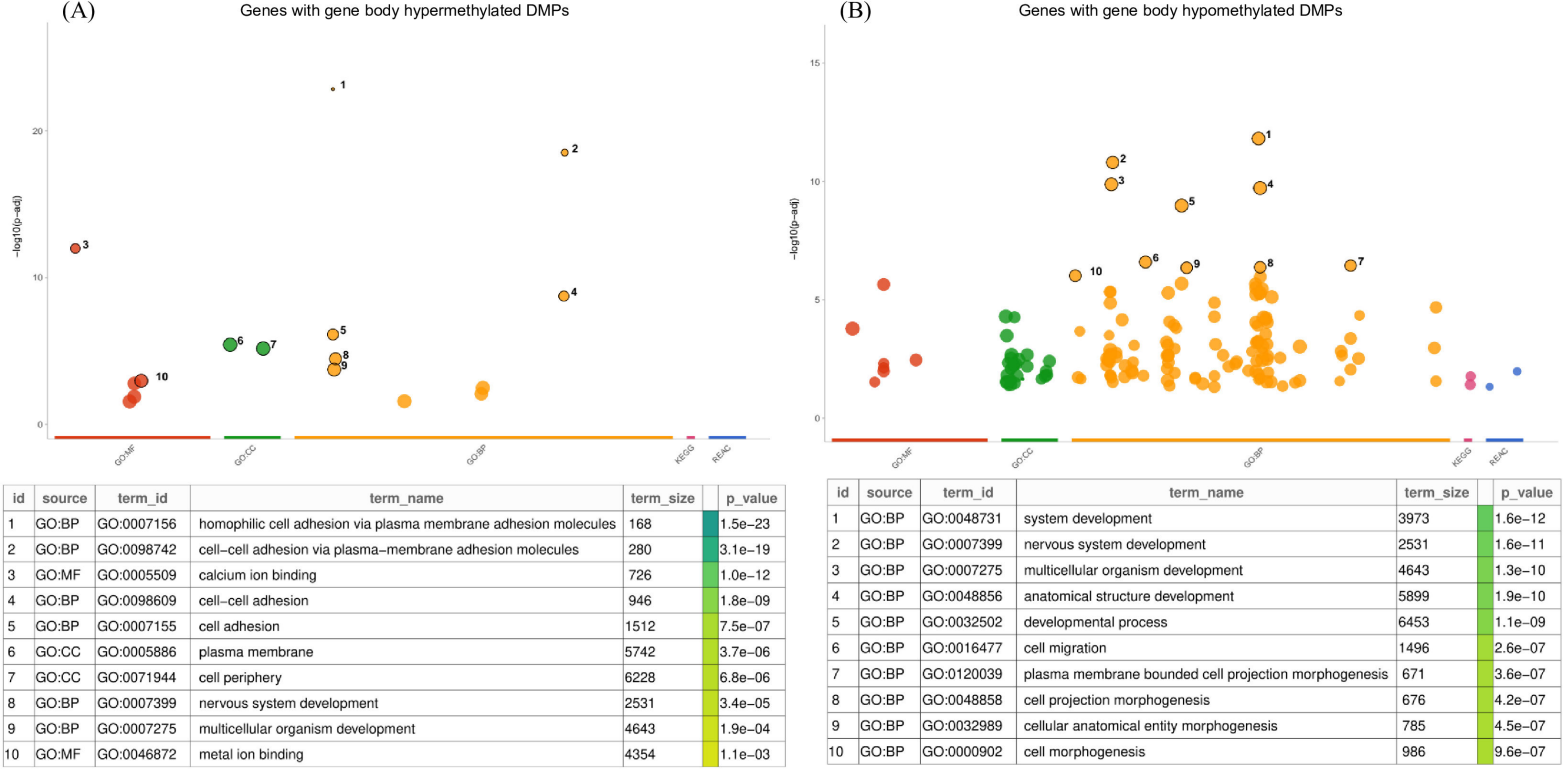
Gene ontology analysis of genes mapped to significantly differentially methylated sites located in gene body with **(A)** hypermethylated and **(B)** hypomethylated status in NR comparing to R group. Dot colors correspond to different annotation classes: red, GO Molecular Function (GO: MF); green, GO Cellular Component (GO: CC); yellow, GO Biological Process (GO: BP); pink, KEGG database; and blue, Reactome database. Numeric dot labels link dot representation with respective significant pathway listed in attached table. Continuous green scale corresponds to statistical significance where darker to brighter colors reflect increasing p-vales.

In order to characterize the function of genes that emerged from CpG-wise DMA, we performed gene ontology (GO) and pathway enrichment analyses. For GO analysis, we used two lists of unique genes: higher DMGs (n=97) and lower (n=333) DMGs in NR group compared to R, according to the methylation change observed in significant CpGs. 5 genes were common to both categories since they mapped to hyper- as well as hypo-methylated DMPs. These were proteins belonging to the protocadherin gamma family: *PCDHGA1*, *PCDHGA2*, *PCDHGA4*, *PCDHGB1* and *PCDHGA3*. As summarized in **Figure 5**, the genes with hypermethylated DMPs located in the body were mainly associated with cell adhesion (**Figure 5A**), while DMGs with hypomethylated sites were linked to development (**Figure 5B**). In PEA we annotated unique list of 425 DMGs with KEGG pathways and according to results genes with differentially methylated CpGs enriched pathways of “Th17 cell differentiation” (p-value = 0.000; adjusted p-value = 0.110), “Human papillomavirus infection” (p-value = 0.002; adjusted p-value = 0.197) and “Hepatitis B” (p-value = 0.002; adjusted p-value = 0.197) (**Table 2**).

**Table 2:** Top 10 results of pathway enrichment analysis of unique genes mapped to significantly differentially methylated sites.

#	Term	Overlap	P-value	Adjusted P-value	Combined Score	Genes
1	**Th17 cell differentiation**	9/107	**0.000**	0.110	33.004	*SMAD3;STAT1;IL23R;TBX21;IL21R;PRKCQ;CD3E;HIF1A;RUNX1*
2	**Human papillomavirus infection**	16/331	**0.002**	0.197	14.821	*RB1;ITGB1;WNT5B;STAT1;NOTCH4;IFNA2;ITGA1;PIK3R1;PRKCZ;FOXO1;CDK6;PKM;CASP3;CREB3L2;ITGA5;ATP6V0A1*
3	**Hepatitis B**	10/162	**0.002**	0.197	18.460	*RB1;SMAD3;CASP10;STAT1;CASP3;CREB3L2;IFNA2;BCL2;TAB2;PIK3R1*
4	AGE-RAGE signaling pathway in diabetic complications	7/100	0.005	0.263	18.265	*SMAD3;STAT1;CASP3;BCL2;PIK3R1;PRKCZ;FOXO1*
5	Chronic myeloid leukemia	6/76	0.006	0.263	20.720	*RB1;BCR;SMAD3;CDK6;PIK3R1;RUNX1*
6	Measles	8/139	0.010	0.320	13.170	*CDK6;STAT1;CASP3;IFNA2;BCL2;TAB2;PIK3R1;CD3E*
7	Epstein-Barr virus infection	10/202	0.011	0.320	10.879	*RB1;NCOR2;CDK6;STAT1;CASP3;IFNA2;BCL2;TAB2;PIK3R1;CD3E*
8	Inflammatory bowel disease	5/65	0.012	0.320	17.025	*SMAD3;STAT1;IL23R;TBX21;IL21R*
9	Proteoglycans in cancer	10/205	0.013	0.320	10.481	*ITGB1;CTTN;WNT5B;CASP3;RRAS2;ANK2;PIK3R1;ITGA5;ANK1;HIF1A*
10	Small cell lung cancer	6/92	0.014	0.320	13.926	*ITGB1;RB1;CDK6;CASP3;BCL2;PIK3R1*

The three most significant texts (p-values <0.005) are indicated in bold.

### 3.4 Analysis of differentially methylated regions

From the region-oriented analysis emerged a total of 4,362 DMRs of which 2,862 had adjusted combined p-value < 0.05 and at least three significant CpGs within the region (median 4; in range between 3 and 22) (**Supplementary Table S7** in **Supplementary Data 1**). eDMRs encompassed a total of 14,854 methylation sites mapping to 3,155 unique genes. 74 of eDMR-linked CpG sites were previously identified during site-wise analysis. The length of extended significant regions was 240.455 ± 146.392 bases on average, ranged between 6 and 1200 bases and had non-normal distribution (Shapiro-Wilk test p-value < 0.05). There were 3,143 hypomethylated and 600 hypermethylated eDMR genes in NR compared to R. 588 genes were common for both lists, depending on the location of the CpG. The genic region encompassing the highest number of methylation sites was located on chromosome 13 between 78,493,012 and 78,494,011 position (hg19). It mapped to CpG island and its S shore within *EDNRB* gene (not emerged previously from DMP analysis), coding endothelin receptor type B and reported as involved in liver injury and pro-inflammatory responses (33).

### 3.5 Analysis of methylation patterns in female NR and R individuals

Among the females, there were 194 differentially methylated positions (DMPs) between NR and R individuals (**Supplementary Table S4** in **Supplementary Data 1**; **Supplementary Table S8** in **Supplementary Data 1**). 65% of CpGs were hypermethylated in NR compared to R as indicated in the volcano plot (**Supplementary Figure S10** in **Supplementary Data 4**). General statistics on distribution of emerged DMPs in relation to CpG island and to gene architecture are provided in **Supplementary Figure S11** in **Supplementary Data 4**. 70% of all DMPs were genic, and they were spread over 147 unique genes, of which 93 were hypermethylated and 55 were hypomethylated in NR compared to R individuals.

**Figure 6:**
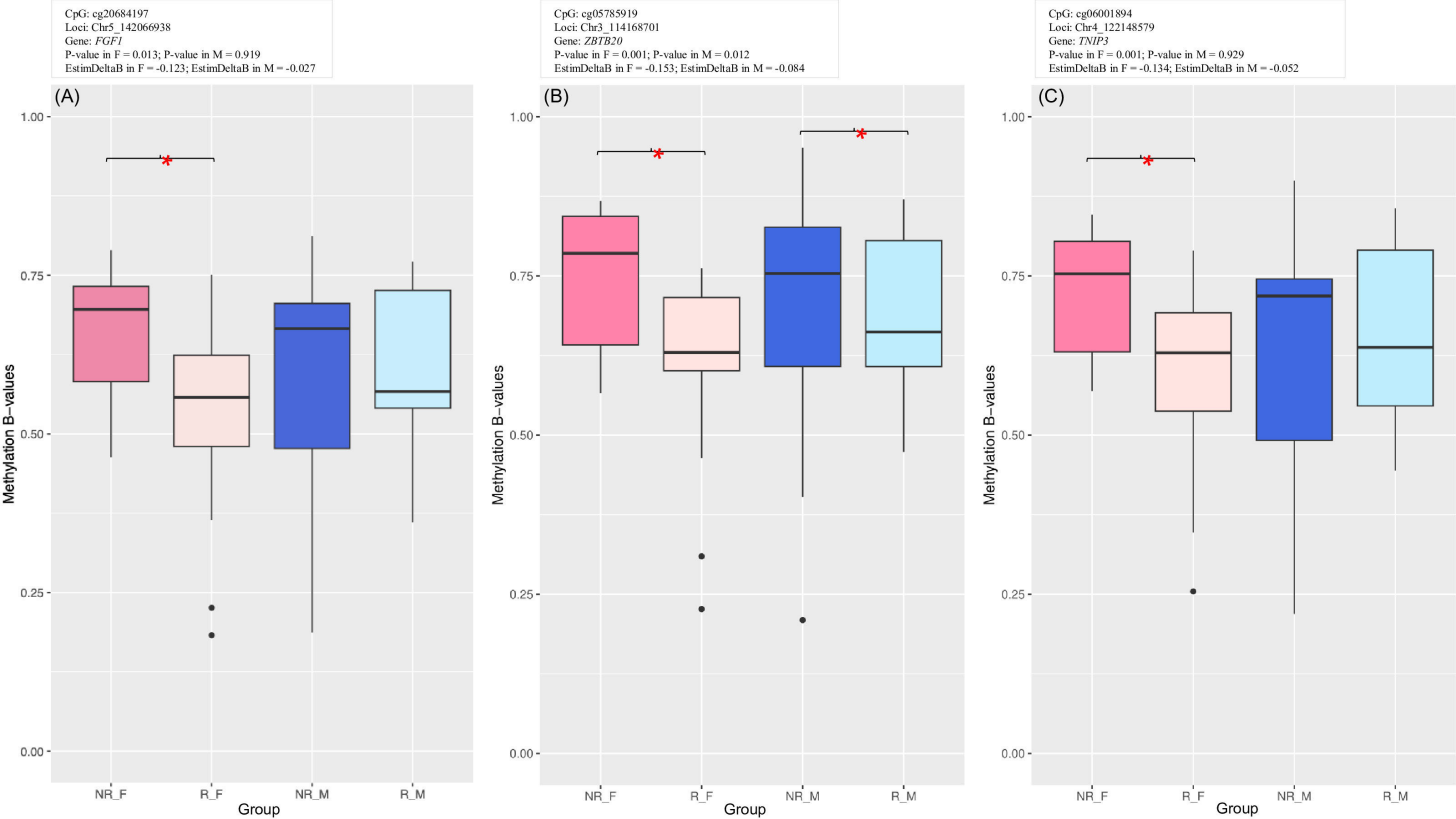
Methylation box plots of top genic CpG sites from female-specific analysis: **(A)** cg20684197 (*FGF1*), **(B)** cg06001894 (*ZBTB20*), **(C)** cg10390905 (*TNIP3*). Males are included in the box plots to evaluate whether the similar trend occurs also in this sex group. Asterisks indicate significant differences between R and NR groups.

Among the top (adjusted p-value < 0.02 and abs(Δβ-values) > 0.12) DMP-linked genes were *FGF1*, *ZBTB20*, and *TNIP3* (**Supplementary Table S8** in **Supplementary Data 1**), all three involved in reducing liver inflammation and disease (34–36). Moreover, *ZBTB20* was found highly expressed in murine germinal center and memory B cells (37). **Figure 6** visualizes DNA methylation in these three DMPs (results of complementary correlation analysis in public datasets are provided in **Supplementary Data 3: Figures T4–T20**). Males are included in the box plots to evaluate whether a similar trend occurs also in this sex group. Methylation β-values at *ZBTB20* (cg06001894) and *TNIP3* (cg10390905) were correlated with anti-HBs titers in female cohort (**Supplementary Table S6** in **Supplementary Data 1**).

We identified 44 DMRs with adjusted combined p-value < 0.05 (**Supplementary Table 9** in **Supplementary Data 1**) but none of them had three or more significant CpGs within the region. These DMRs encompassed 51 CpGs and 33 unique genes (9 hyper- and 24 hypomethylated in NR). 12 of CpGs and 10 of genes overlapped with the results of site-wise DMA.

### 3.6 Analysis of methylation patterns in male NR and R individuals

**Figure 7:**
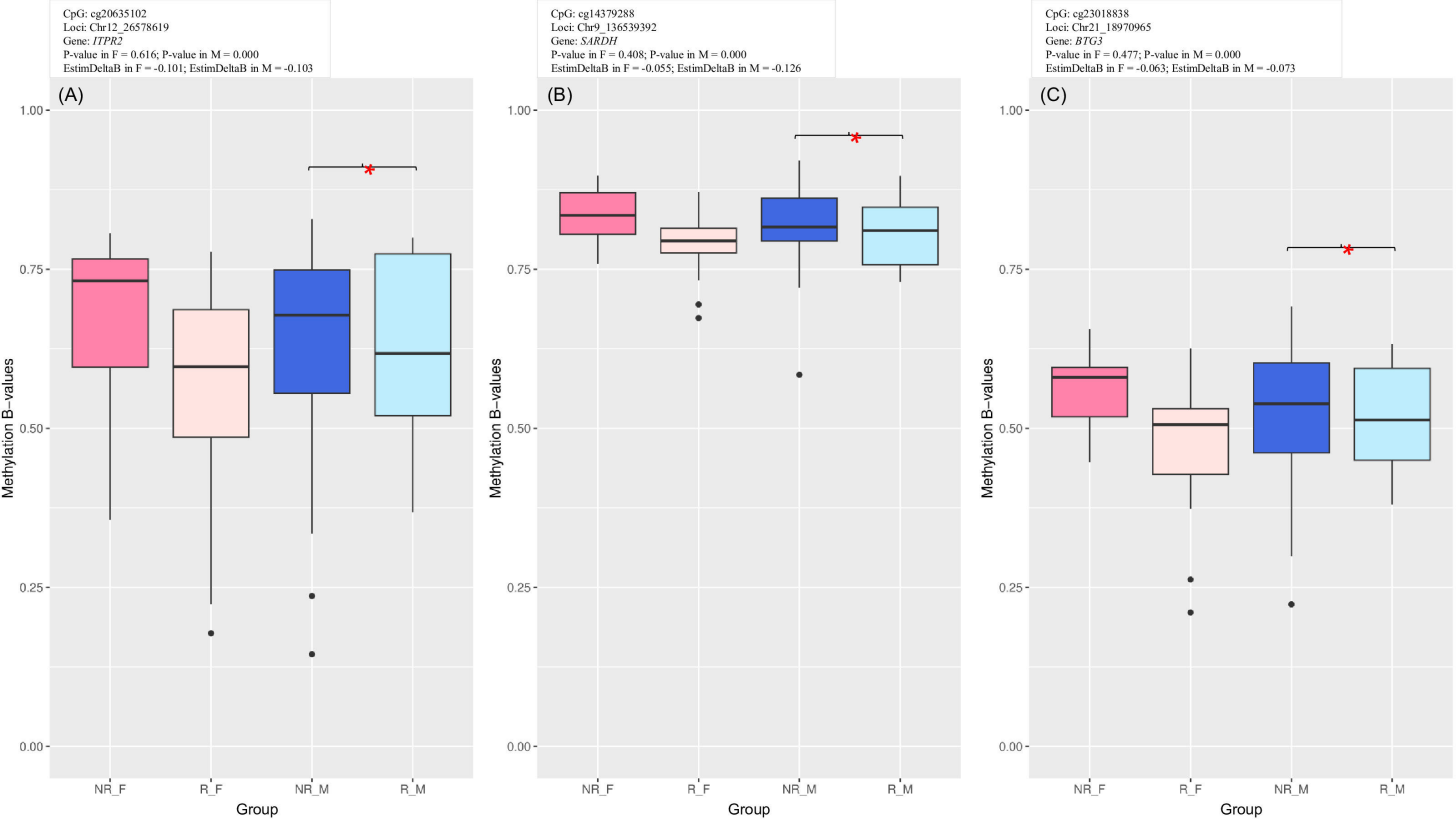
Methylation box plots of top genic CpG sites from male-specific analysis: **(A)** cg20635102 (*ITPR2*), **(B)** cg14379288 (*SARDH*), **(C)** cg23018838 (*BTG3*). Females are included in the box plots to evaluate whether the similar trend occurs also in this sex group. Asterisks indicate significant differences between R and NR groups.

In the male cohort, we found 2199 DMPs (**Supplementary Table S4** in **Supplementary Data 1**; **Supplementary Table S10** in **Supplementary Data 1**) of which 65% were hypermethylated in NR comparing to R as indicated in the volcano plot (**Supplementary Figure S12** in **Supplementary Data 4**), comparably to female cohort. General characterization of emerged DMPs in relation to CpG island and to gene architecture are provided in **Supplementary Figure S13** in **Supplementary Data 4**. 68% of identified CpGs were genic and mapped to 1364 unique genes (932 hypo- and 488 hypermethylated). The top genes that emerged from male-specific DMA were *ITPR2*, *SARDH*, and *BTG3*. *ITPR2* was reported to play a protective role in liver damage and to modulate cell senescence (38, 39); *SARDH* was recently shown to regulate immune cell infiltration in hepatocellular carcinoma (HCC) and to promote HCC progression (40). *BTG3* expression was associated with cellular senescence (41). **Figure 7** visualizes DNA methylation in these three DMPs (results of complementary correlation analysis in public datasets are provided in **Supplementary Data 3: Figures T21–T23**). Females are also included in the box plots to evaluate if the trend is reproduced in this sex group. Methylation β-values at *SARDH* (cg14379288) and *BTG3* (cg23018838) were correlated with anti-HBs titers in female but not male cohort (**Supplementary Table S6** in **Supplementary Data 1**).

DMR analysis returned 551 significant regions, but only 24 had adjusted combined p-value < 0.05 and at least three significant CpGs within the region (**Supplementary Table S10** in **Supplementary Data 1**). These eDMRs encompassed 112 CpGs and 24 unique genes (6 unequivocally hypermethylated in NR compared to R, 16 unequivocally hypomethylated, and 2 bidirectional). 44 of CpG sites discovered with eDMRs were common with DMP list and 20 genes overlapped with results of site-oriented male-specific DMA. The genic DMR encompassing the highest number (4) of CpGs was located on chr8: 21771252 – 21771447 (hg19) mapping to the S shore of *DOK2* gene, which regulates the development and function of natural killer cells - important players in the immune response (42).

### 3.7 Common differentially methylated genes between aggregated and sex-specific analysis

We verified the overlaps between gene lists resulting from aggregated and sex-specific DMP analysis as visualized in **Supplementary Figures S14, S15** in **Supplementary Data 4**. According to up-to-date literature, the 11 genes which were common between three settings have not been previously associated to immune response to vaccines (**Table 3**), except for SMAD3 whose inhibition has been found to improve humoral response to HBsAg DNA vaccination (43).

**Table 3:** Genes common between results obtained from joined and sex-specific DMP analysis.

Gene	Sex-joined analysis				Female-specific analysis				Male-specific analysis			
	CpG	Δβ	P-value	Adj. P-value	CpG	Δβ	P-value	Adj. P-value	CpG	Δβ	P-value	Adj. P-value
*AUTS2*	cg15503762	0.062	0.000	0.004	cg00980636	-0.058	0.000	0.045	cg05081041	-0.079	0.000	0.005
*FOXN3*	cg12504912	0.099	0.000	0.002	cg13663980	-0.087	0.000	0.027	cg10932086	-0.074	0.000	0.000
*HIVEP3*	cg26038582	0.060	0.002	0.025	cg23762517	0.087	0.000	0.025	cg06958720	-0.115	0.000	0.015
*PCDHGA1* *PCDHGA2* *PCDHGA3* *PCDHGA4* *PCDHGB1* *PCDHGB2*	cg00395420	0.053	0.000	0.003	cg03086707	0.059	0.000	0.002	cg02452944	-0.069	0.000	0.017
	cg27079776	0.059	0.000	0.009								
	cg01224715	-0.058	0.004	0.038								
*SETD8*	cg11628550	-0.077	0.000	0.005	cg11628550	-0.087	0.000	0.046	cg11628550	-0.102	0.000	0.002
*SMAD3*	cg23731272	0.080	0.003	0.034	cg20110851	-0.115	0.000	0.025	cg10711190	-0.079	0.000	0.016
	cg19193595	0.072	0.000	0.000					cg15291389	0.059	0.000	0.000
	cg02486855	0.052	0.004	0.038								

The differential methylation signal found in aggregated and sex-specific DMA was concordant for the common genes except for *HIVEP3* which involved significantly NR-hypomethylated CpG in joined and in female cohorts, whereas it presented NR-hypermethylated CpG in the separated male group.

146 loci corresponding to 102 unique genes were previously found as differentially methylated between responders and non-responders to HBV vaccine in whole blood samples (44). Even though none of the most significant CpG sites that emerged from sex-joined or sex-specific analysis in our cohort were found by Lu Y and collaborators (44), there were 10 unique differentially methylated genes shared between the two studies (**Table 4**). The direction of methylation alteration was concordant between two studies only for *ERICH1*, *FGFR2* and *SDHAP3* genes.

**Table 4:** Genes common between results obtained from sex-aggregated DMP analysis and genes emerged from Lu et al. study (13).

#	Gene	Sex-joined analysis		Lu et al., 2014 (13)	
		CpG	State in NR	CpG	State in NR
1	*ALOX12*	cg21237687; cg03407747	Hypomethylated	cg01600516	Hypermethylated
2	*ATXN7L1*	cg10575219	Hypomethylated	cg16963093	Hypermethylated
3	*CCDC154*	cg04837170; cg10681552; cg24070867; cg22171016	Hypermethylated	cg06352616	Hypomethylated
4	*ERICH1*	cg22307444	Hypomethylated	cg17373649	Hypomethylated
5	*FGFR2*	cg16653991; cg08899523	Hypomethylated	cg02210151	Hypomethylated
6	*KALRN*	cg04915618	Hypomethylated	cg06690085	Hypermethylated
7	*LRRC16A*	cg14639704	Hypomethylated	cg19938535	Hypermethylated
8	*RIN3*	cg17366276	Hypomethylated	cg01022501	Hypermethylated
9	*SDHAP3*	cg21931717	Hypomethylated	cg21167402; cg08778598; cg26472636	Hypomethylated
10	*TRIO*	cg24148874	Hypomethylated	cg17013691	Hypermethylated

Owing to the high dimension of the methylation array is given that some degree of false positive and false negative hits has to be considered among the presented results. However, the biological concordance of most of the results, the overlap with previous and independent studies, and the use of separated B cells encourage us to support the idea that many of our highlights represent promising target for future studies on HBV immunization in humans.

## 4 Discussion

Understanding the factors underlying impaired HBV vaccine response is crucial to improving vaccine immunogenicity and efficiency and optimizing immunization strategies.

Many studies have attempted to correlate the response to the HBV vaccine with various factors, including age and sex (16, 45–47). However, little is known about epigenetic mechanisms influencing vaccine response.

Here, we present a pioneering genome-wide DNA methylation study in isolated B cells from responders and non-responders to HBV vaccine. The studied cohort was recruited from healthcare workers at high risk for infection and priority category for vaccination. We performed differential methylation analysis on CpG and region levels, describing the differential signal in relation to CpG island and gene architecture. We demonstrated that responders and non-responders are characterized by different epigenetic signatures which have the indicative potential to discriminate to some extent between R and NR groups. Based on these signatures, we have identified genes that were not previously reported to be associated with HBV vaccine responsiveness. We complemented this analysis with an exploration of pathways enriched in emerged genes. Additionally, the numerosity of the studied cohort allowed us to identify and describe, for the first time, sex-specific DNA methylation differences associated with the response to the HBV vaccine. Interestingly, the genes emerging from this analysis of epigenetic signatures appear to be specific for females and males.

Our results, derived from both aggregated and sex-specific analysis, showed that 11 genes were differentially methylated between R and NR to the HBV vaccine. Specifically, *AUST2*, *FOXN3*, *HIVEP3*, *SET8*, and *SMAD3* are inflammation- and immune-related genes. AUST2 has recently been identified as a reliable molecular marker of inflammatory status and it has been included in a reliable molecular predictive model for inflammation to differentiate between subjects with and without inflammation (48). Phosphorylation and subsequent proteasomal degradation of *FOXN3* have been found to play a crucial role in lung inflammation and injury in a murine model of bacterial infection (49).

*HIVEP3* is a transcription factor involved in regulation of inflammatory response (50). Placental DNA methylation level of three loci in the *HIVEP3* gene shore region was found associated with pro-inflammatory cytokine *TNFα* protein levels in cord blood (51). *SETD8* was shown to be involved in the progression of inflammatory bowel disease (52). *SMAD3* acts as a signaling molecule within the *TGF-β* pathway. *TGF-β/SMAD3* signaling plays a significant role in regulating immune responses by modulating the differentiation and activation of immune cells (32). Furthermore, a study demonstrated that inhibiting *TGF-β/SMAD3* signaling using praziquantel as an adjuvant enhanced both humoral and cellular responses to hepatitis B surface antigen (HBsAg) DNA vaccination. This inhibition led to increased T cell proliferation and higher specific antibody responses, suggesting that targeting *SMAD3* can improve vaccine-induced immunity (43).

The pathway enrichment analysis results confirmed that DMGs correlated with unresponsiveness to HBV vaccine are involved in immune cell differentiation, have been found to be altered during inflammatory diseases, or are related to processes that are important to ensure an adequate immune response. For example, proteoglycans which are key constituents of the extracellular matrix, have been shown to bind and regulate the production and function of several cytokines, chemokines, and growth factors, as well as guide the movement and positioning of leukocytes (i.e., monocytes/macrophage, B and T lymphocytes) (53, 54). Many genes whose expression is altered during viral infections are part of pathways that are also crucial for the immune response to vaccines (55–57). Among them are several genes that modulate the cell cycle, cell proliferation and apoptosis (i.e. *CASP10*, *CASP3*, *BCL2*, *CDK6*); genes that code for cytokines (i.e. *IFNA2*); proteins of signaling pathways responsible for the development, activation and function of immune cells (i.e. *FOXO1*, *STAT1*, *PIK3R1*) (58). Taken together, these results suggest that specific modifications in DNA methylation profiles could be associated with a poor immune response to HBV vaccination.

In order to better understand the significance of differentially methylated genes associated with a poor response to the HBV vaccine, we compared our findings with those of previous studies. Upon reviewing the most recent literature, we found that none of the 11 DMGs between R and NR to the HBV vaccine were mentioned. It is worth noting that nearly all studies that investigated DNA methylation profiles in response to vaccination used whole blood samples or peripheral blood mononuclear cells (PBMCs). Therefore, it is likely that the heterogeneous cell composition of whole blood and PBMCs with respect to isolated B cells can account for the discrepancy between our and previously published results.

However, we observed 10 genes (reported in **Table 4**) that were previously associated with unresponsiveness to HBV vaccination in a study conducted in whole blood by Lu Y et al. (44). In particular, four of them that emerged from sex-aggregated analysis (*ALOX12*, *FGFR2*, *SDHAP3* and *TRIO*), have been found to modulate inflammation (59–63). Aberrant *ALOX12* methylation has been correlated with several diseases characterized by low-grade chronic inflammation, such as atherosclerosis, diabetes, and obesity (59). *FGFR2* signaling has been found to suppress skin inflammation (64). Furthermore, several studies have reported the role of *FGFR2* signaling in modulating the immune response to infections with viruses (65–68). These observations reinforce what we found in a previous work, that is, an association between HBV vaccine unresponsiveness and methylation level of a marker of chronic low-grade inflammation (13).

Recent studies have suggested that sex has a strong influence on DNA methylation, which contributes to sex differences in cell and organ development, function, and susceptibility to specific diseases (69–72). The immune responses of females and males are different (15, 16) and the underlying mechanisms are distinct as well. However, the attempt to identify these mechanisms at the epigenetic level of methylation patterns and to determine if they have sex-specific or shared character has not been taken so far.

To our knowledge, this study is the first to identify sex-related DNA methylation patterns characterizing non-responders to the HBV vaccine. We identified 147 DMGs in females and 1364 DMGs in males (as well as number of unique genes in hypermethylated DMPs: 93 vs 932 respectively, and number of unique genes in hypomethylated DMPs: 55 vs 488 respectively) suggesting that DNA methylation differences between responders and non-responders are greater in male vaccinated HCWs than in female ones. However, this difference can be explained by the greater epigenetic heterogeneity in females that we have already observed and described in previous articles (13, 14). The higher entropy system of females penalizes the search for DMPs and DMRs in group comparisons. It is interesting to note, however, that the gene-specific signals of greatest interest have the same trend in both sexes. This leads us to speculate that the regulation of the genes that have been highlighted can play a significant role in the response to HBV vaccination.

Therefore, in contrast to the epigenetic mechanisms of immune response mentioned above, which are shared between the sexes, apparently there are also sex-specific processes. Among the genes found to be the most differentially methylated in females NR compared to sex-matched R, we observed *ZBTB20*, a transcription factor highly expressed in germinal centers and memory B cells, and which was shown to be required for long-term survival of plasma cells and antibody (6, 73).

Some male-specific DMGs were found to be correlated with cellular senescence. *ITPR2*, which is hypermethylated in male NR compared to R, is a calcium-release channel that has been shown to promote immunosenescence in studies conducted in murine models (39). *BTG3* depletion induces senescence in human fibroblasts by enhancing the expression of p16INK4a (41).

Our findings suggest a role of methylation-mediated regulation of B cell senescence in male unresponsiveness to the HBV vaccine. This conclusion is consistent with our previous research that showed an association between a poor immune response to the HBV vaccine and an increased biological age of B cells measured by DNAm-based estimates (13, 14).

Complementary analysis of three publicly available datasets which the most fitted reported here experimental settings, confirmed the presence of significant correlations between DNA methylation and RNA expression in genes described above. These correlations were found despite the several limitations of outsourced data (i.e. very low sample sizes, only female samples, outdated arrays) and not complete correspondence between study designs due to sex/age distribution mismatches, different tissues, incompatible methylation arrays. Integration of methylomic and transcriptomic data is crucial to draw accurate conclusions however it must be remembered that interactions between methylation and expression are complex and go beyond the direct standard correlations (74).

The main strength of this study lies in the fact that it provided for the first time an in-depth analysis of DMGs of purified B cells from both male and female responders and non-responders to the HBV vaccine. This experimental approach helps generate high-quality data with minimal noise impact compared to whole blood or PBMCs. Reinius and collaborators provided the evidence on the distinct methylation profiles of separated blood cell lineages and emphasized the importance of shifting the experimental strategy to isolated cell subpopulations (75). Cells tend to cluster by their type rather than according to the donor, with B cells demonstrating the most divergent methylation profile from other lymphocytes. Additionally, using isolated B cells in our study allowed us to set the threshold of significant group differences in methylation levels to 5% (results of effect size analysis are provided in **Supplementary Data Sheet 5**). However, the methylation analysis of purified B cells is cost-consuming compared to whole blood/PBMC analysis and does not allow for the analysis of a large number of samples. RNA-seq experiment was not included in this study therefore functional interpretation of identified genes remains speculative. However, for main emerged genes we have found correlations between CpG methylation and RNA expression in three publicly available datasets. In the future, it will be important to increase the number of male and female samples collected and extend the analysis to gene expression to understand the mechanisms underlying sex-specific responses to the HBV vaccine. Furthermore, given that many confounding factors (e.g. BMI, smoking) may strongly impact the immune response, future studies should take them into consideration to allow adequate immunological interpretation of results. In conformity with Italian law, as the mandatory HBV vaccination may be provided any of several products which are officially approved by the Italian Medicines Agency and the administration is made according to the choice of the regional health system. This fact could rise considerations for dataset variability regarding the primary vaccine type, composition and/or dosages and adequate adjustment of the analysis. However, the direct evidence that specific adjuvants cause distinct DNA-methylation signatures is currently unavailable and mostly indirect. Most epigenetic vaccine studies measure methylation changes after vaccination without isolating the contribution of the adjuvant from the antigen or the immune response itself. Moreover, these concerns can be ruled out since the adjuvants differ slightly between the hepatitis B monovalent vaccines (Engerix B and HBVAXPRO) and the hexavalent pediatric combination vaccines, but they are all aluminum-based adjuvants (alum). In addition, all the HBV vaccine in use in Italy are recombinant vaccines produced in yeast, so they have homogeneous composition and are administered withe the same schedule.

Several studies have previously demonstrated presence of the epigenetic differences between high and low vaccine responders (76, 77). In our cohort, the suggested analysis was not feasible since there are only 5 individuals with peak antibody levels. However, we found significant correlation between anti-HBs titers and DNA methylation as reported in **Supplementary Table S6**, which could suggest that observation of such differences (peak vs wanning levels) could be expected in a larger cohort.

In conclusion, we demonstrated significantly different methylation patterns and identified differentially methylated genes between male and female responders and non-responders to HBV vaccination. Overall, our findings provide additional evidence on the possible contribution of DNA methylation to sex-specific immune response to HBV vaccination. The present manuscript reports the results of a cross-sectional study on whole genome DNA methylation analysis of separated B cells. Even though the separated B cells can contain some impurity in terms of cells belonging to different population, thus influencing some of the presented result, the isolated cells allow to remove molecular noise owing to mixed cell types such as in whole blood. This open to a clear view on DNA methylation arrangement in responder and non-responder to HBV vaccination. This is an observational study providing interesting clues on human immunization and, to our knowledge, is the first report in literature with such characteristic, not only in HBV vaccination but in vaccination in general. Owing to this peculiarity the here reported differentially methylated genes could be taken into consideration for future research on sex-specific differential response to HBV vaccine to assess whether these differences are marker of response or also involved in the mechanism of the HBV immunization.

## Data Availability

The datasets generated and analyzed during the current study are available in the GEO NCBI repository accessible through GEO Series accession number GSE273657 (https://www.ncbi.nlm.nih.gov/geo/query/acc.cgi?acc=GSE273657).
