# Supplementary material for: B-cell DNA methylation signature in response to hepatitis B virus vaccination in females and males
Source: Front Immunol. 2026 Apr 10;17:1734384. doi: 10.3389/fimmu.2026.1734384 (PMC13105942; doi:10.3389/fimmu.2026.1734384)
Supplement: Supplementary file 2 [file DataSheet2.pdf]

Beta

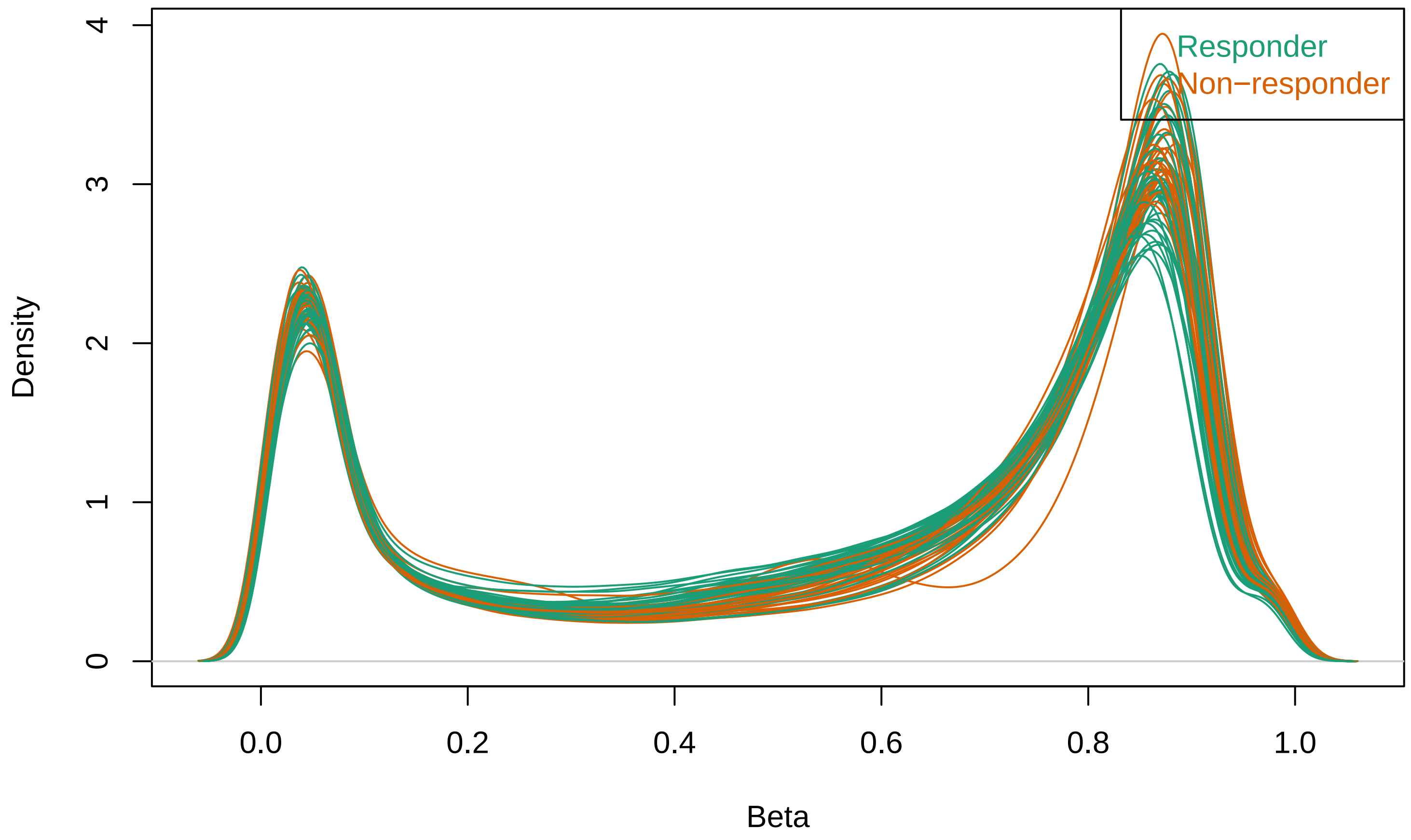

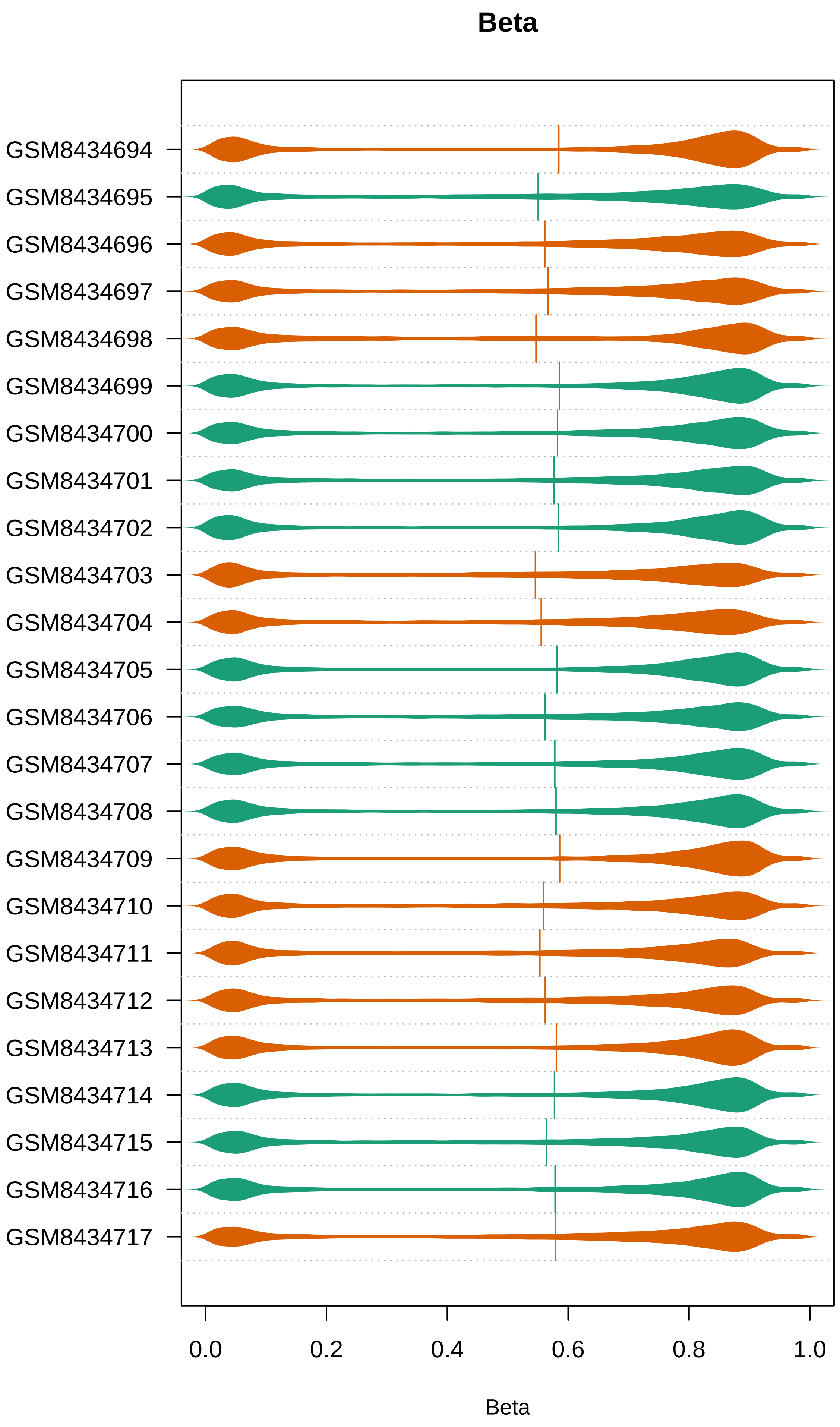

Beta
GSM8434694
GSM8434695
GSM8434696
GSM8434697
GSM8434698
GSM8434699
GSM8434700
GSM8434701
GSM8434702
GSM8434703
GSM8434704
GSM8434705
GSM8434706
GSM8434707
GSM8434708
GSM8434709
GSM8434710
GSM8434711
GSM8434712
GSM8434713
GSM8434714
GSM8434715
GSM8434716
GSM8434717
0.0
0.2
0.4
0.6
0.8
1.0
Beta

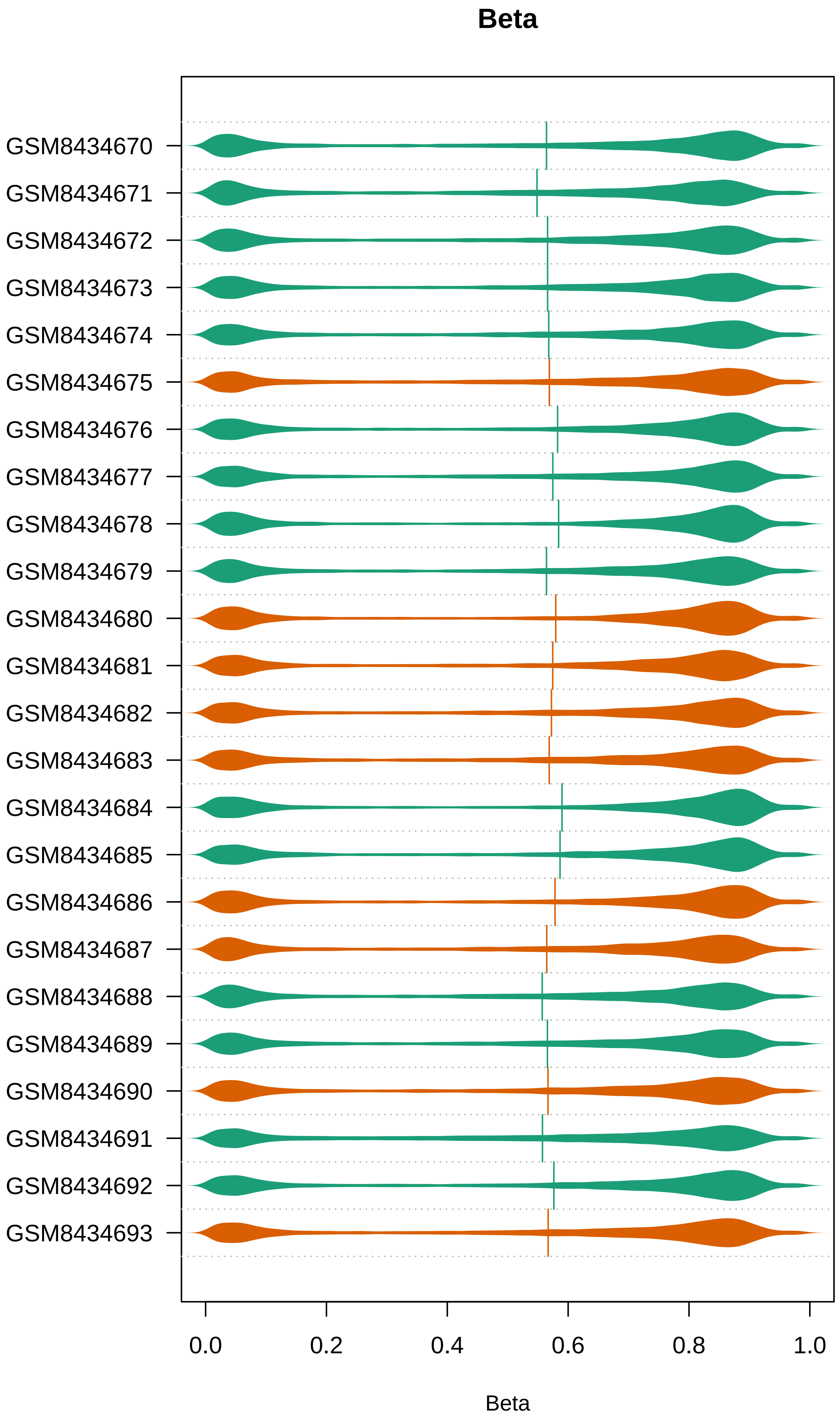
Beta
GSM8434670
GSM8434671
GSM8434672
GSM8434673
GSM8434674
GSM8434675
GSM8434676
GSM8434677
GSM8434678
GSM8434679
GSM8434680
GSM8434681
GSM8434682
GSM8434683
GSM8434684
GSM8434685
GSM8434686
GSM8434687
GSM8434688
GSM8434689
GSM8434690
GSM8434691
GSM8434692
GSM8434693
0.0
0.2
0.4
0.6
0.8
1.0
Beta

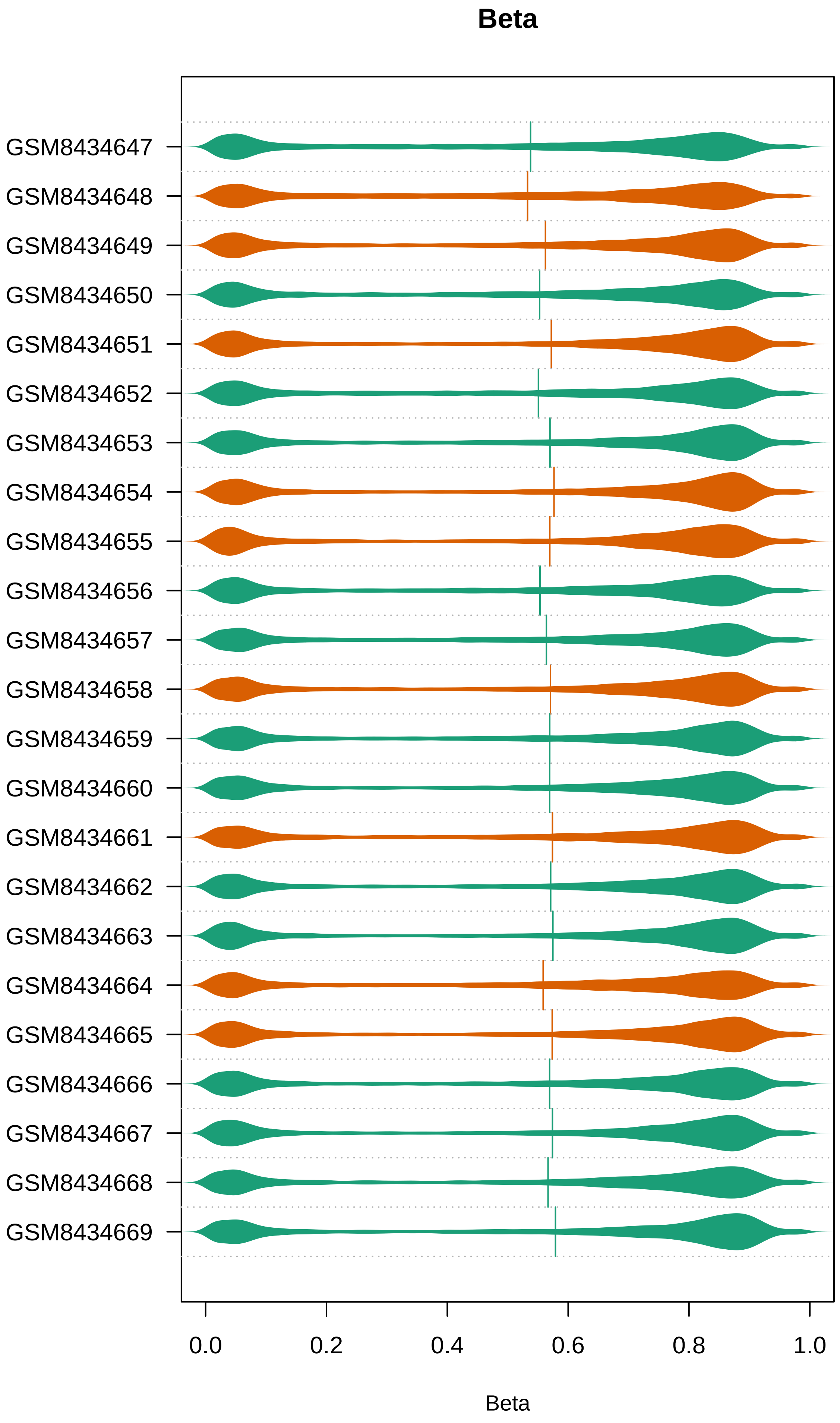

Beta
GSM8434647
GSM8434648
GSM8434649
GSM8434650
GSM8434651
GSM8434652
GSM8434653
GSM8434654
GSM8434655
GSM8434656
GSM8434657
GSM8434658
GSM8434659
GSM8434660
GSM8434661
GSM8434662
GSM8434663
GSM8434664
GSM8434665
GSM8434666
GSM8434667
GSM8434668
GSM8434669
0.0
0.2
0.4
0.6
0.8
1.0
Beta

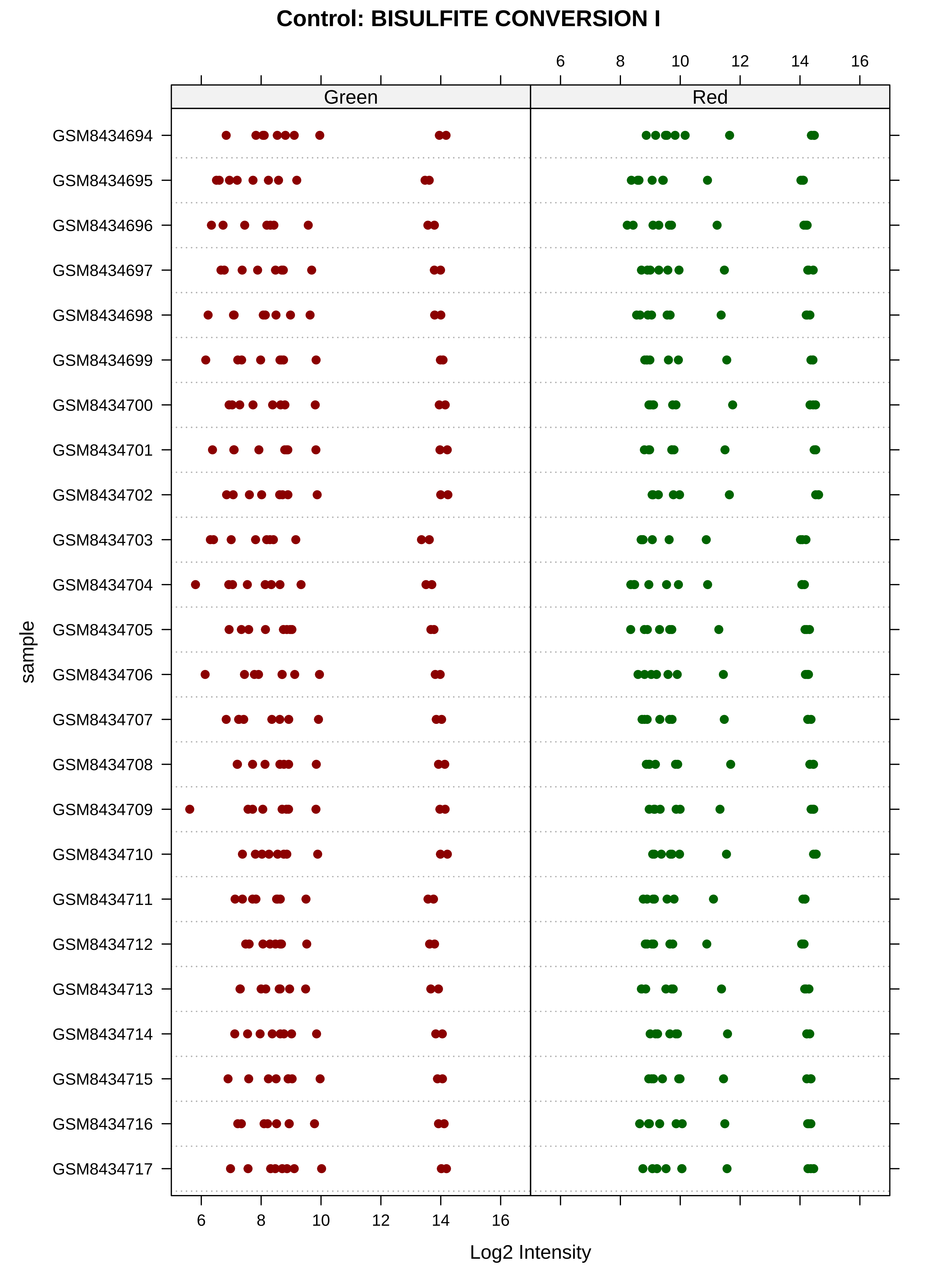

Control: BISULFITE CONVERSION I
Green
Red
sample
Log2 Intensity
GSM8434694
GSM8434695
GSM8434696
GSM8434697
GSM8434698
GSM8434699
GSM8434700
GSM8434701
GSM8434702
GSM8434703
GSM8434704
GSM8434705
GSM8434706
GSM8434707
GSM8434708
GSM8434709
GSM8434710
GSM8434711
GSM8434712
GSM8434713
GSM8434714
GSM8434715
GSM8434716
GSM8434717
6
8
10
12
14
16

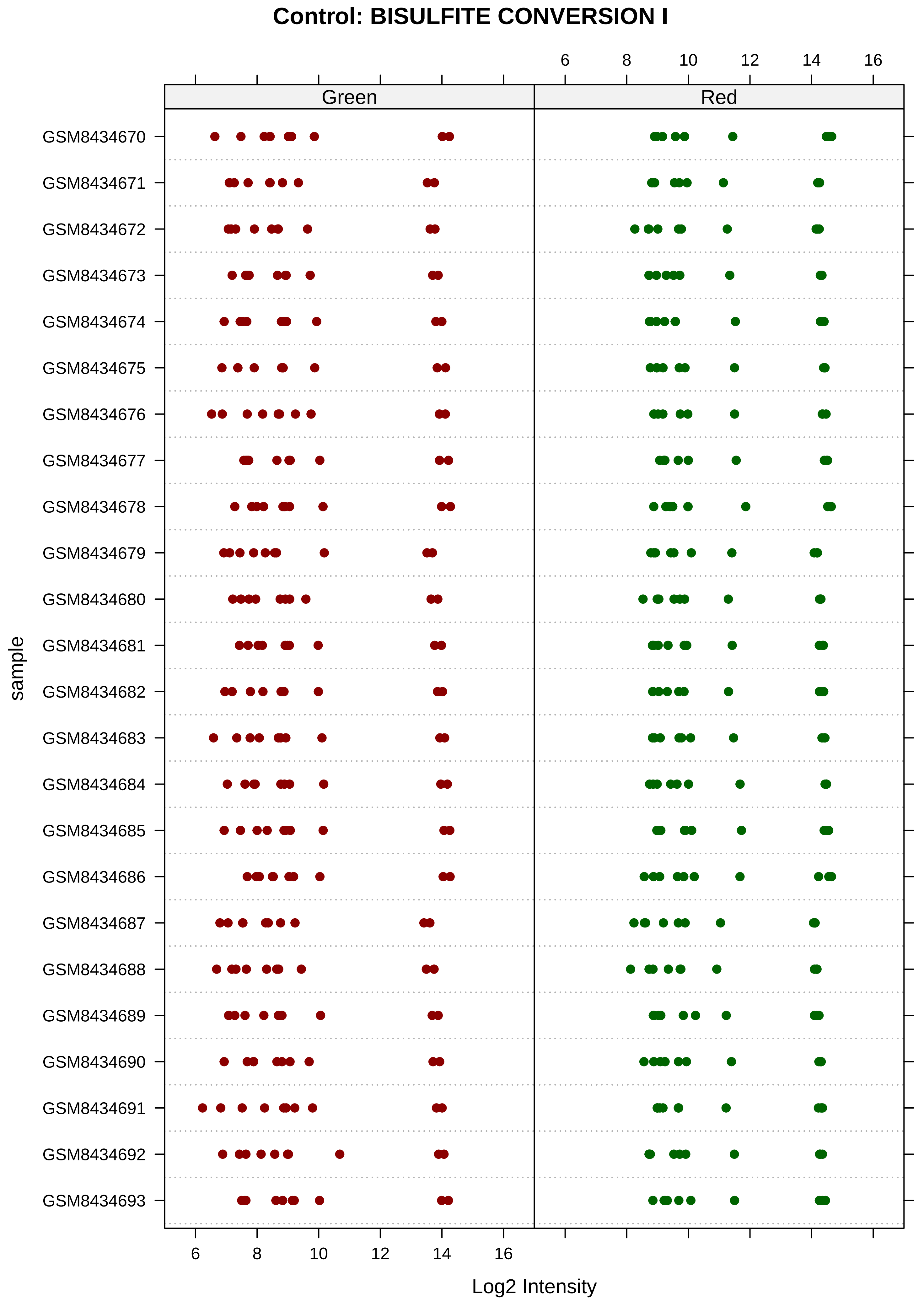

Control: BISULFITE CONVERSION I
Green
Red
GSM8434670
GSM8434671
GSM8434672
GSM8434673
GSM8434674
GSM8434675
GSM8434676
GSM8434677
GSM8434678
GSM8434679
GSM8434680
GSM8434681
GSM8434682
GSM8434683
GSM8434684
GSM8434685
GSM8434686
GSM8434687
GSM8434688
GSM8434689
GSM8434690
GSM8434691
GSM8434692
GSM8434693
sample
6 8 10 12 14 16
Log2 Intensity

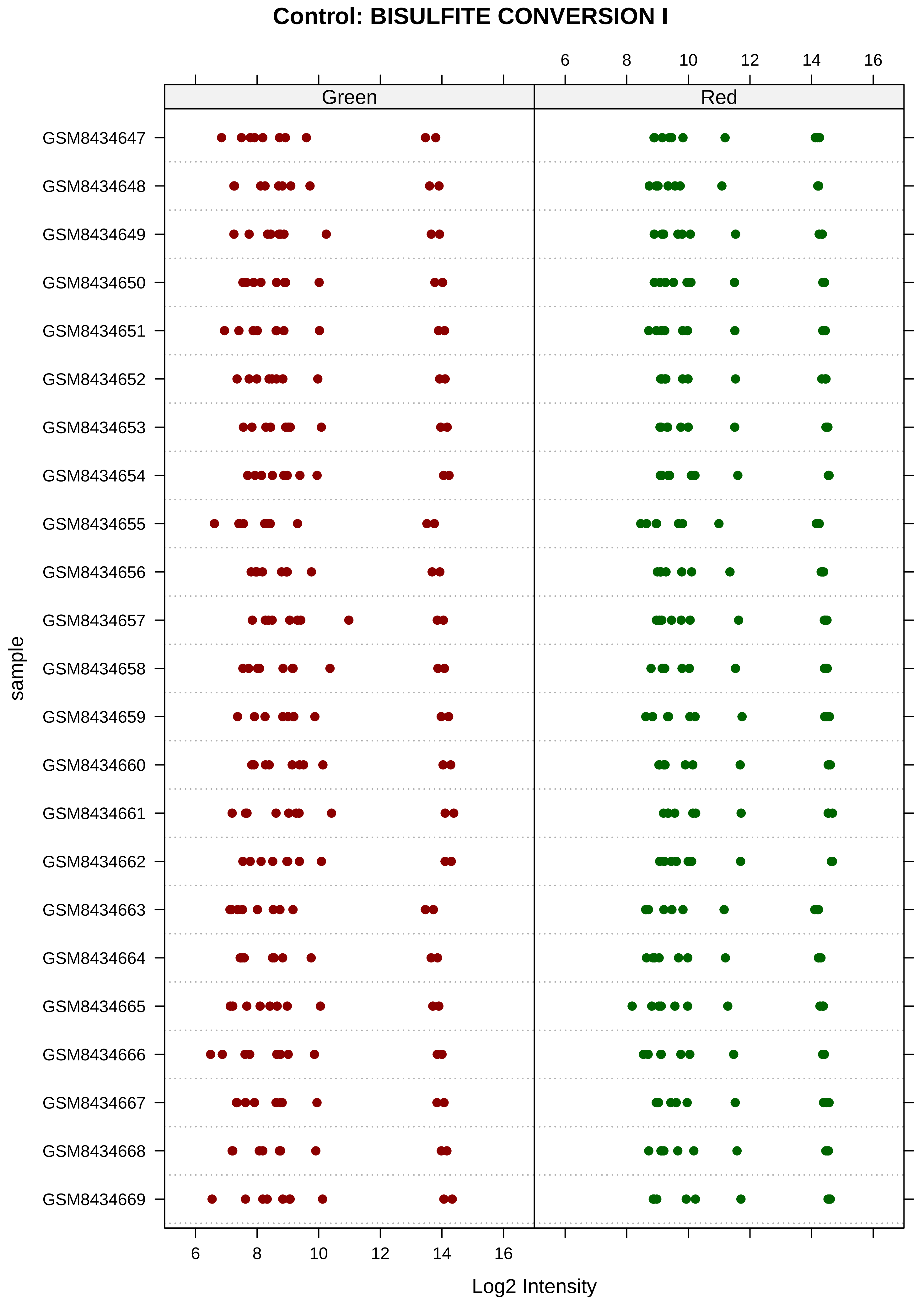

Control: BISULFITE CONVERSION I
Green
Red
sample
GSM8434647
GSM8434648
GSM8434649
GSM8434650
GSM8434651
GSM8434652
GSM8434653
GSM8434654
GSM8434655
GSM8434656
GSM8434657
GSM8434658
GSM8434659
GSM8434660
GSM8434661
GSM8434662
GSM8434663
GSM8434664
GSM8434665
GSM8434666
GSM8434667
GSM8434668
GSM8434669
6
8
10
12
14
16
Log2 Intensity

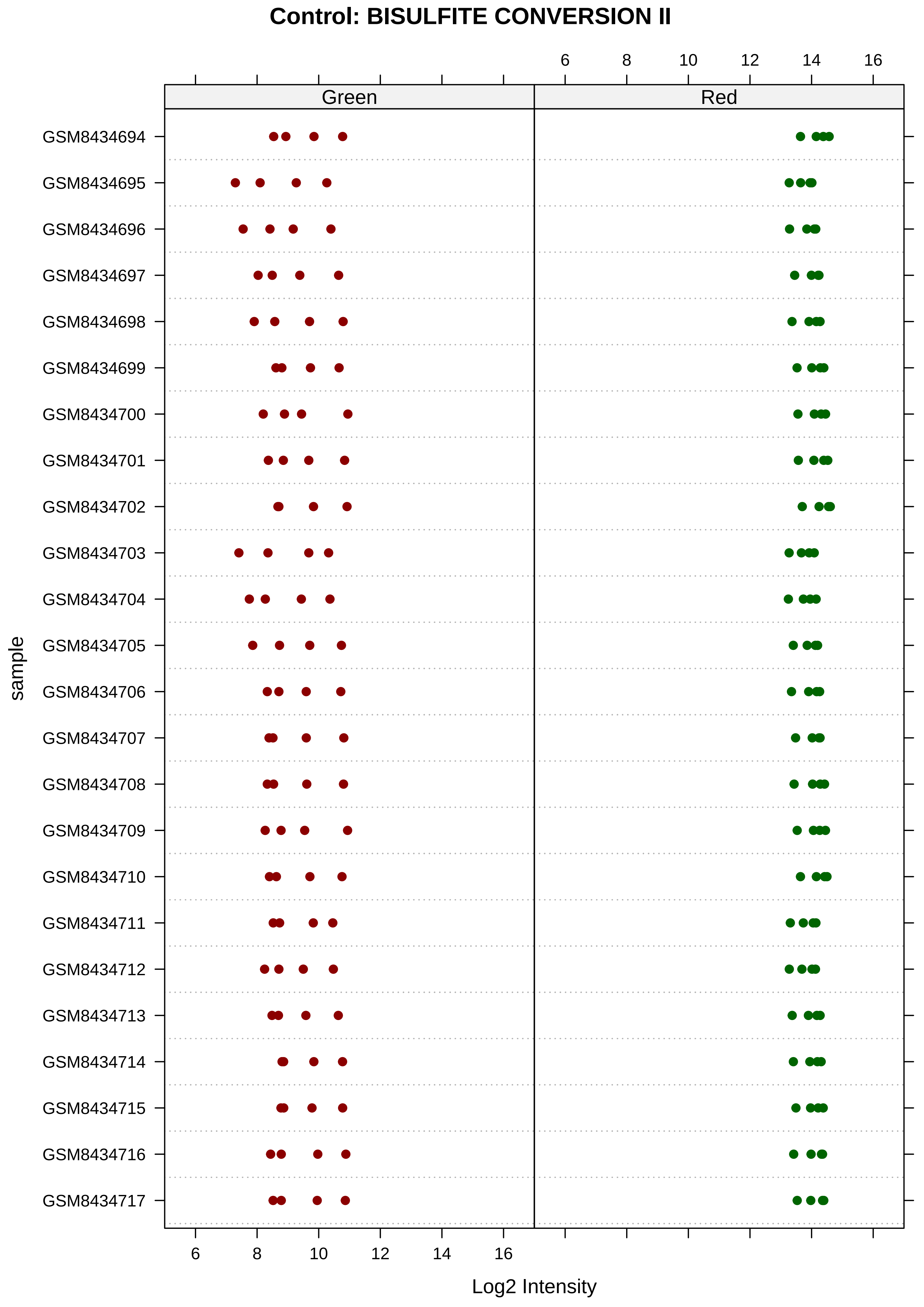

Control: BISULFITE CONVERSION II
Green
Red
sample
Log2 Intensity
GSM8434694
GSM8434695
GSM8434696
GSM8434697
GSM8434698
GSM8434699
GSM8434700
GSM8434701
GSM8434702
GSM8434703
GSM8434704
GSM8434705
GSM8434706
GSM8434707
GSM8434708
GSM8434709
GSM8434710
GSM8434711
GSM8434712
GSM8434713
GSM8434714
GSM8434715
GSM8434716
GSM8434717
6
8
10
12
14
16

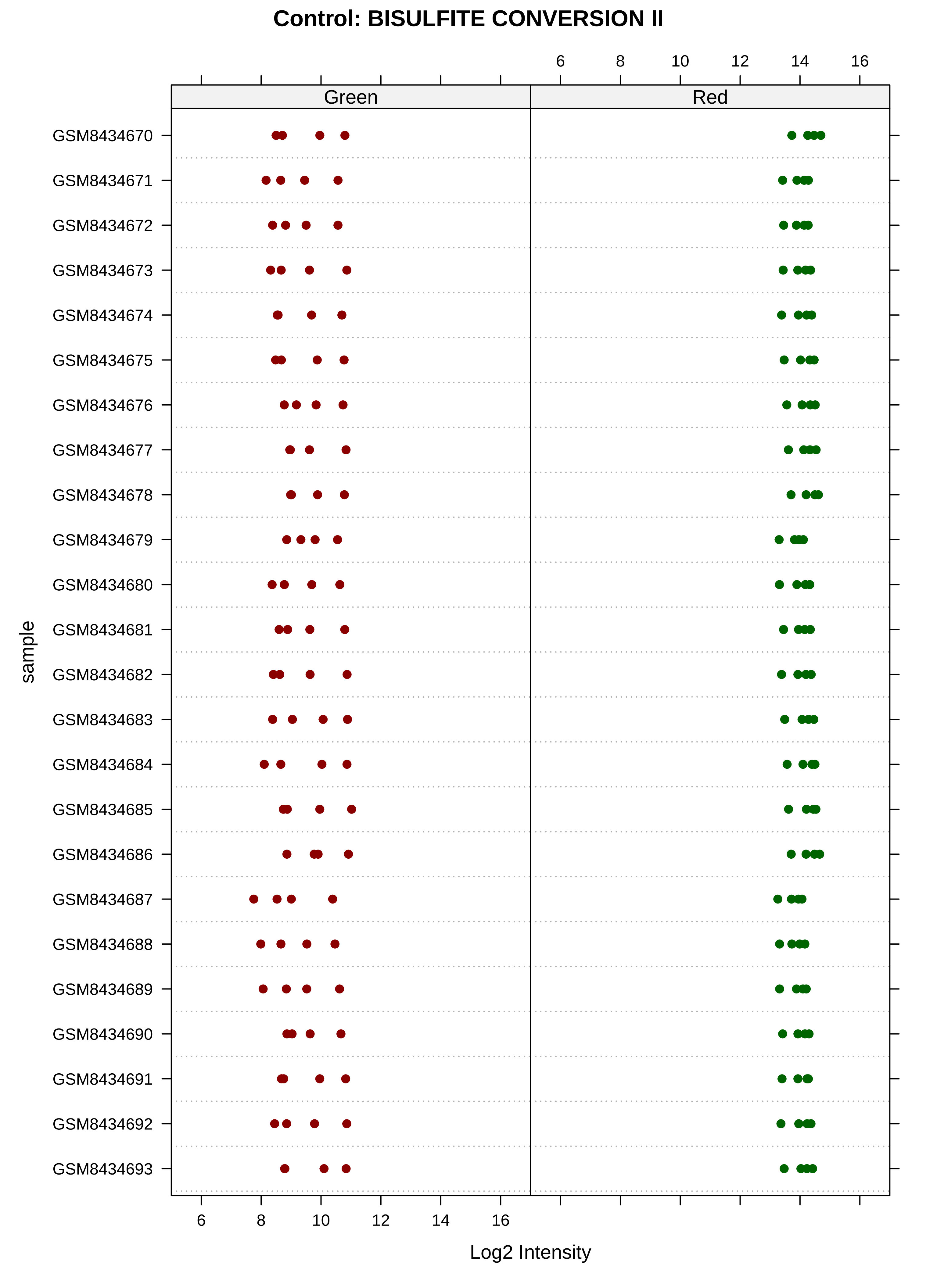

Control: BISULFITE CONVERSION II
Green
Red
sample
Log2 Intensity
GSM8434670
GSM8434671
GSM8434672
GSM8434673
GSM8434674
GSM8434675
GSM8434676
GSM8434677
GSM8434678
GSM8434679
GSM8434680
GSM8434681
GSM8434682
GSM8434683
GSM8434684
GSM8434685
GSM8434686
GSM8434687
GSM8434688
GSM8434689
GSM8434690
GSM8434691
GSM8434692
GSM8434693
6
8
10
12
14
16

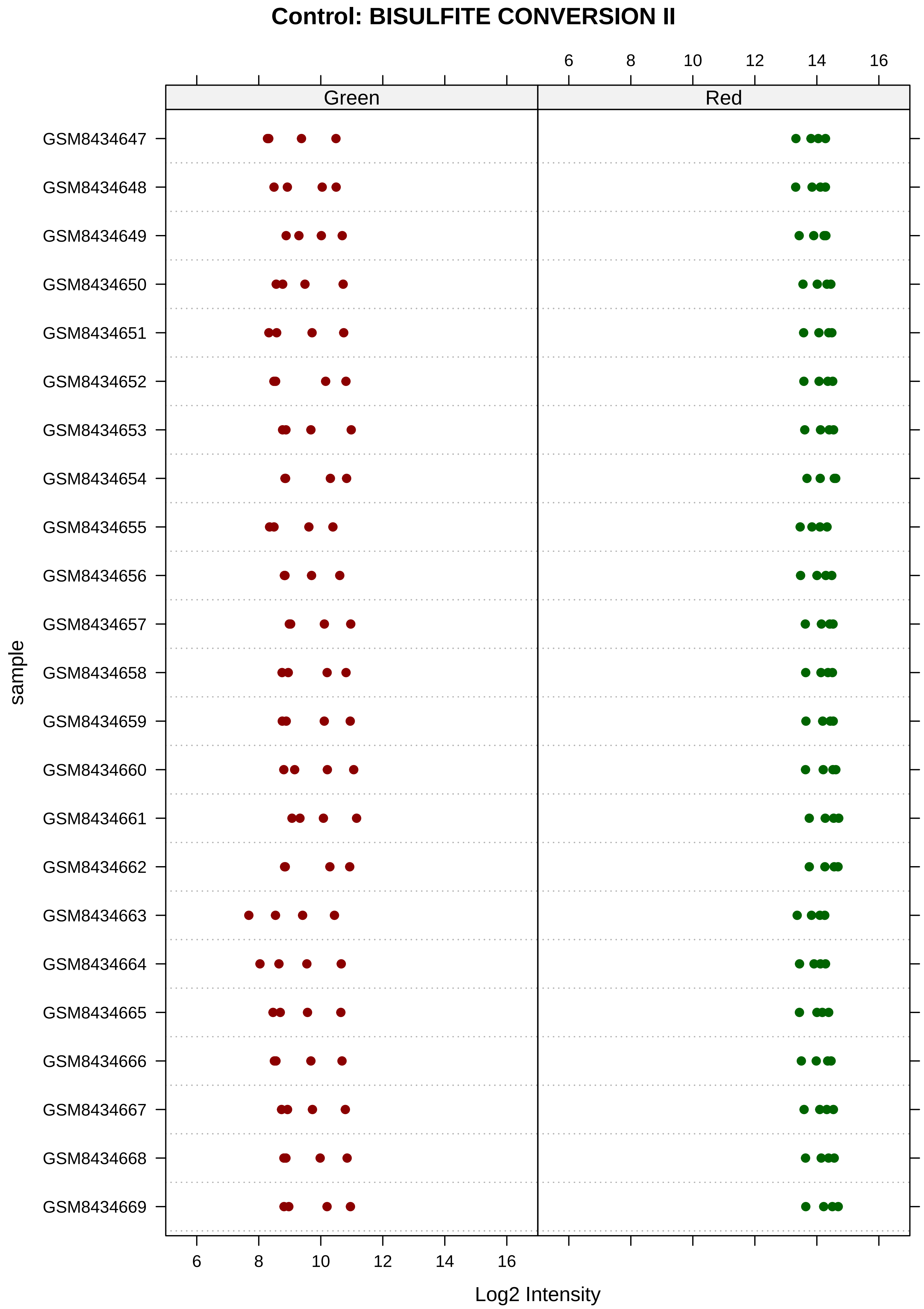

Control: BISULFITE CONVERSION II
Green
Red
sample
Log2 Intensity
GSM8434647
GSM8434648
GSM8434649
GSM8434650
GSM8434651
GSM8434652
GSM8434653
GSM8434654
GSM8434655
GSM8434656
GSM8434657
GSM8434658
GSM8434659
GSM8434660
GSM8434661
GSM8434662
GSM8434663
GSM8434664
GSM8434665
GSM8434666
GSM8434667
GSM8434668
GSM8434669
6
8
10
12
14
16

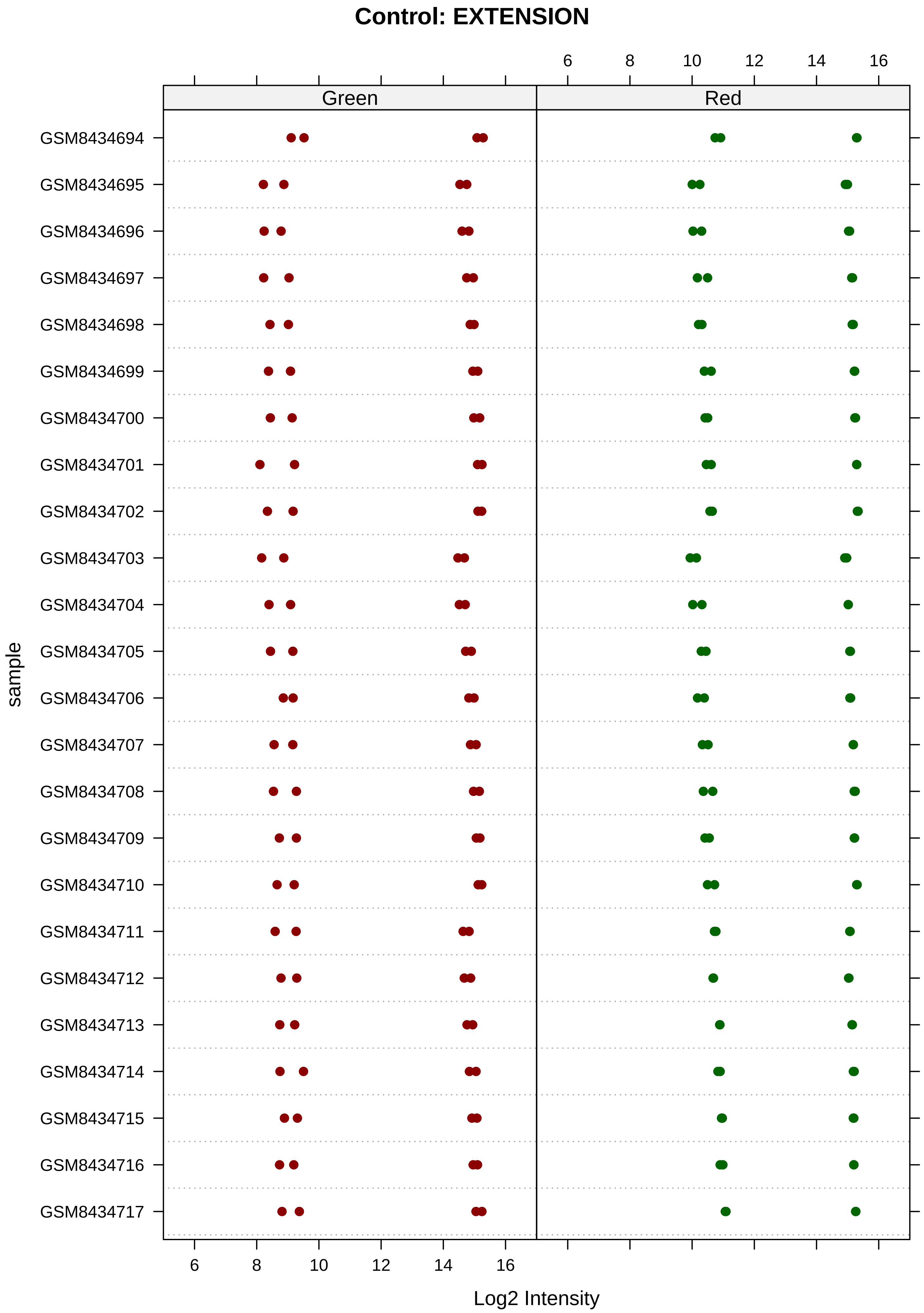

Control: EXTENSION
Green
Red
sample
GSM8434694
GSM8434695
GSM8434696
GSM8434697
GSM8434698
GSM8434699
GSM8434700
GSM8434701
GSM8434702
GSM8434703
GSM8434704
GSM8434705
GSM8434706
GSM8434707
GSM8434708
GSM8434709
GSM8434710
GSM8434711
GSM8434712
GSM8434713
GSM8434714
GSM8434715
GSM8434716
GSM8434717
6
8
10
12
14
16
Log2 Intensity

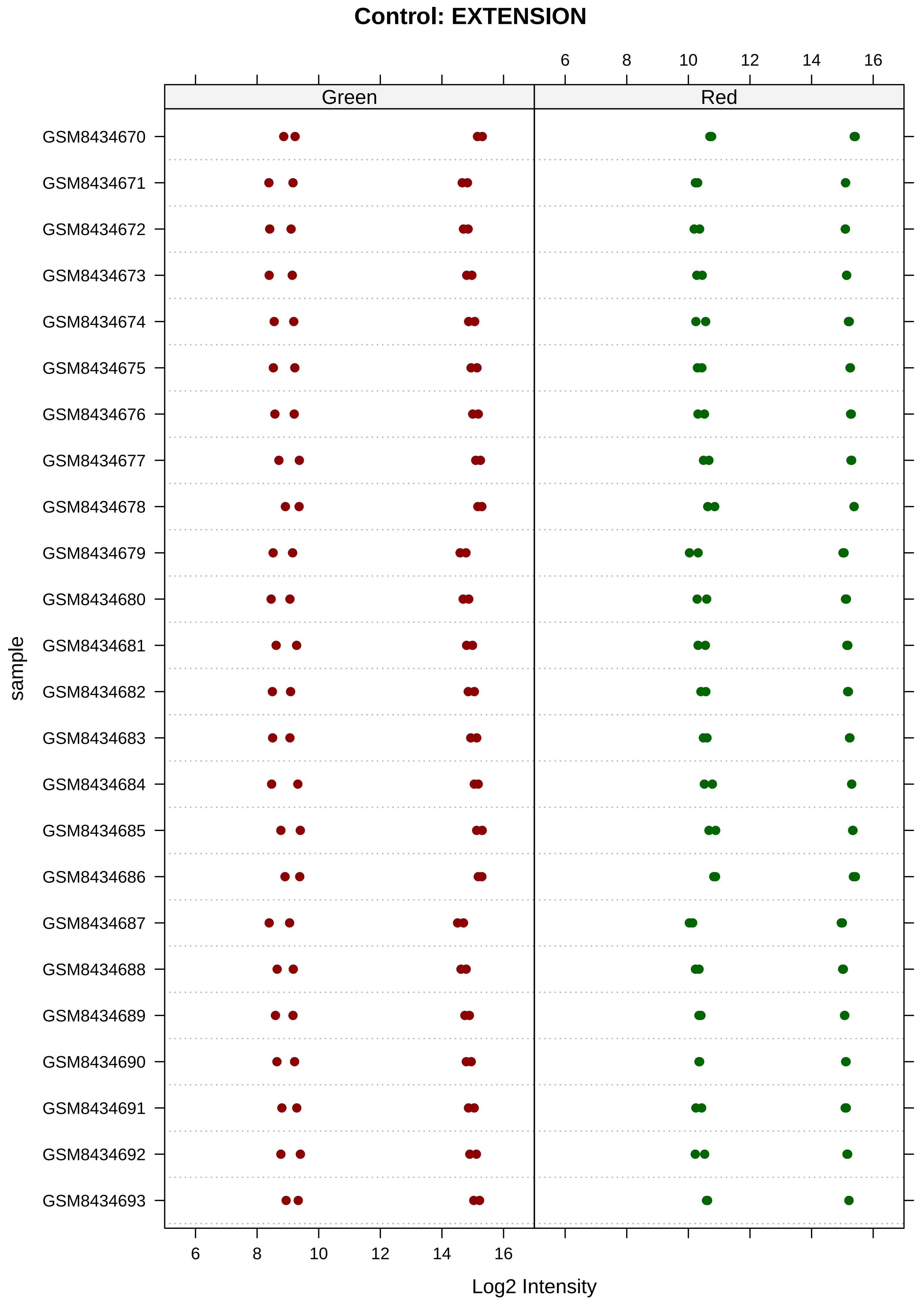
Control: EXTENSION
Green
Red
sample
GSM8434670
GSM8434671
GSM8434672
GSM8434673
GSM8434674
GSM8434675
GSM8434676
GSM8434677
GSM8434678
GSM8434679
GSM8434680
GSM8434681
GSM8434682
GSM8434683
GSM8434684
GSM8434685
GSM8434686
GSM8434687
GSM8434688
GSM8434689
GSM8434690
GSM8434691
GSM8434692
GSM8434693
6
8
10
12
14
16
Log2 Intensity

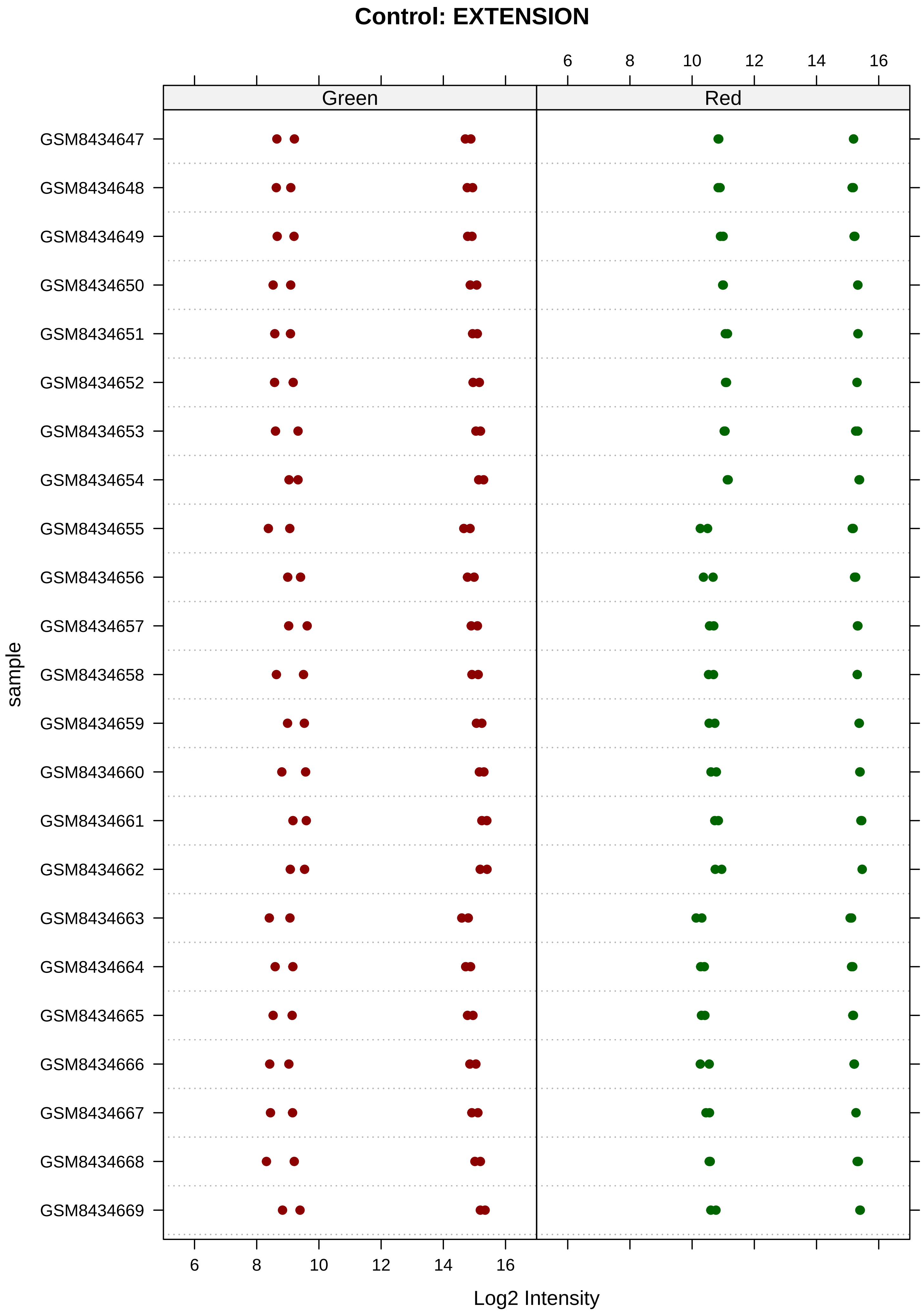
Control: EXTENSION
Green
Red
sample
Log2 Intensity
6
8
10
12
14
16
GSM8434647
GSM8434648
GSM8434649
GSM8434650
GSM8434651
GSM8434652
GSM8434653
GSM8434654
GSM8434655
GSM8434656
GSM8434657
GSM8434658
GSM8434659
GSM8434660
GSM8434661
GSM8434662
GSM8434663
GSM8434664
GSM8434665
GSM8434666
GSM8434667
GSM8434668
GSM8434669

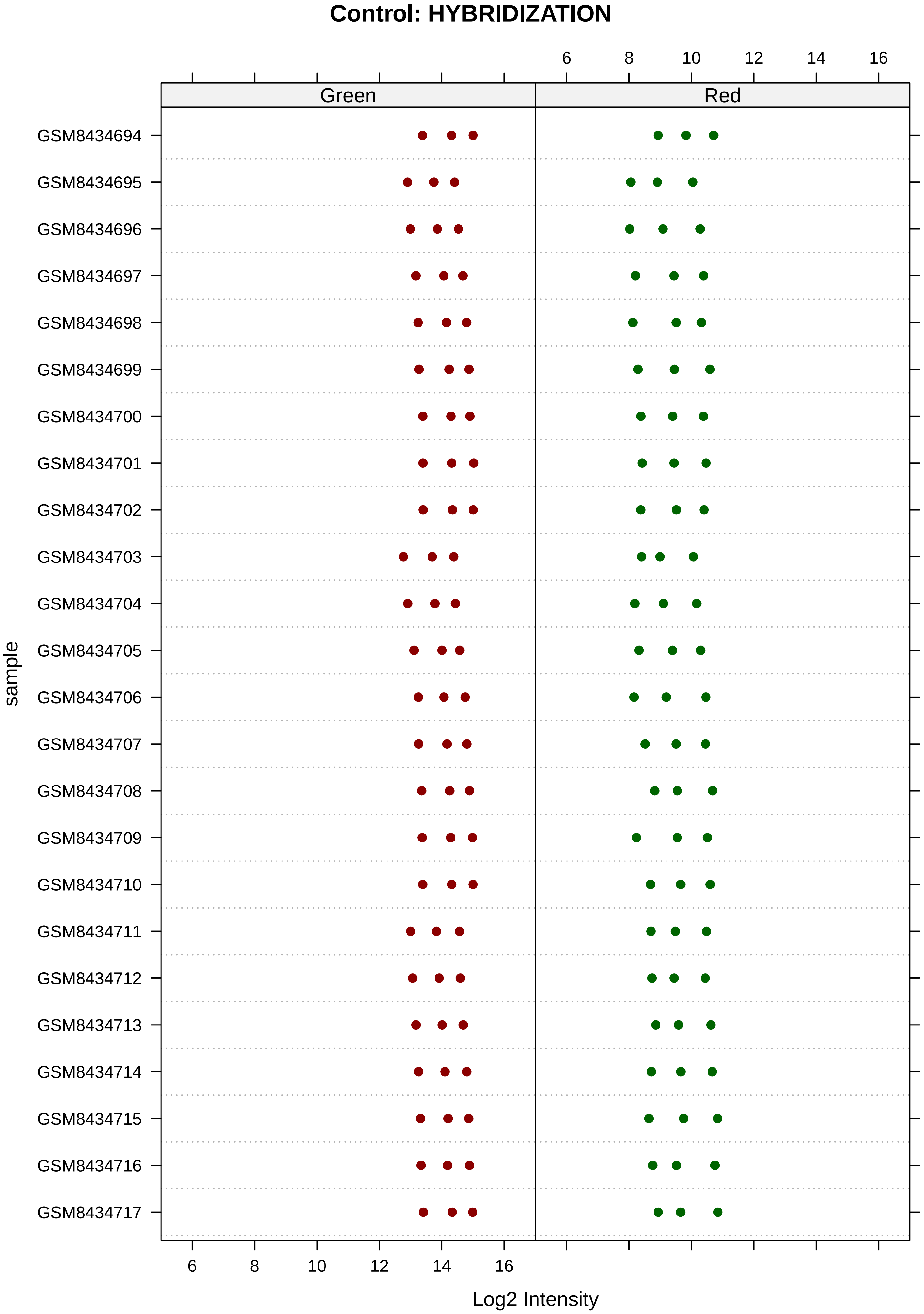
Control: HYBRIDIZATION
Green
Red
sample
Log2 Intensity
6
8
10
12
14
16
GSM8434694
GSM8434695
GSM8434696
GSM8434697
GSM8434698
GSM8434699
GSM8434700
GSM8434701
GSM8434702
GSM8434703
GSM8434704
GSM8434705
GSM8434706
GSM8434707
GSM8434708
GSM8434709
GSM8434710
GSM8434711
GSM8434712
GSM8434713
GSM8434714
GSM8434715
GSM8434716
GSM8434717

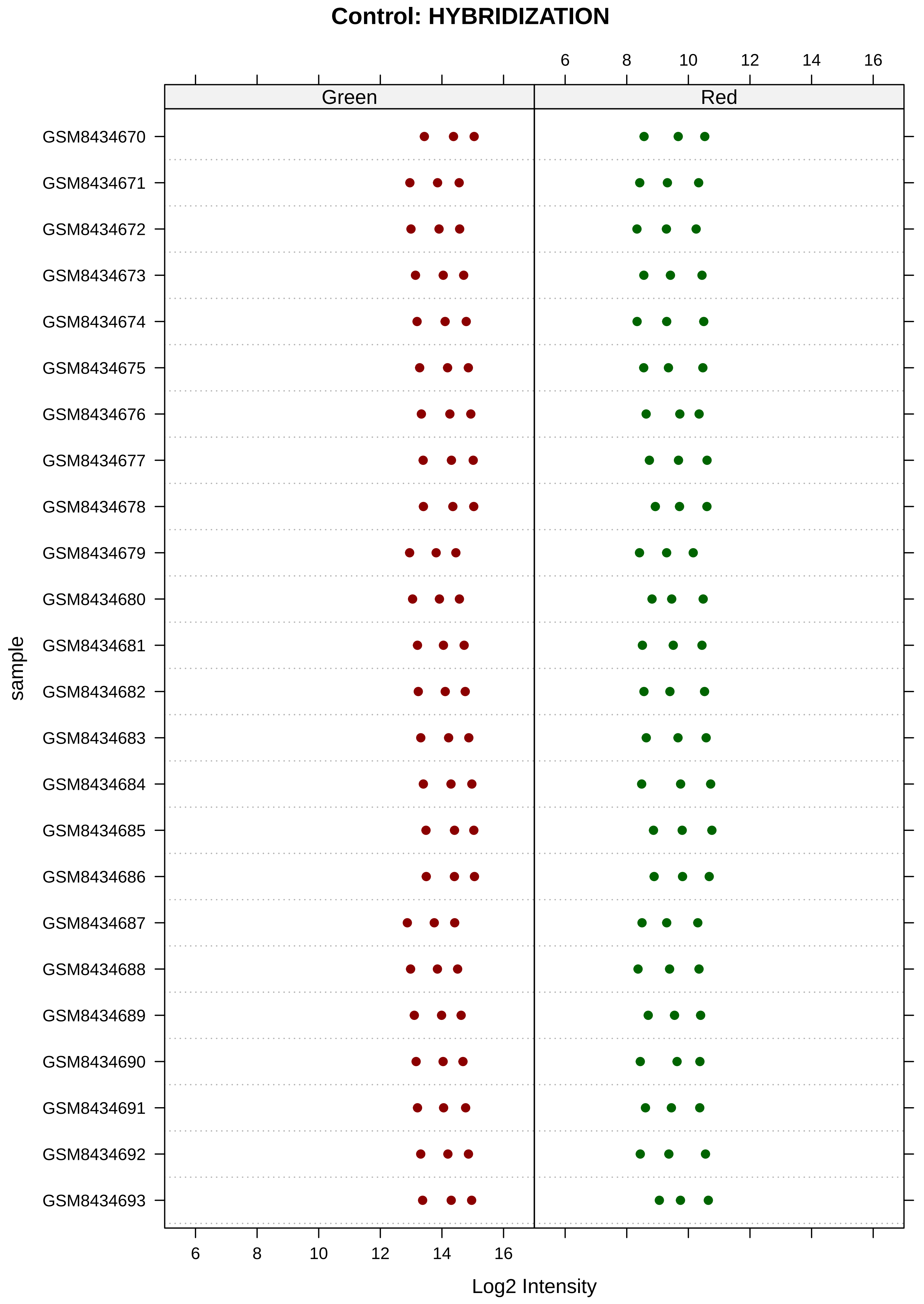

Control: HYBRIDIZATION
Green
Red
sample
Log2 Intensity
GSM8434670
GSM8434671
GSM8434672
GSM8434673
GSM8434674
GSM8434675
GSM8434676
GSM8434677
GSM8434678
GSM8434679
GSM8434680
GSM8434681
GSM8434682
GSM8434683
GSM8434684
GSM8434685
GSM8434686
GSM8434687
GSM8434688
GSM8434689
GSM8434690
GSM8434691
GSM8434692
GSM8434693
6
8
10
12
14
16

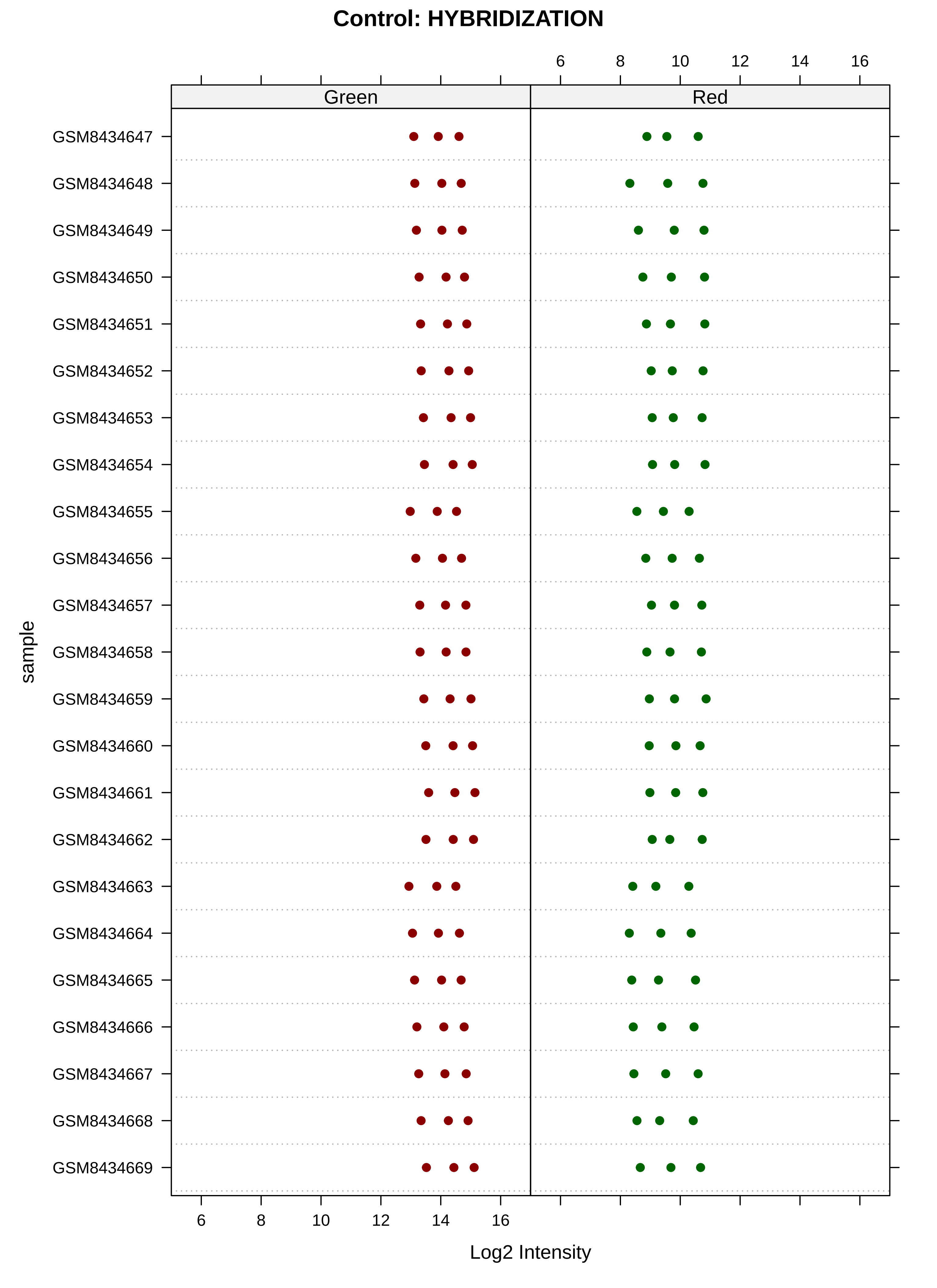

Control: HYBRIDIZATION
Green
Red
sample
Log2 Intensity
GSM8434647
GSM8434648
GSM8434649
GSM8434650
GSM8434651
GSM8434652
GSM8434653
GSM8434654
GSM8434655
GSM8434656
GSM8434657
GSM8434658
GSM8434659
GSM8434660
GSM8434661
GSM8434662
GSM8434663
GSM8434664
GSM8434665
GSM8434666
GSM8434667
GSM8434668
GSM8434669
6
8
10
12
14
16

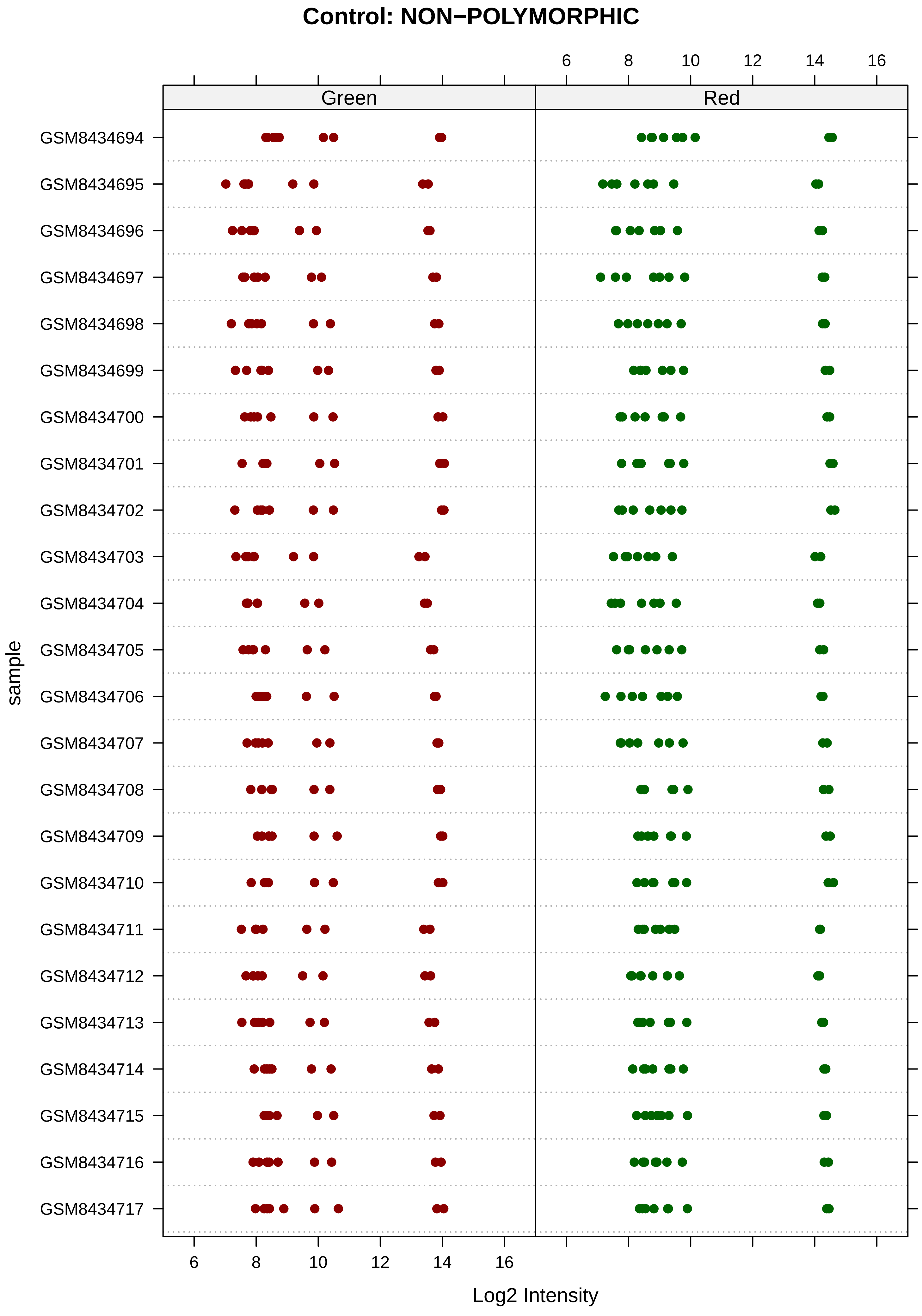

Control: NON−POLYMORPHIC
Green
Red
sample
Log2 Intensity
GSM8434694
GSM8434695
GSM8434696
GSM8434697
GSM8434698
GSM8434699
GSM8434700
GSM8434701
GSM8434702
GSM8434703
GSM8434704
GSM8434705
GSM8434706
GSM8434707
GSM8434708
GSM8434709
GSM8434710
GSM8434711
GSM8434712
GSM8434713
GSM8434714
GSM8434715
GSM8434716
GSM8434717
6
8
10
12
14
16

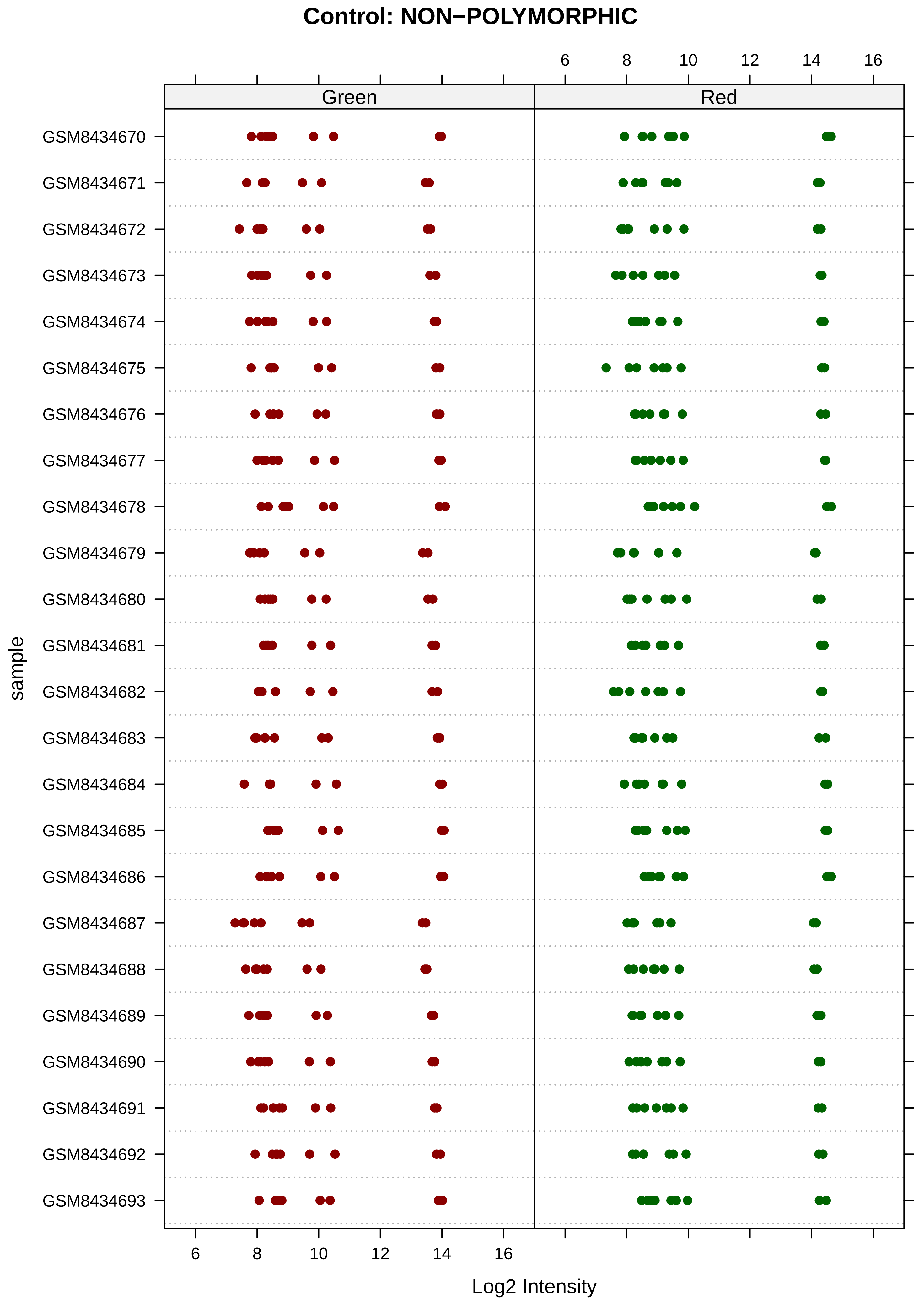

Control: NON−POLYMORPHIC
Green
Red
sample
GSM8434670
GSM8434671
GSM8434672
GSM8434673
GSM8434674
GSM8434675
GSM8434676
GSM8434677
GSM8434678
GSM8434679
GSM8434680
GSM8434681
GSM8434682
GSM8434683
GSM8434684
GSM8434685
GSM8434686
GSM8434687
GSM8434688
GSM8434689
GSM8434690
GSM8434691
GSM8434692
GSM8434693
6
8
10
12
14
16
Log2 Intensity

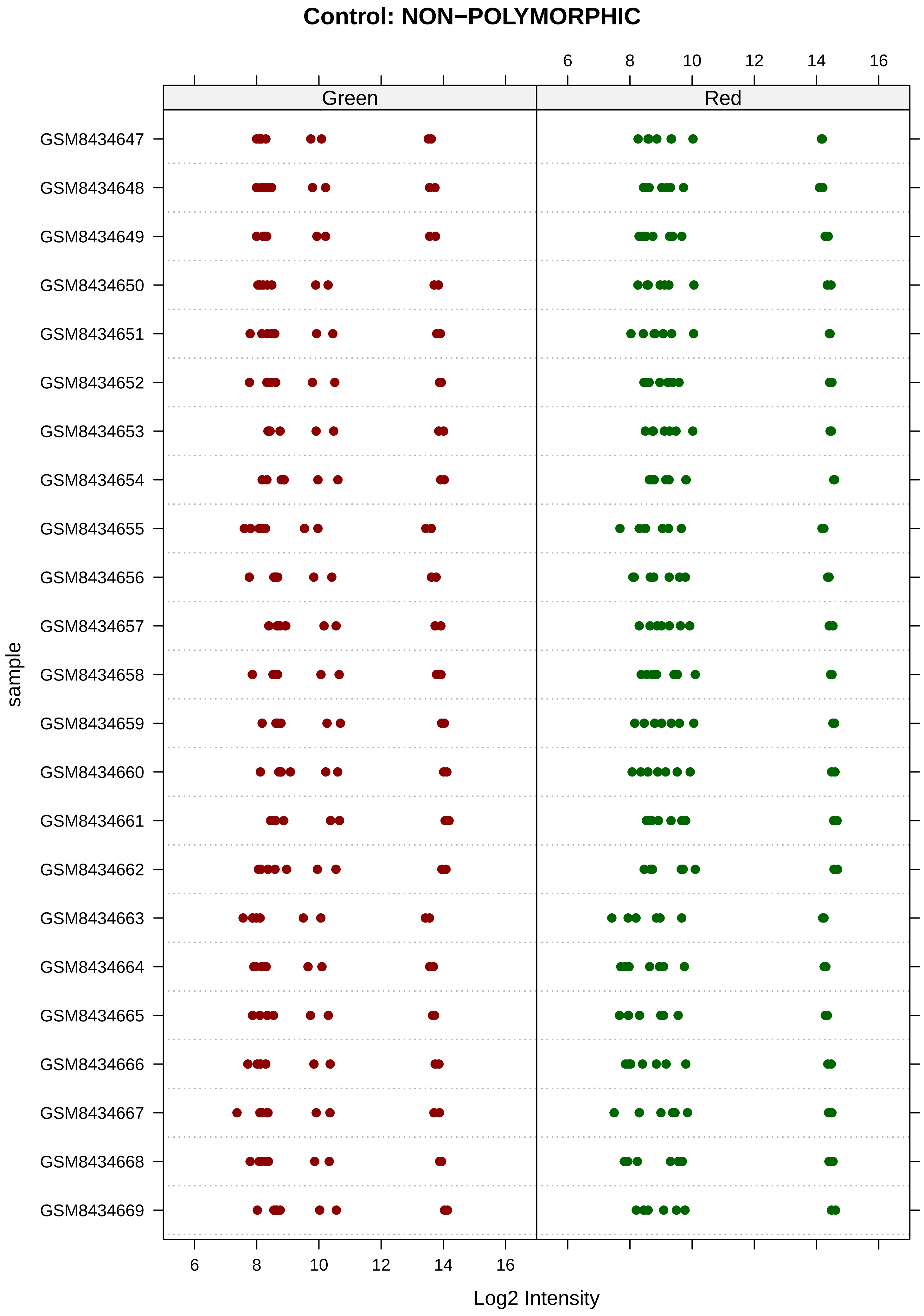

Control: NON−POLYMORPHIC
Green
Red
sample
GSM8434647
GSM8434648
GSM8434649
GSM8434650
GSM8434651
GSM8434652
GSM8434653
GSM8434654
GSM8434655
GSM8434656
GSM8434657
GSM8434658
GSM8434659
GSM8434660
GSM8434661
GSM8434662
GSM8434663
GSM8434664
GSM8434665
GSM8434666
GSM8434667
GSM8434668
GSM8434669
6 8 10 12 14 16
Log2 Intensity

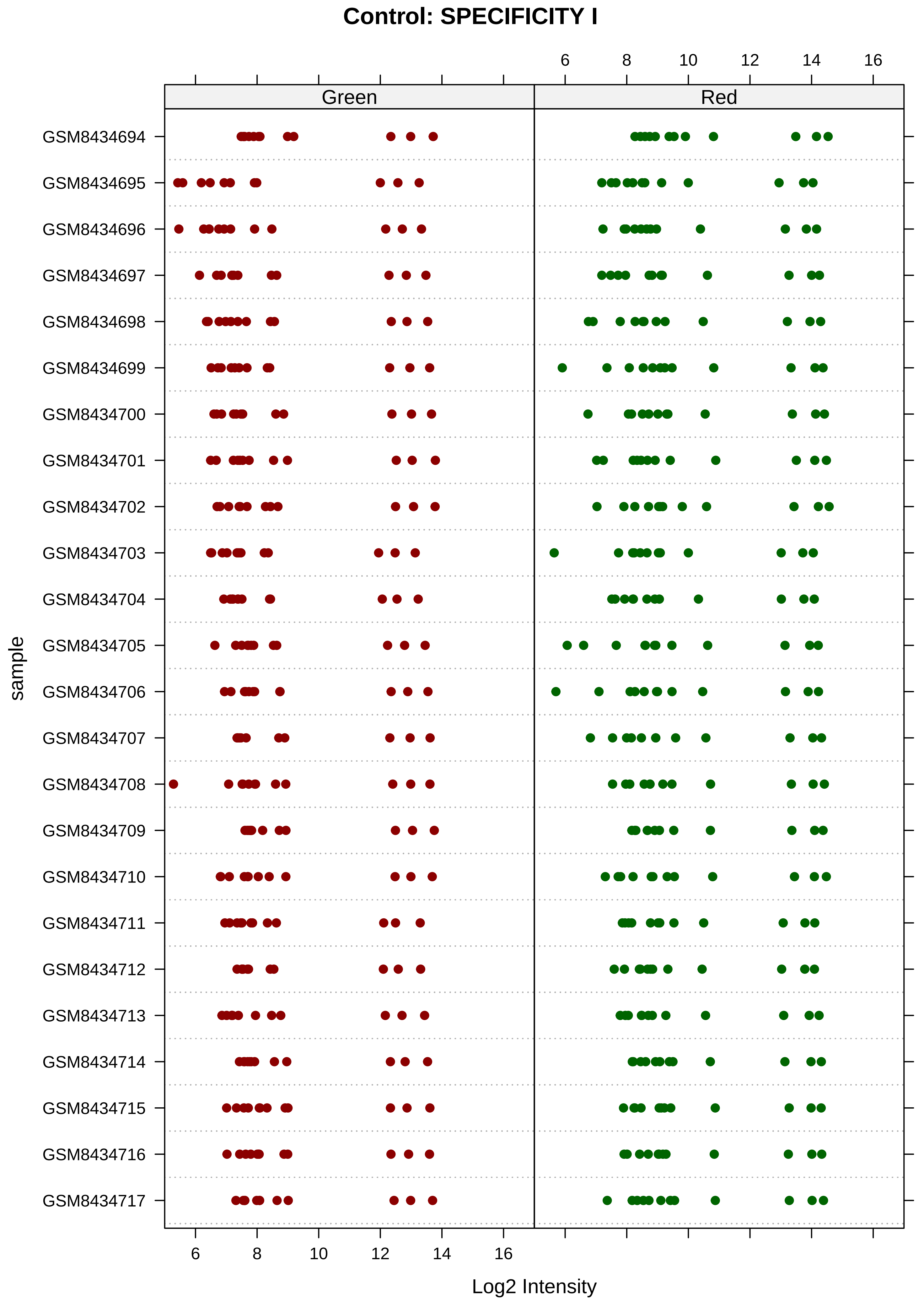

Control: SPECIFICITY I
Green
Red
sample
Log2 Intensity
GSM8434694
GSM8434695
GSM8434696
GSM8434697
GSM8434698
GSM8434699
GSM8434700
GSM8434701
GSM8434702
GSM8434703
GSM8434704
GSM8434705
GSM8434706
GSM8434707
GSM8434708
GSM8434709
GSM8434710
GSM8434711
GSM8434712
GSM8434713
GSM8434714
GSM8434715
GSM8434716
GSM8434717
6
8
10
12
14
16

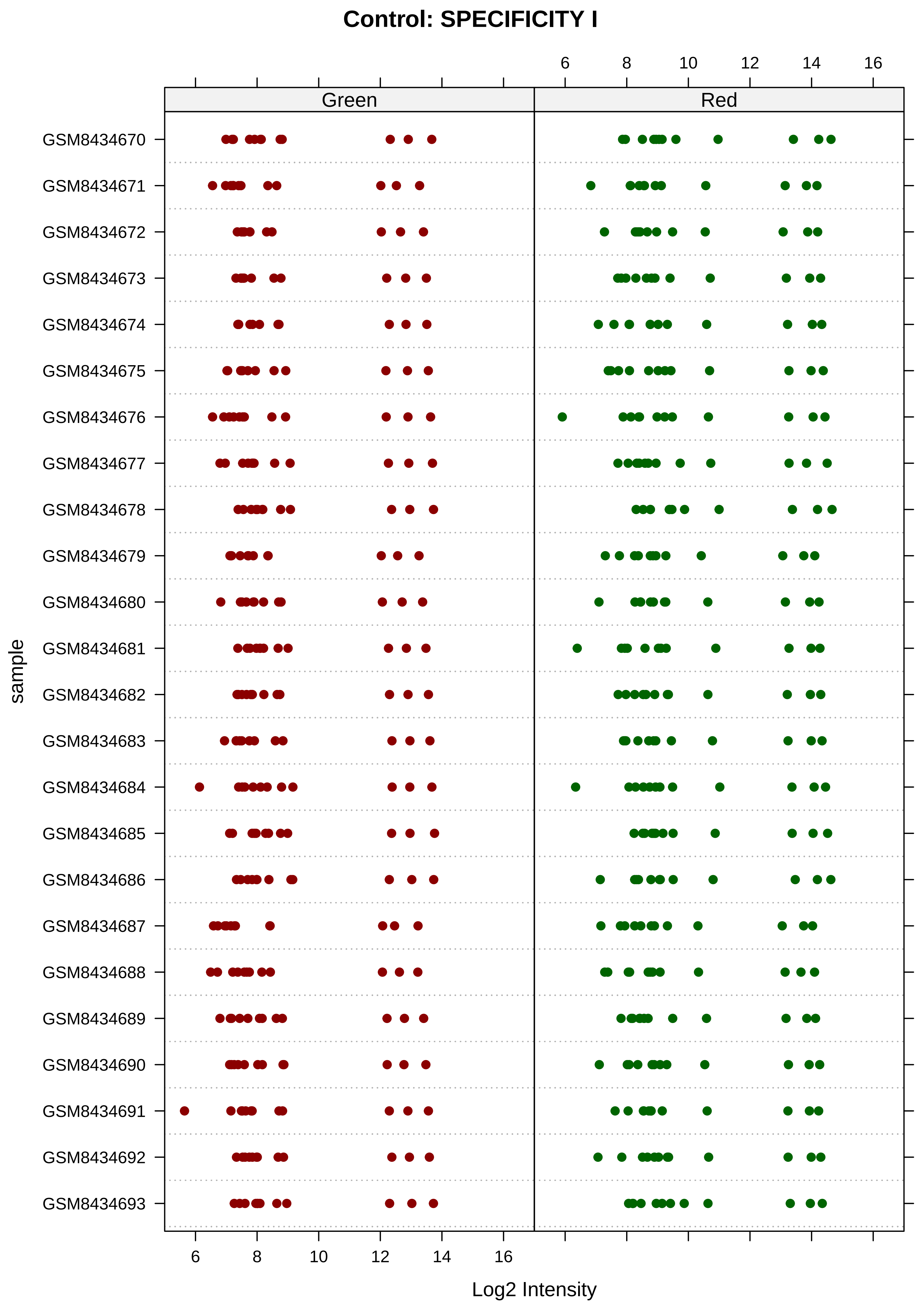

Control: SPECIFICITY I
Green
Red
sample
Log2 Intensity
GSM8434670
GSM8434671
GSM8434672
GSM8434673
GSM8434674
GSM8434675
GSM8434676
GSM8434677
GSM8434678
GSM8434679
GSM8434680
GSM8434681
GSM8434682
GSM8434683
GSM8434684
GSM8434685
GSM8434686
GSM8434687
GSM8434688
GSM8434689
GSM8434690
GSM8434691
GSM8434692
GSM8434693
6 8 10 12 14 16

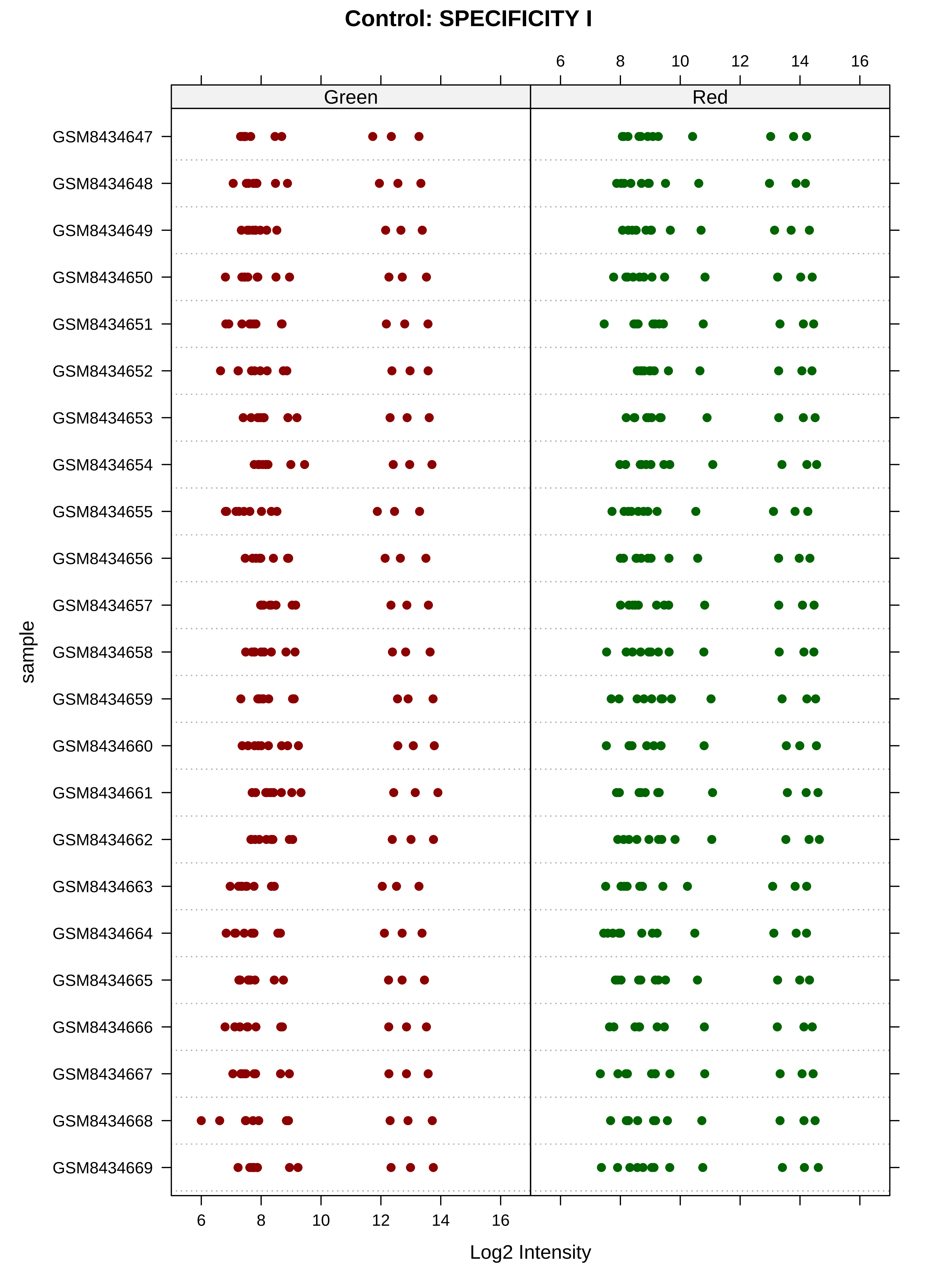
Control: SPECIFICITY I
Green
Red
sample
GSM8434647
GSM8434648
GSM8434649
GSM8434650
GSM8434651
GSM8434652
GSM8434653
GSM8434654
GSM8434655
GSM8434656
GSM8434657
GSM8434658
GSM8434659
GSM8434660
GSM8434661
GSM8434662
GSM8434663
GSM8434664
GSM8434665
GSM8434666
GSM8434667
GSM8434668
GSM8434669
6
8
10
12
14
16
Log2 Intensity

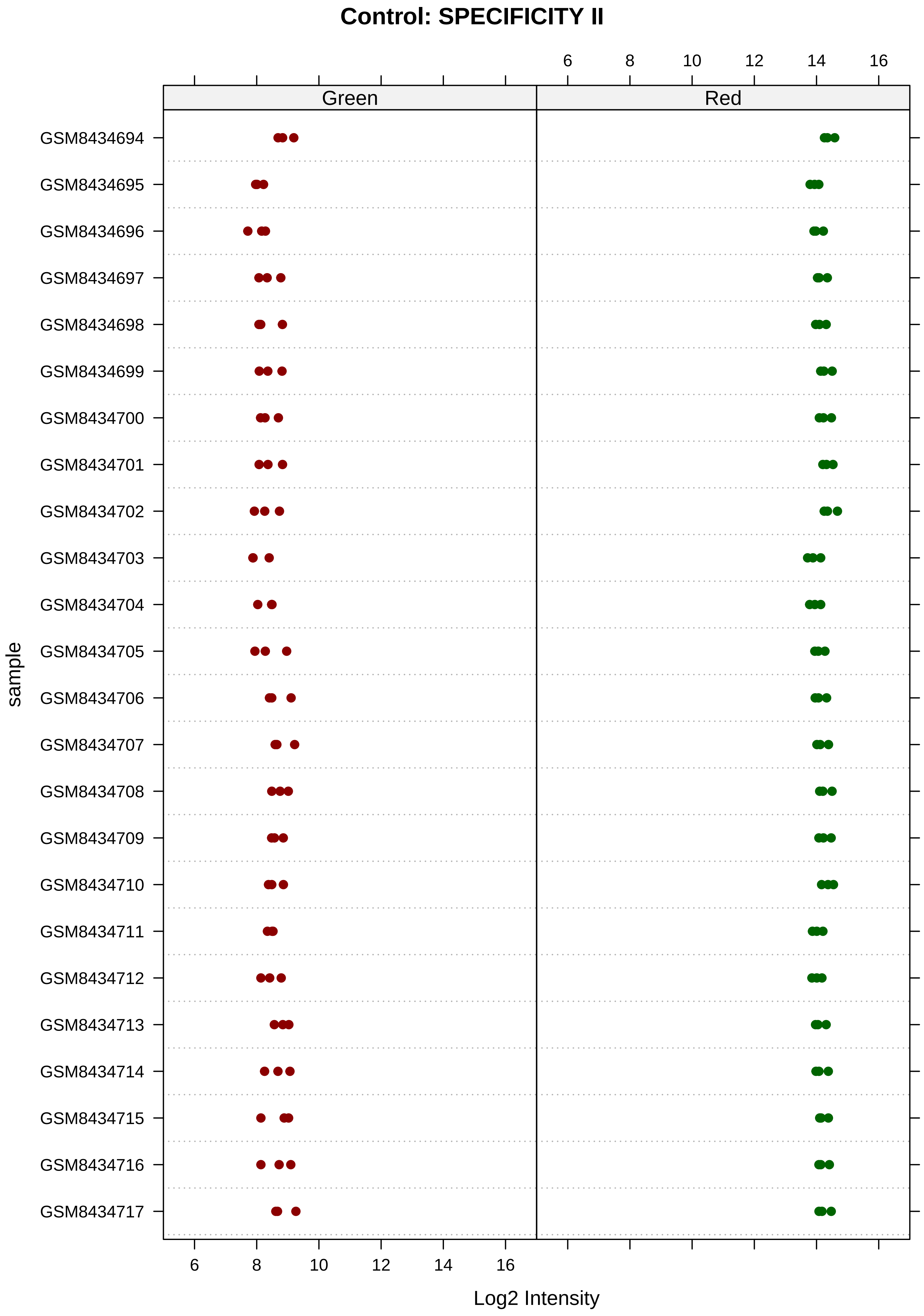
Control: SPECIFICITY II
Green
Red
sample
Log2 Intensity
6
8
10
12
14
16
GSM8434694
GSM8434695
GSM8434696
GSM8434697
GSM8434698
GSM8434699
GSM8434700
GSM8434701
GSM8434702
GSM8434703
GSM8434704
GSM8434705
GSM8434706
GSM8434707
GSM8434708
GSM8434709
GSM8434710
GSM8434711
GSM8434712
GSM8434713
GSM8434714
GSM8434715
GSM8434716
GSM8434717

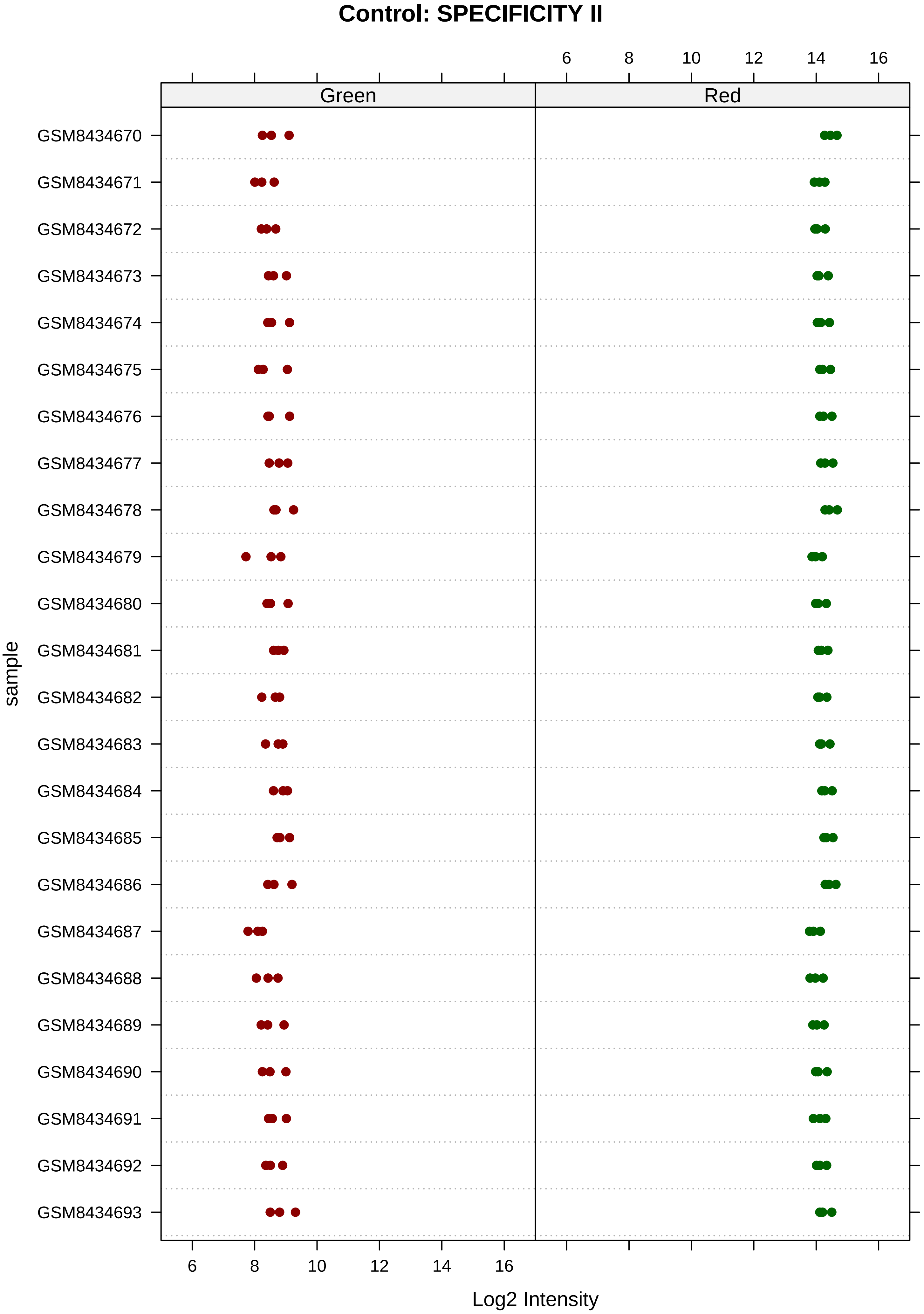

Control: SPECIFICITY II
Green
Red
sample
Log2 Intensity
GSM8434670
GSM8434671
GSM8434672
GSM8434673
GSM8434674
GSM8434675
GSM8434676
GSM8434677
GSM8434678
GSM8434679
GSM8434680
GSM8434681
GSM8434682
GSM8434683
GSM8434684
GSM8434685
GSM8434686
GSM8434687
GSM8434688
GSM8434689
GSM8434690
GSM8434691
GSM8434692
GSM8434693
6
8
10
12
14
16

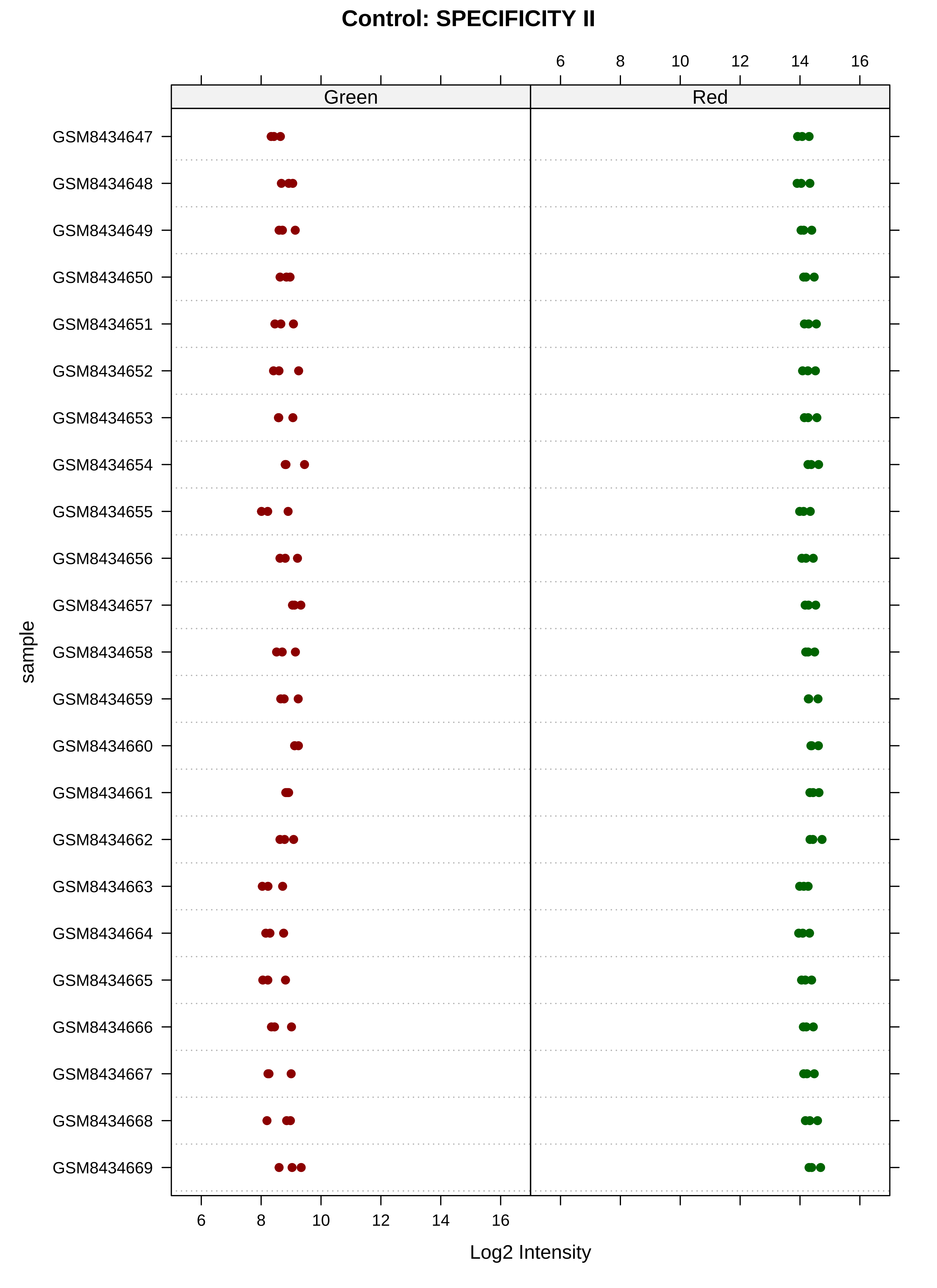
Control: SPECIFICITY II
Green
Red
sample
Log2 Intensity
6
8
10
12
14
16
GSM8434647
GSM8434648
GSM8434649
GSM8434650
GSM8434651
GSM8434652
GSM8434653
GSM8434654
GSM8434655
GSM8434656
GSM8434657
GSM8434658
GSM8434659
GSM8434660
GSM8434661
GSM8434662
GSM8434663
GSM8434664
GSM8434665
GSM8434666
GSM8434667
GSM8434668
GSM8434669

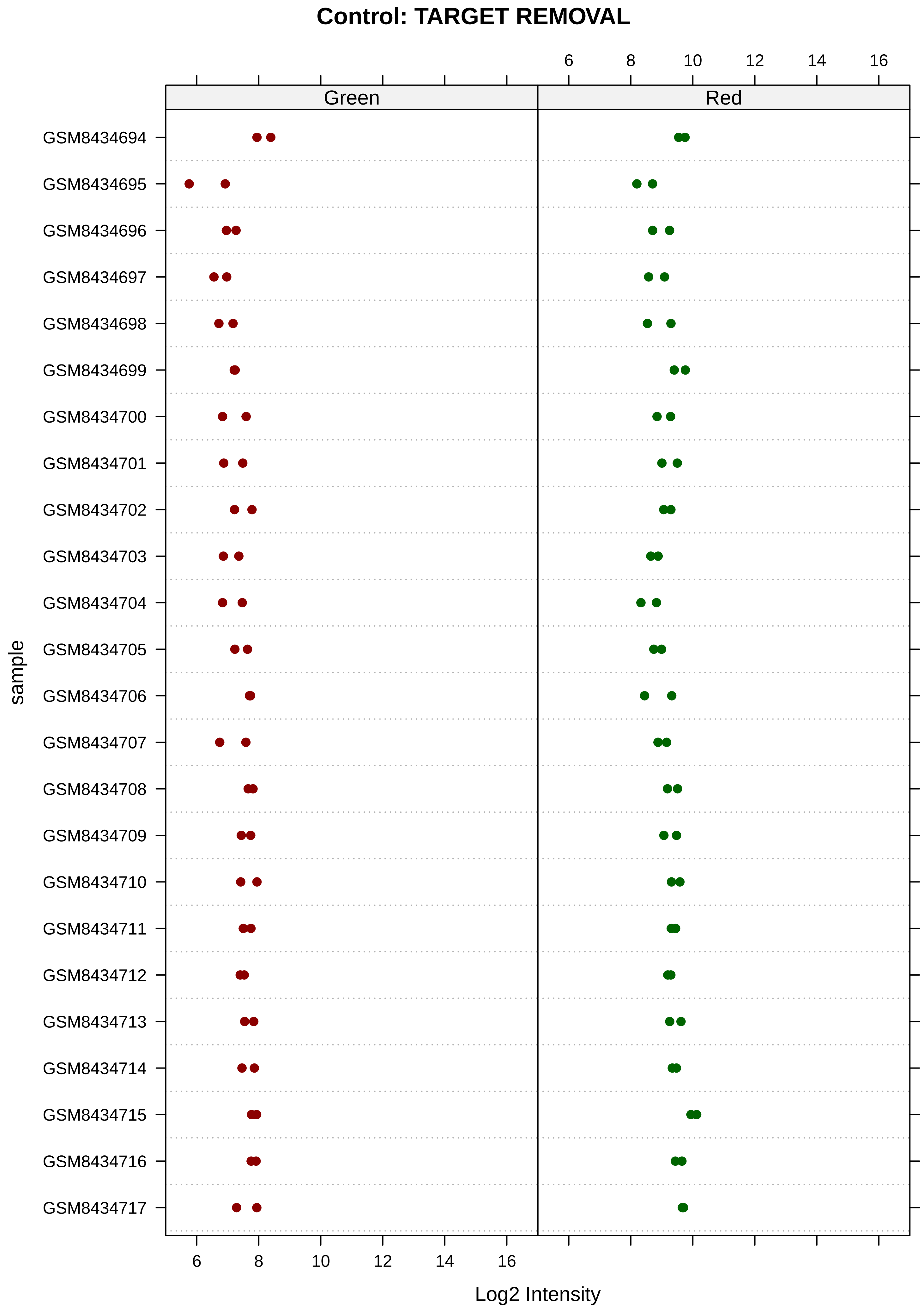

Control: TARGET REMOVAL
Green
Red
sample
Log2 Intensity
GSM8434694
GSM8434695
GSM8434696
GSM8434697
GSM8434698
GSM8434699
GSM8434700
GSM8434701
GSM8434702
GSM8434703
GSM8434704
GSM8434705
GSM8434706
GSM8434707
GSM8434708
GSM8434709
GSM8434710
GSM8434711
GSM8434712
GSM8434713
GSM8434714
GSM8434715
GSM8434716
GSM8434717
6
8
10
12
14
16

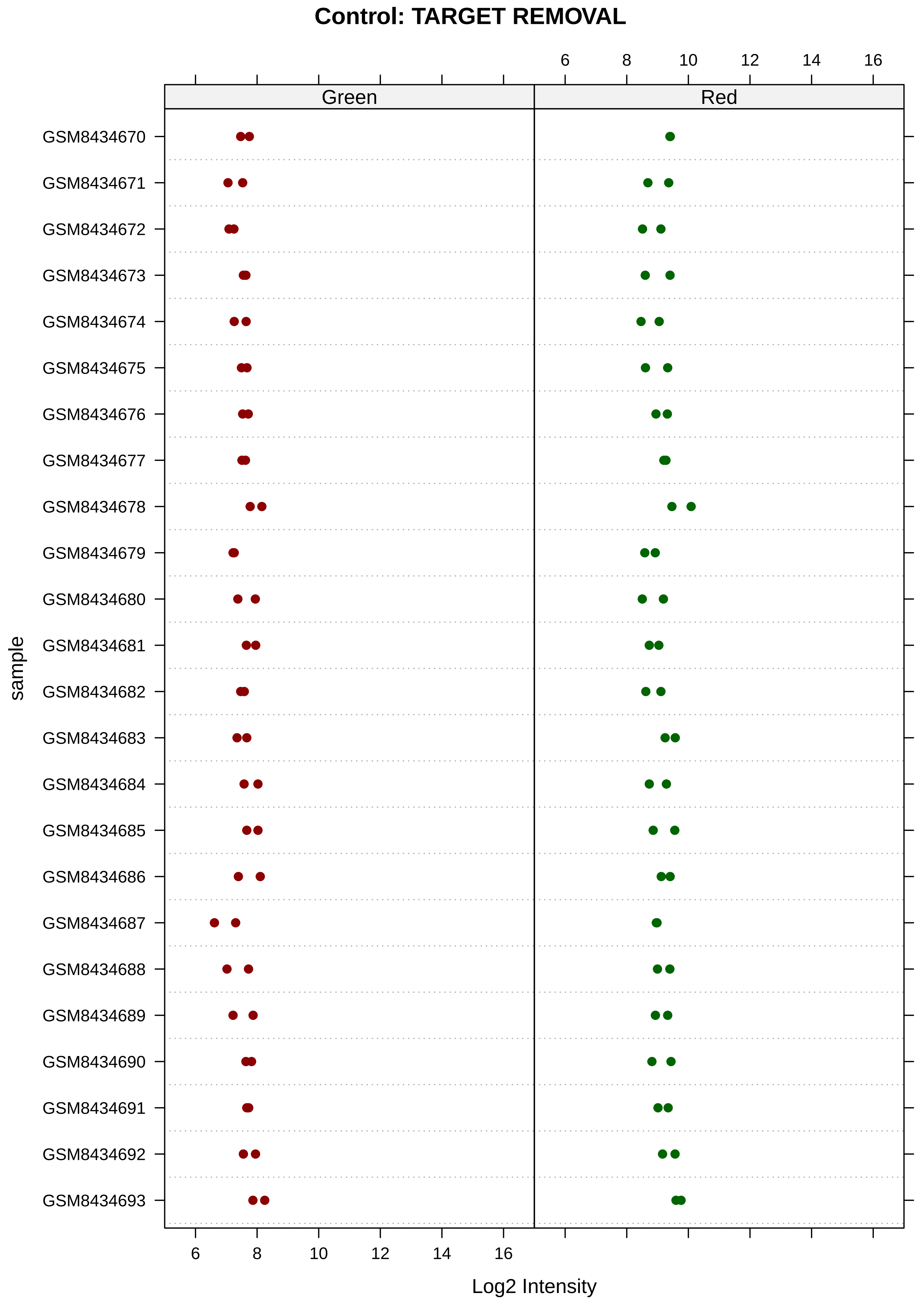

Control: TARGET REMOVAL
Green
Red
sample
Log2 Intensity
GSM8434670
GSM8434671
GSM8434672
GSM8434673
GSM8434674
GSM8434675
GSM8434676
GSM8434677
GSM8434678
GSM8434679
GSM8434680
GSM8434681
GSM8434682
GSM8434683
GSM8434684
GSM8434685
GSM8434686
GSM8434687
GSM8434688
GSM8434689
GSM8434690
GSM8434691
GSM8434692
GSM8434693
6
8
10
12
14
16

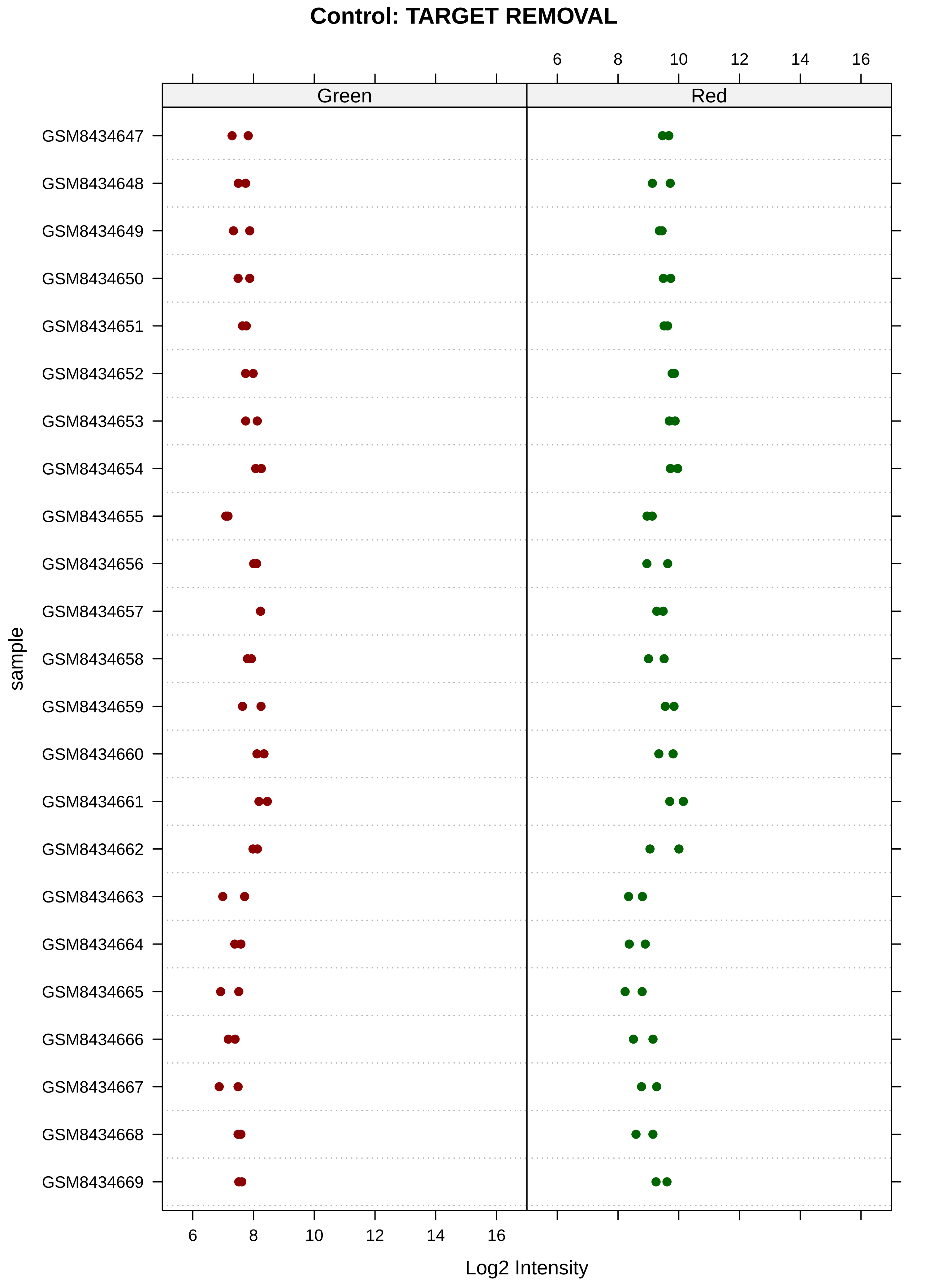

Control: TARGET REMOVAL
Green
Red
sample
Log2 Intensity
GSM8434647
GSM8434648
GSM8434649
GSM8434650
GSM8434651
GSM8434652
GSM8434653
GSM8434654
GSM8434655
GSM8434656
GSM8434657
GSM8434658
GSM8434659
GSM8434660
GSM8434661
GSM8434662
GSM8434663
GSM8434664
GSM8434665
GSM8434666
GSM8434667
GSM8434668
GSM8434669
6
8
10
12
14
16

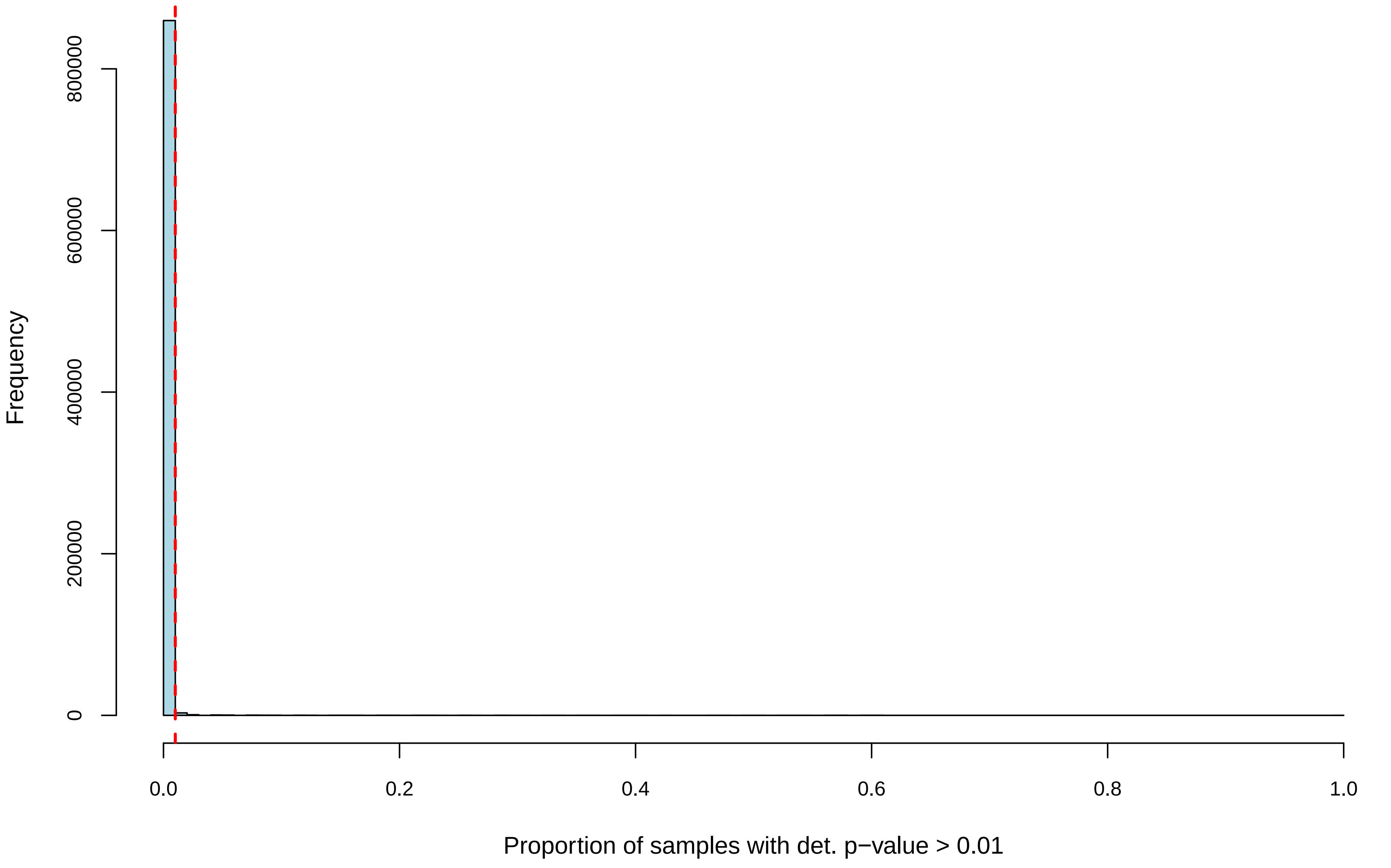

Frequency
0
200000
400000
600000
800000
0.0
0.2
0.4
0.6
0.8
1.0
Proportion of samples with det. p−value > 0.01

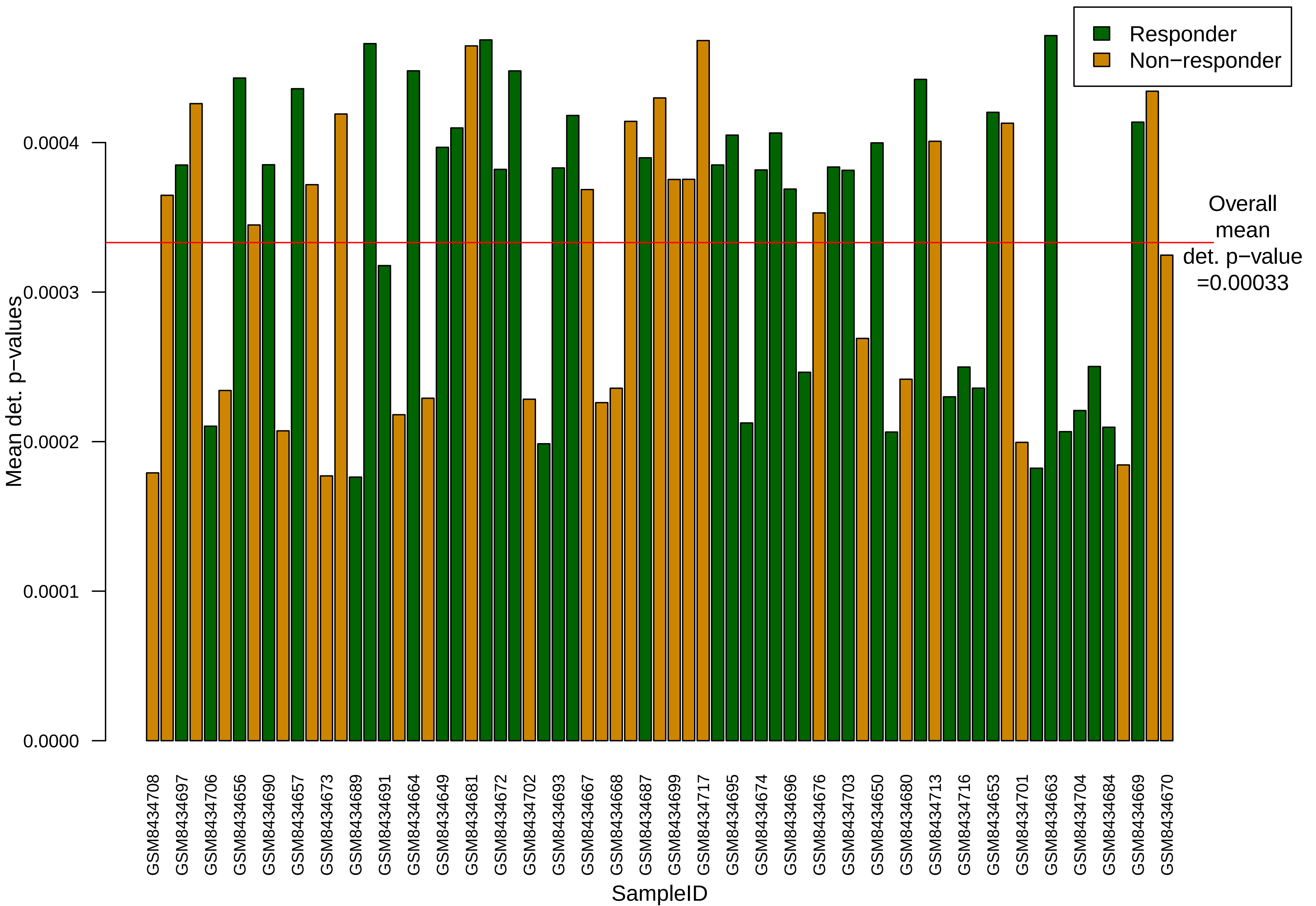
Responder
Non−responder
Overall mean det. p−value =0.00033
Mean det. p−values
0.0000
0.0001
0.0002
0.0003
0.0004
SampleID
GSM8434708
GSM8434697
GSM8434706
GSM8434656
GSM8434690
GSM8434657
GSM8434673
GSM8434689
GSM8434691
GSM8434664
GSM8434649
GSM8434681
GSM8434672
GSM8434702
GSM8434693
GSM8434667
GSM8434668
GSM8434687
GSM8434699
GSM8434717
GSM8434695
GSM8434674
GSM8434696
GSM8434676
GSM8434703
GSM8434650
GSM8434680
GSM8434713
GSM8434716
GSM8434653
GSM8434701
GSM8434663
GSM8434704
GSM8434684
GSM8434669
GSM8434670

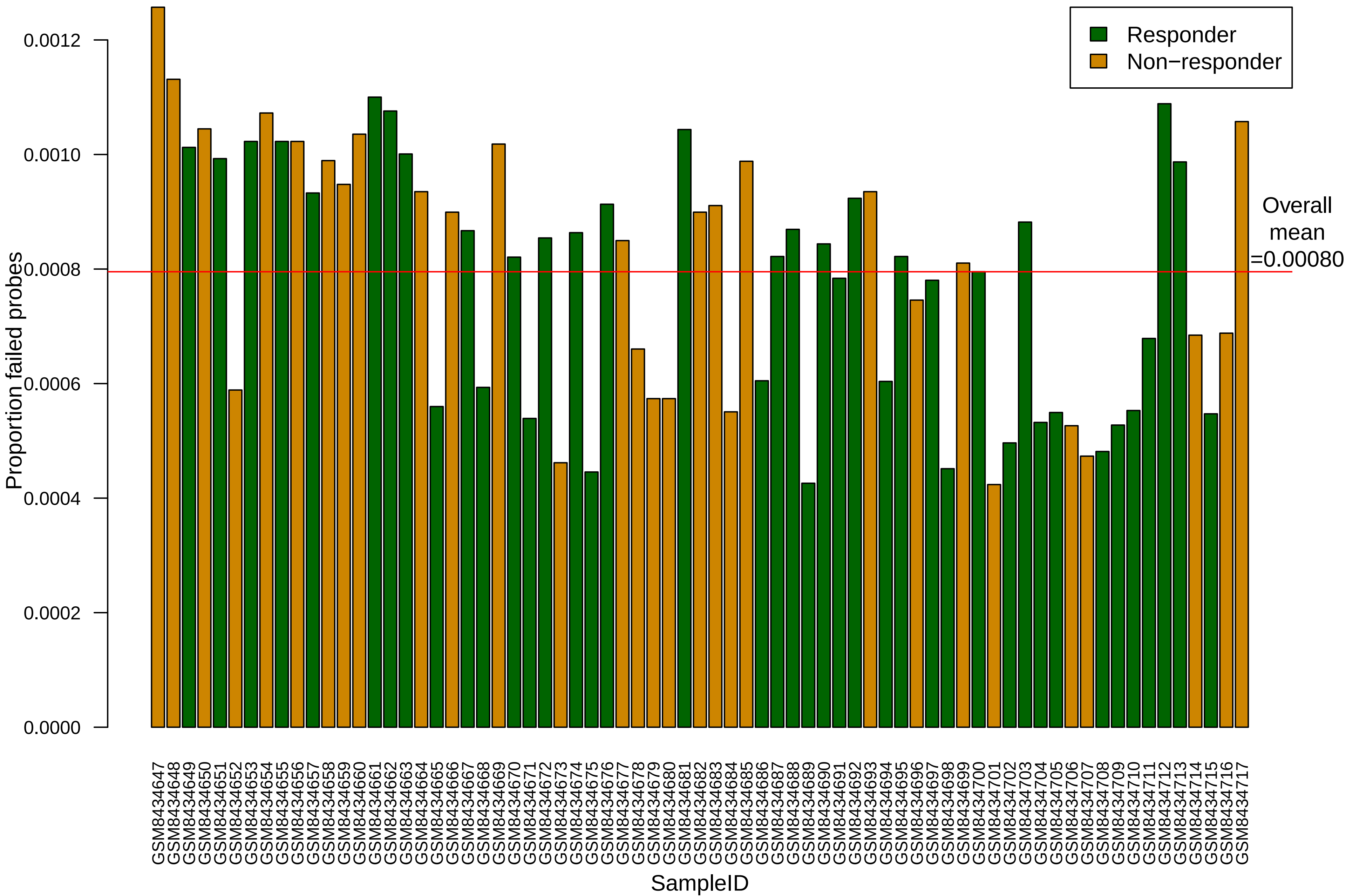

Responder
Non−responder
Overall mean =0.00080
Proportion failed probes
0.0000
0.0002
0.0004
0.0006
0.0008
0.0010
0.0012
SampleID
GSM8434647
GSM8434648
GSM8434649
GSM8434650
GSM8434651
GSM8434652
GSM8434653
GSM8434654
GSM8434655
GSM8434656
GSM8434657
GSM8434658
GSM8434659
GSM8434660
GSM8434661
GSM8434662
GSM8434663
GSM8434664
GSM8434665
GSM8434666
GSM8434667
GSM8434668
GSM8434669
GSM8434670
GSM8434671
GSM8434672
GSM8434673
GSM8434674
GSM8434675
GSM8434676
GSM8434677
GSM8434678
GSM8434679
GSM8434680
GSM8434681
GSM8434682
GSM8434683
GSM8434684
GSM8434685
GSM8434686
GSM8434687
GSM8434688
GSM8434689
GSM8434690
GSM8434691
GSM8434692
GSM8434693
GSM8434694
GSM8434695
GSM8434696
GSM8434697
GSM8434698
GSM8434699
GSM8434700
GSM8434701
GSM8434702
GSM8434703
GSM8434704
GSM8434705
GSM8434706
GSM8434707
GSM8434708
GSM8434709
GSM8434710
GSM8434711
GSM8434712
GSM8434713
GSM8434714
GSM8434715
GSM8434716
GSM8434717
