# Supplementary material for: B-cell DNA methylation signature in response to hepatitis B virus vaccination in females and males
Source: Front Immunol. 2026 Apr 10;17:1734384. doi: 10.3389/fimmu.2026.1734384 (PMC13105942; doi:10.3389/fimmu.2026.1734384)
Supplement: Supplementary file 3 [file DataSheet3.pdf]

Table T1. List of public datasets used in evaluation of correlations between gene methylation and expression. Correlation analysis was performed for main results i.e. 8 genes visualized and commented in main body of the manuscript: *IKZF1*, *SMAD3*, *FGF1*, *ZBTB20*, *TNIP3*, *ITPR2*, *SARDH* and *BTG3*. Correlations were evaluated with linear regression model. All significant correlations (p-value < 0.05) are summarized on the plots below.

GEO NCBI Accession Number	Number of control samples	Biological material	Female %	Average age ± Standard deviation	Methylation assay	Expression assay	Methylation data	Expression data	Reference
GSE71245	4	B cells	100 %	43.5 ± 9.7	Illumina HM450 BeadChip	Illumina HumanHT-12 V4.0	Methylation values were retrieved from GSE71244_series_matrix.txt file, providing, as reported by authors, normalized values calculated using an R-script pipeline developed by Touleimat and Tost (Epigenomics 4:325-341).	Expression values were retrieved from GSE71115_series_matrix.txt file, providing, as reported by authors, log2 transformed, filtered and normalized values.	(1)
GSE165083	14	Whole blood	53 %	68.0 ± 5.2	Illumina HM450 BeadChip	Illumina HiSeq 2000	Methylation values were calculated from raw idat files using our standard preprocessing script including QC cleaning, filtering and normalization steps.	Expression values were retrieved from GSE165082_PD-CC.counts.txt file, providing, as reported by authors, not-normalized values. These counts were depth-normalized to CPM with edgeR package before assessment of correlation.	(2)
GSE181647	9	PBMC	55 %	39.57 ± 7.33	Illumina HMEpic BeadChip	MGISEQ-2000RS	Methylation values were retrieved from GSE181647-GPL21145_series_matrix.txt file, presumably processed by authors with ChAMP R package.	Expression values were retrieved from GSE181646_Expression_matrix.txt file, providing, as reported by authors, not-normalized values. These counts were depth-normalized to CPM with edgeR package before assessment of correlation.	(3)

1. Mamrut S, Avidan N, Staun-Ram E, Ginzburg E, Truffault F, Berrih-Aknin S, et al. Integrative analysis of methylome and transcriptome in human blood identifies extensive sex- and immune cell-specific differentially methylated regions. Epigenetics. 2015;10(10):943–57.

2. Henderson AR, Wang Q, Meechoovet B, Siniard AL, Naymik M, De Both M, et al. DNA Methylation and Expression Profiles of Whole Blood in Parkinson’s Disease. Front Genet. 2021;12:640266.

3. Baek SJ, Ban HJ, Park SM, Kim SY, Lee S, Jin HJ. Genome-wide DNA methylation profiling reveals candidate biomarkers and probable molecular mechanism of metabolic syndrome. Genes Dis. 2022 July;9(4):833–6.

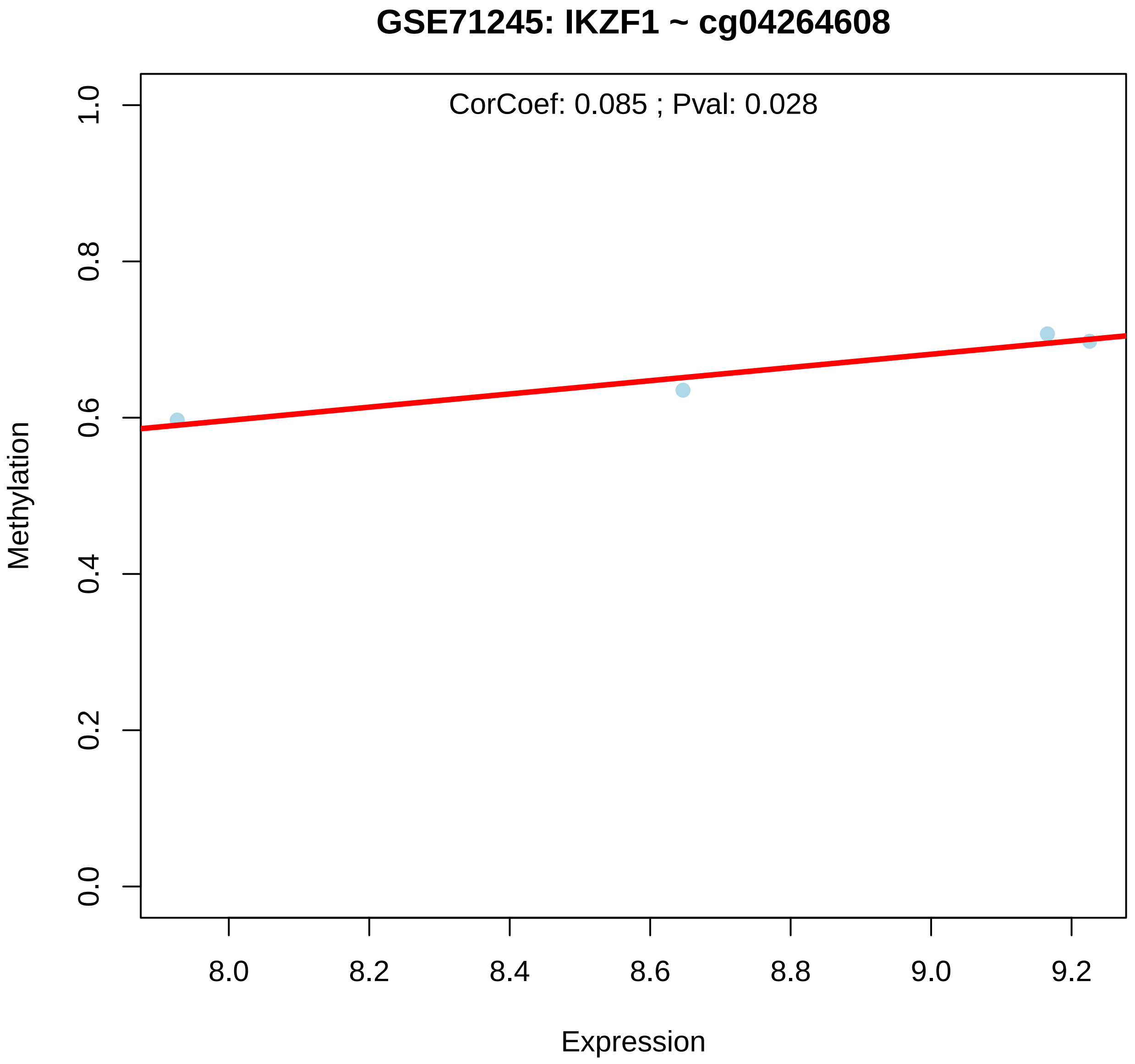

Figure T1. Correlation between DNA methylation and RNA expression in public dataset.

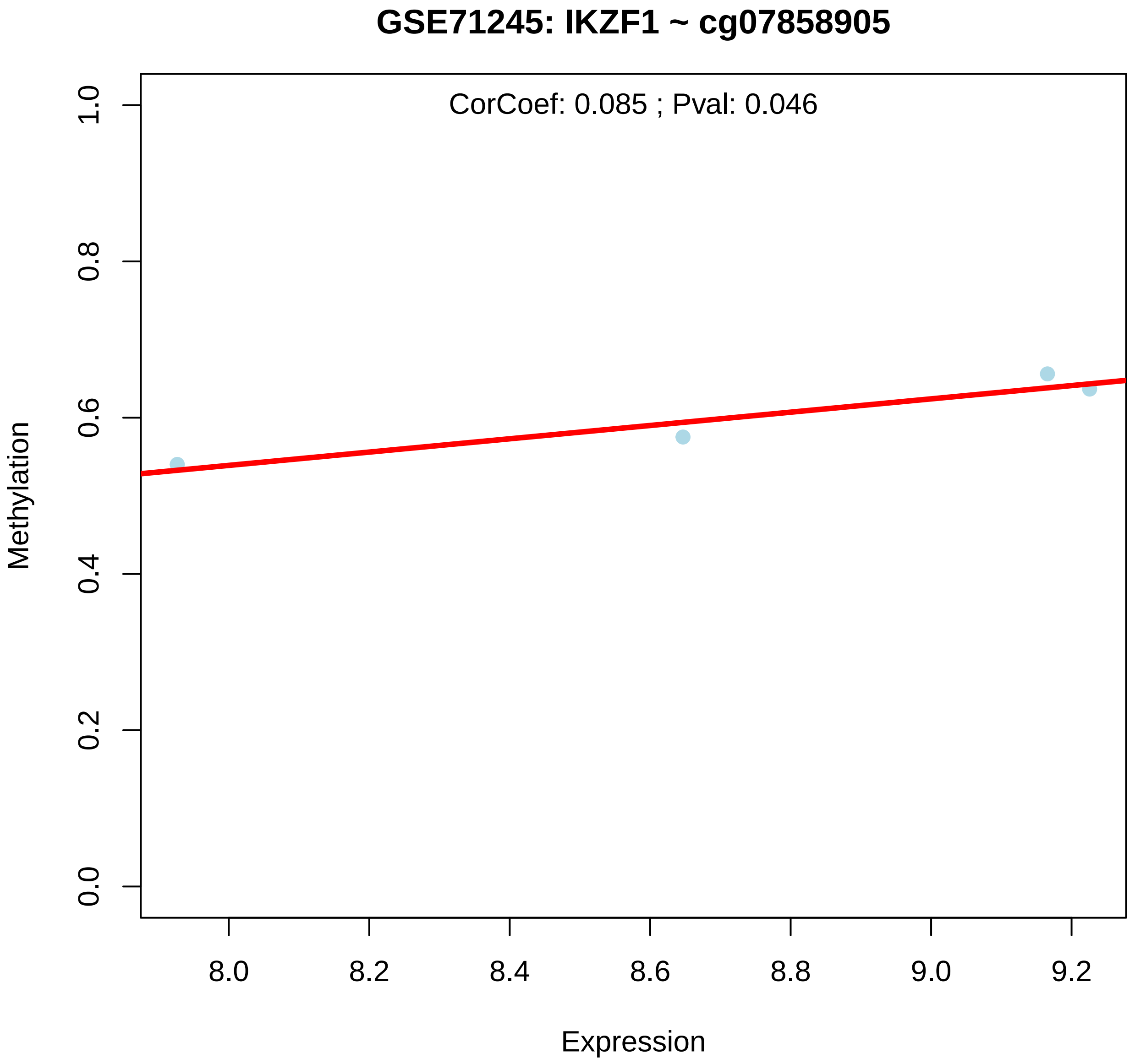

Figure T2. Correlation between DNA methylation and RNA expression in public dataset.

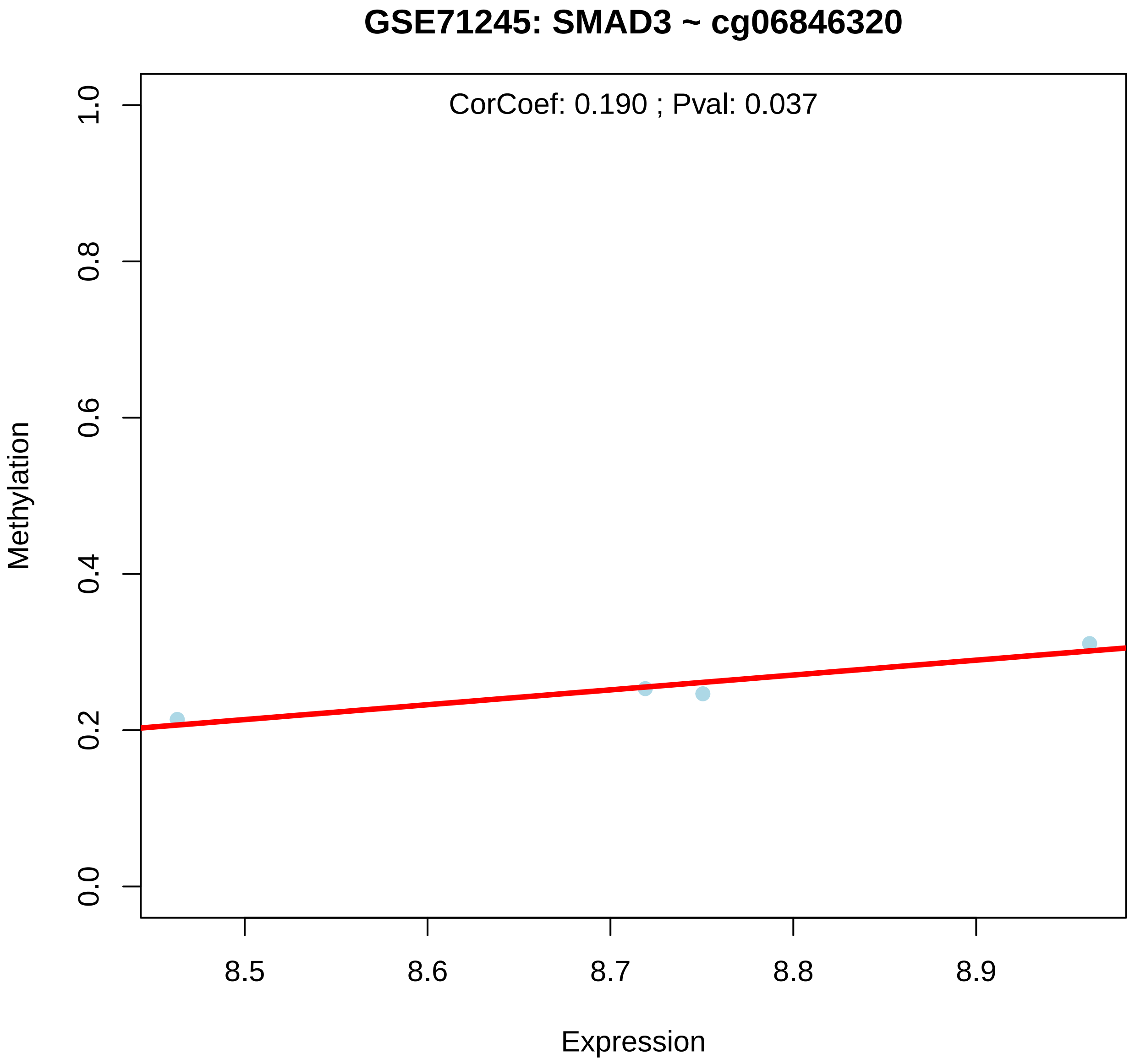

Figure T3. Correlation between DNA methylation and RNA expression in public dataset.

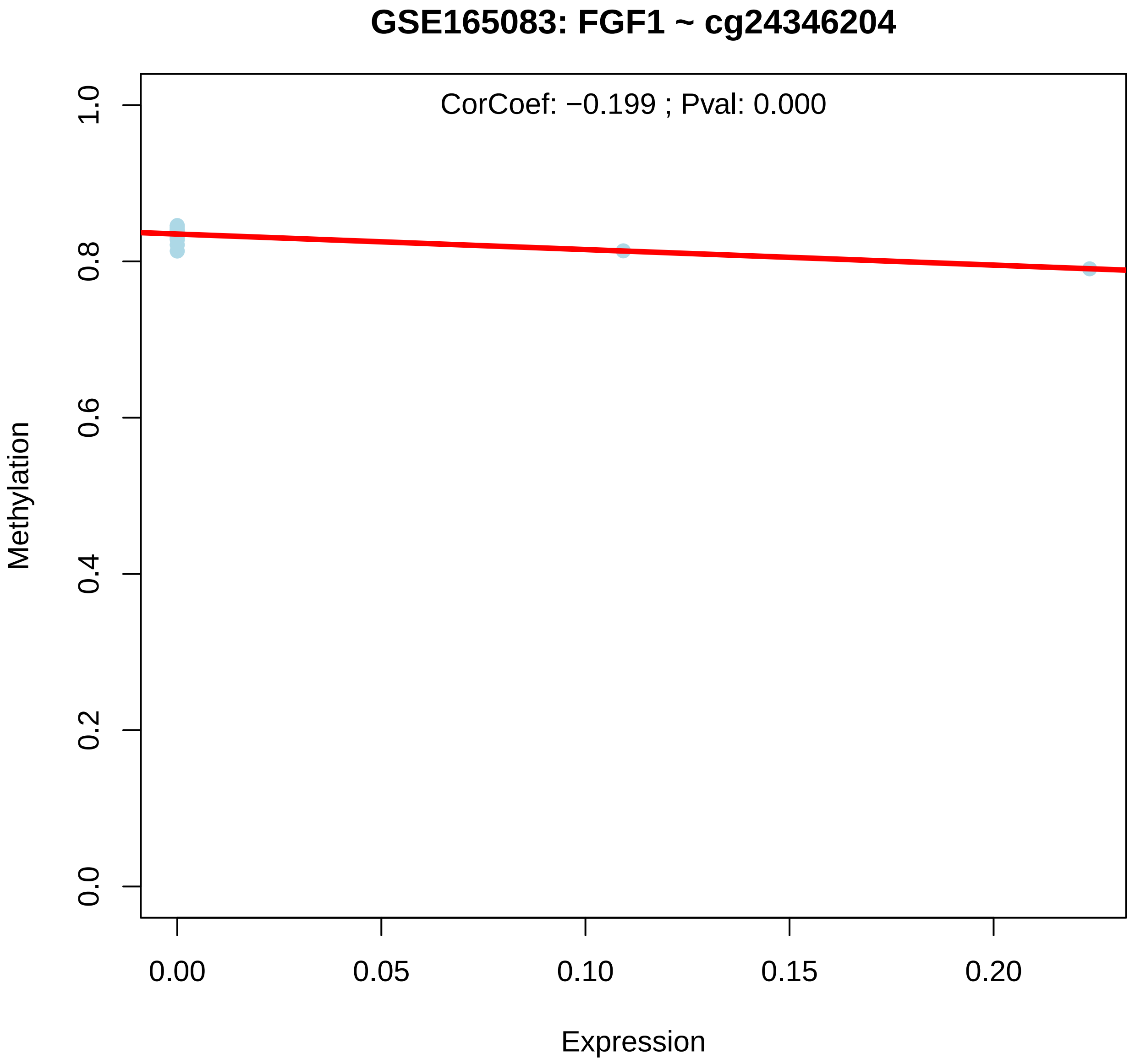

Figure T4. Correlation between DNA methylation and RNA expression in public dataset.

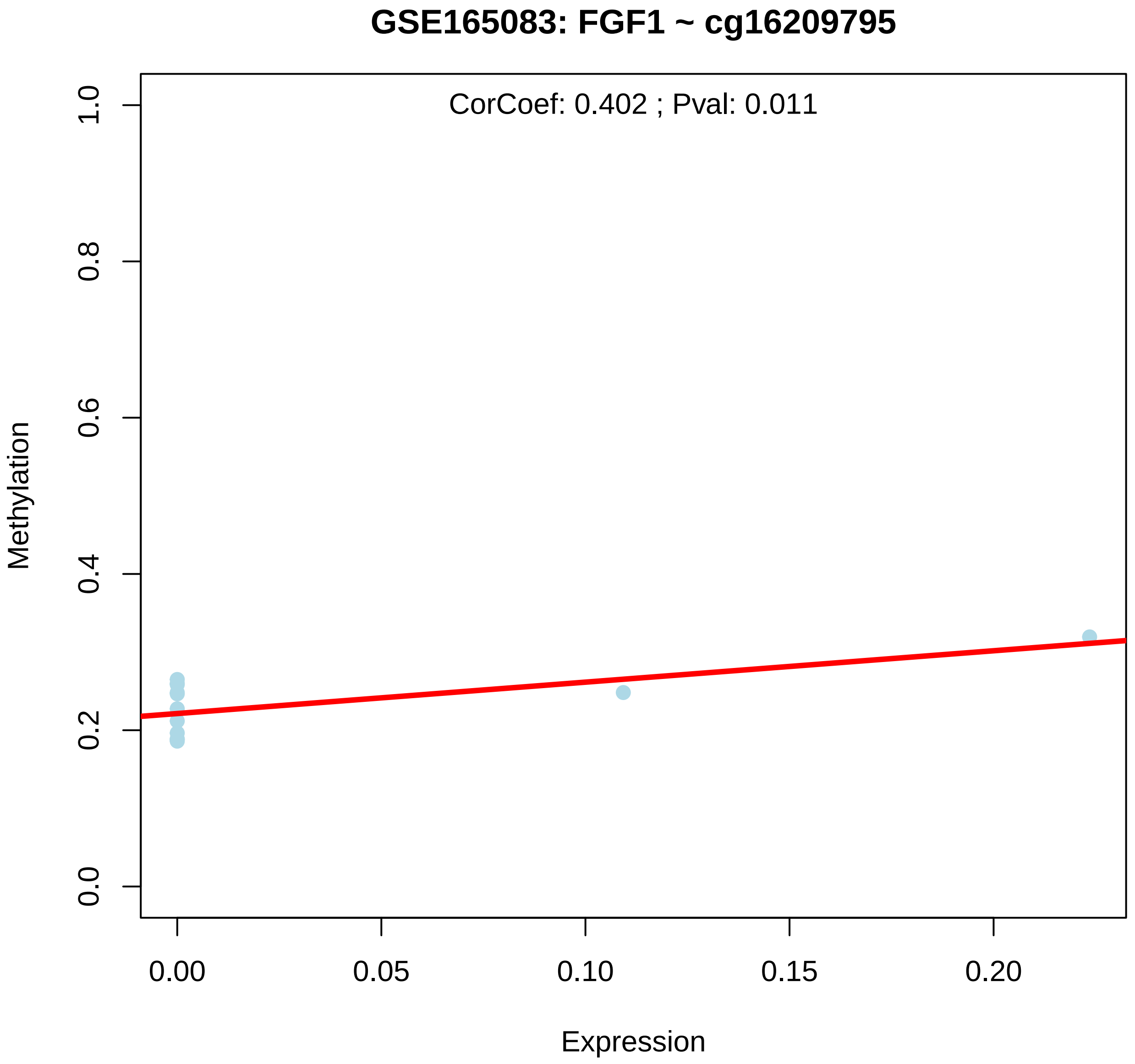

Figure T5. Correlation between DNA methylation and RNA expression in public dataset.

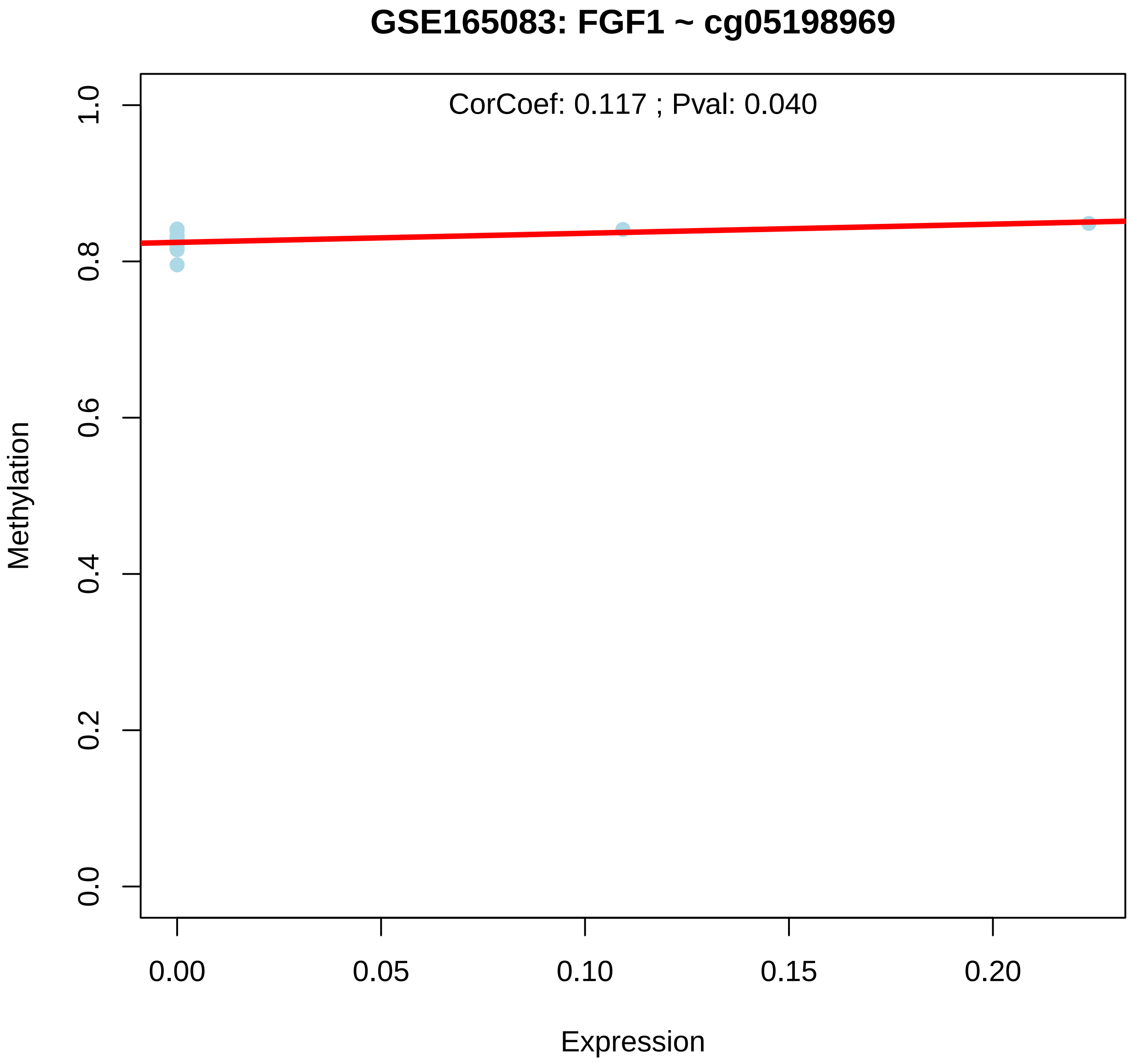

Figure T6. Correlation between DNA methylation and RNA expression in public dataset.

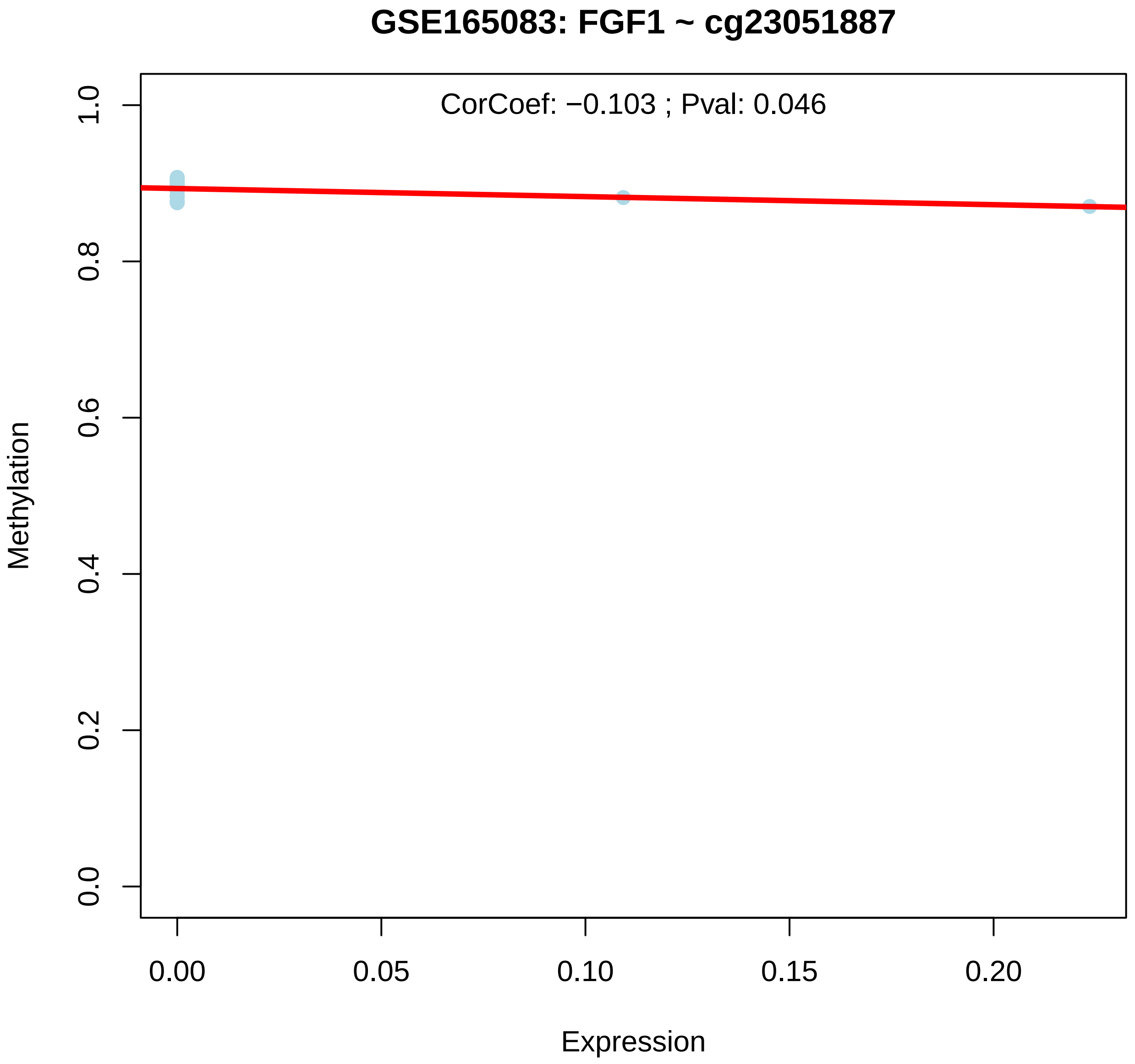

Figure T7. Correlation between DNA methylation and RNA expression in public dataset.

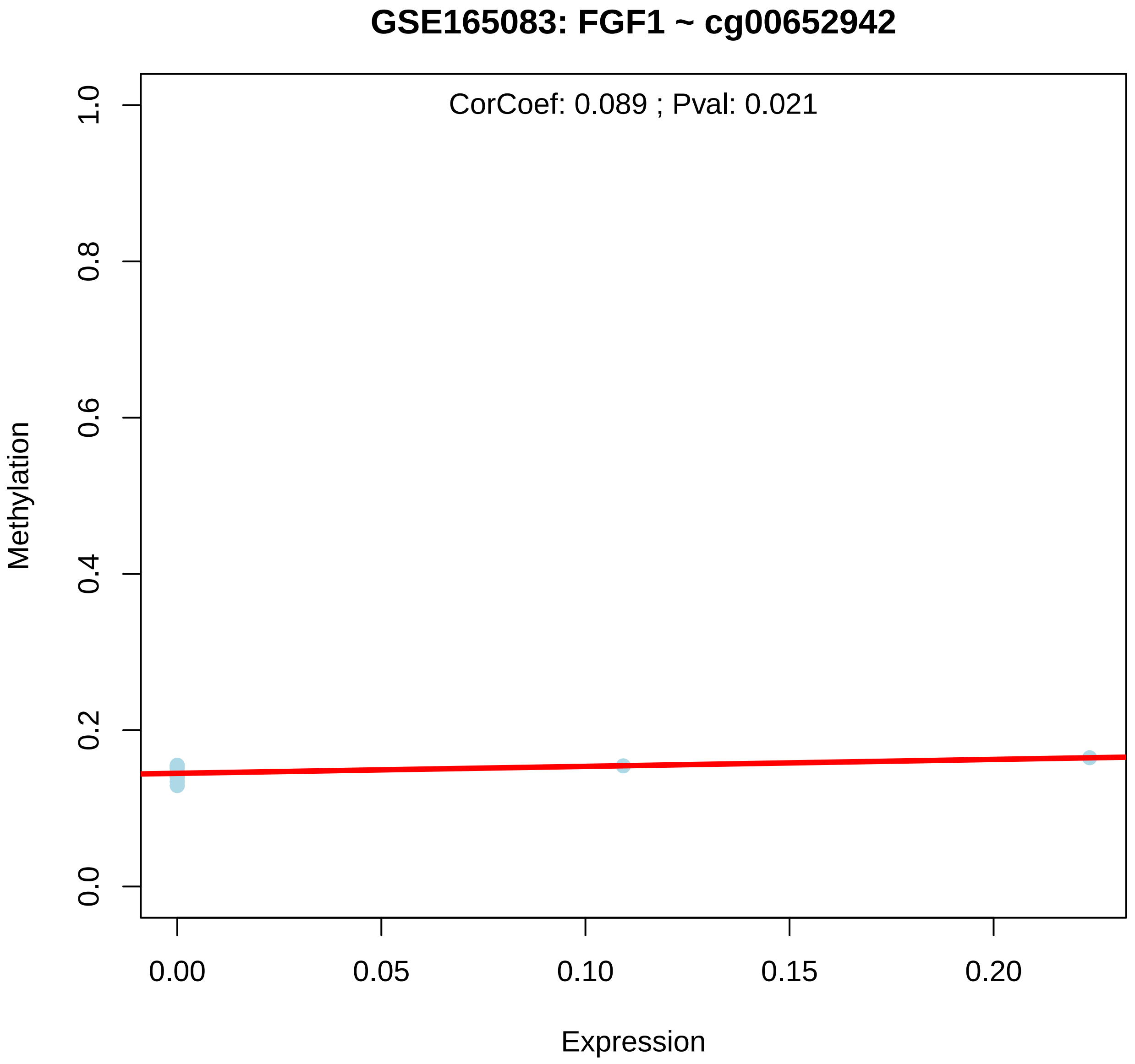

Figure T8. Correlation between DNA methylation and RNA expression in public dataset.

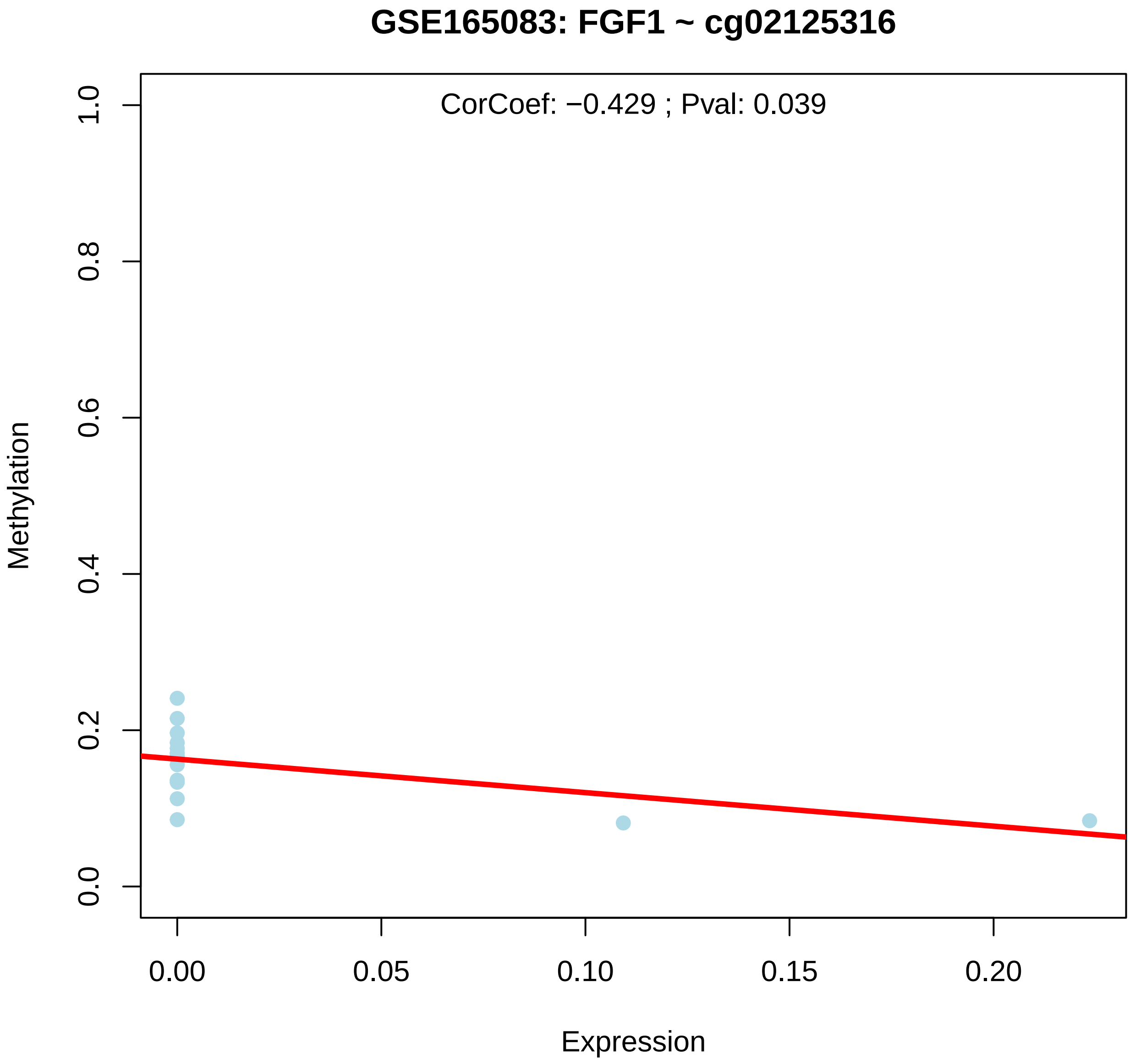

Figure T9. Correlation between DNA methylation and RNA expression in public dataset.

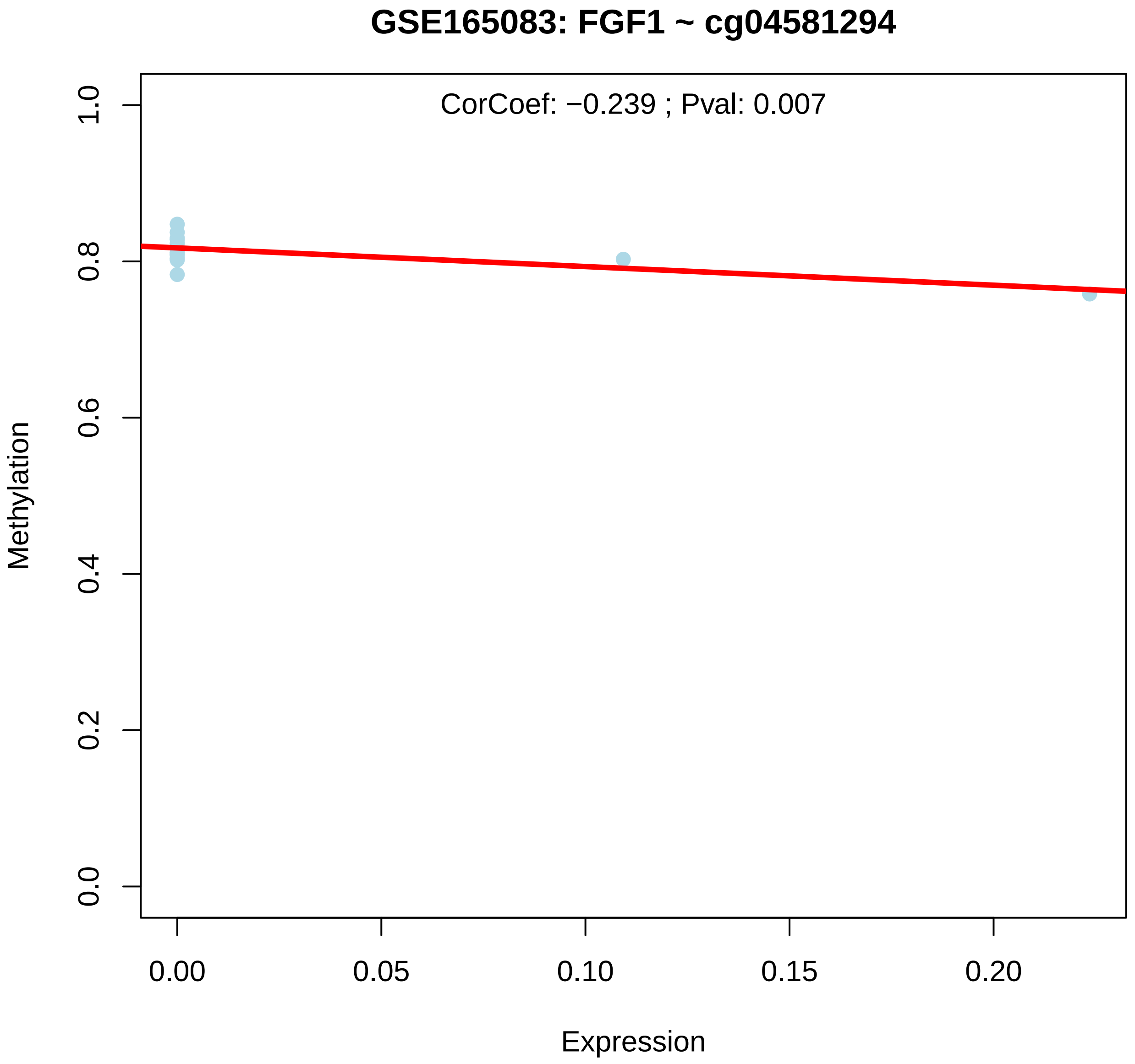

Figure T10. Correlation between DNA methylation and RNA expression in public dataset.

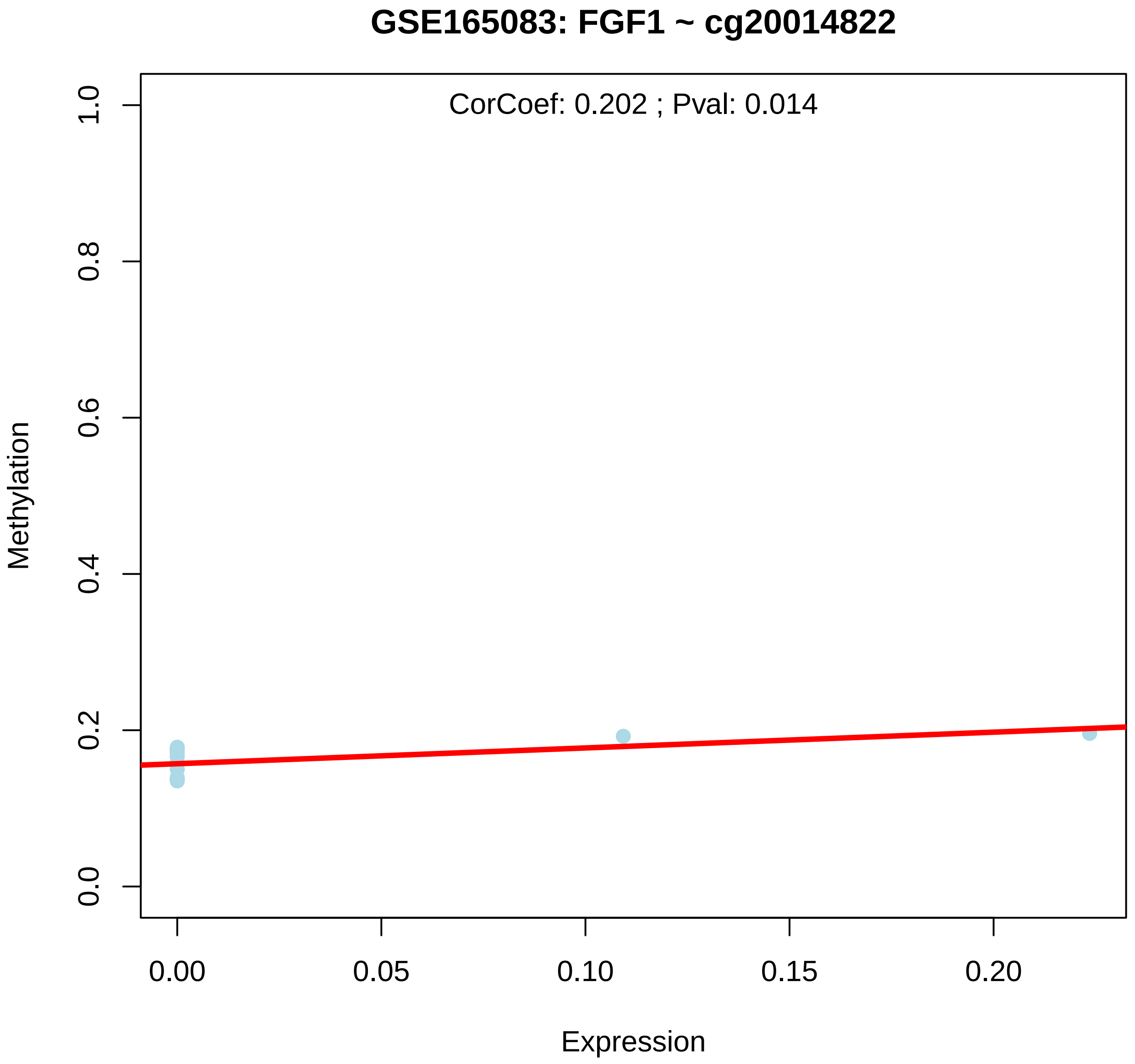

Figure T11. Correlation between DNA methylation and RNA expression in public dataset.

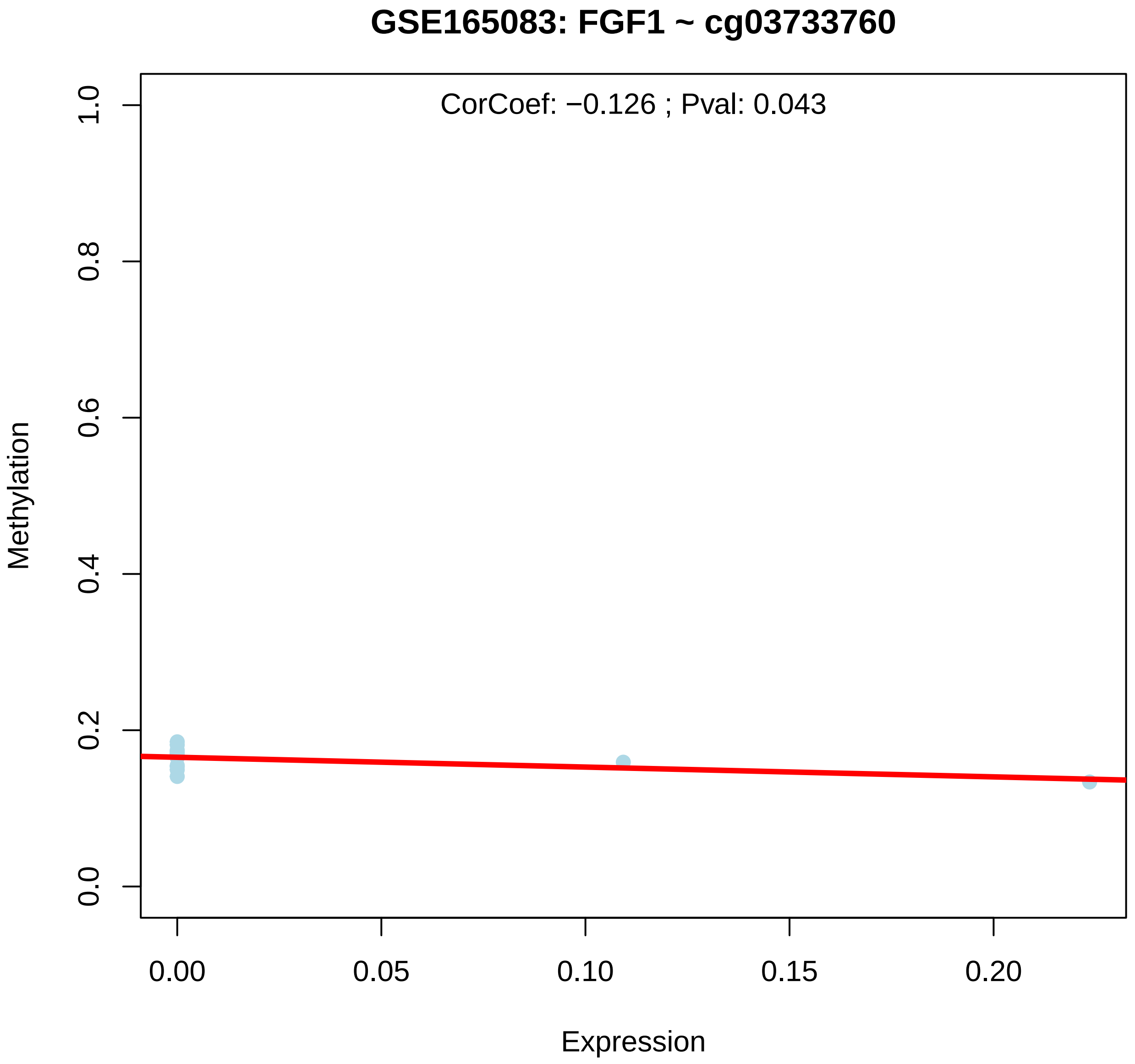

Figure T12. Correlation between DNA methylation and RNA expression in public dataset.

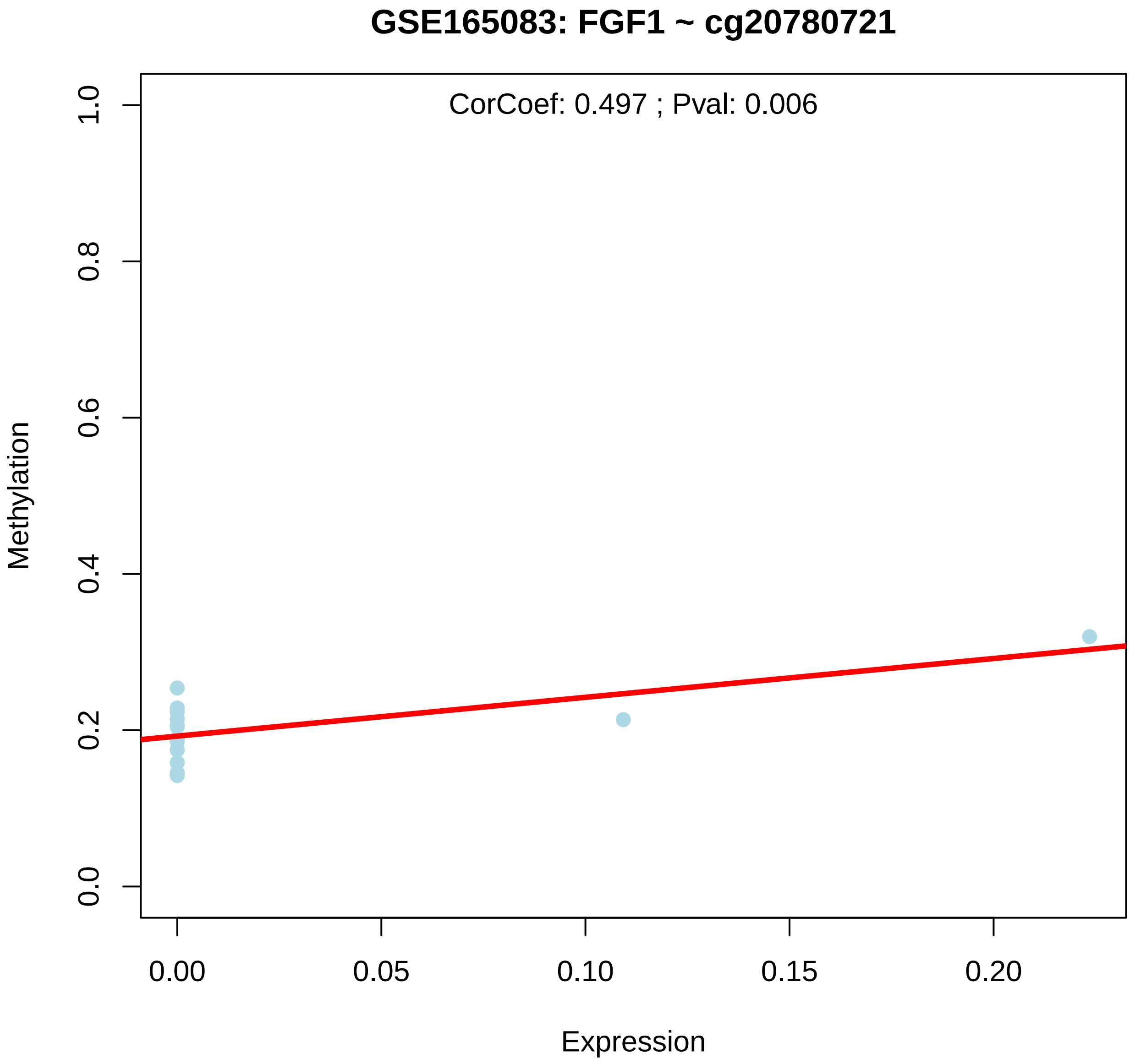

Figure T13. Correlation between DNA methylation and RNA expression in public dataset.

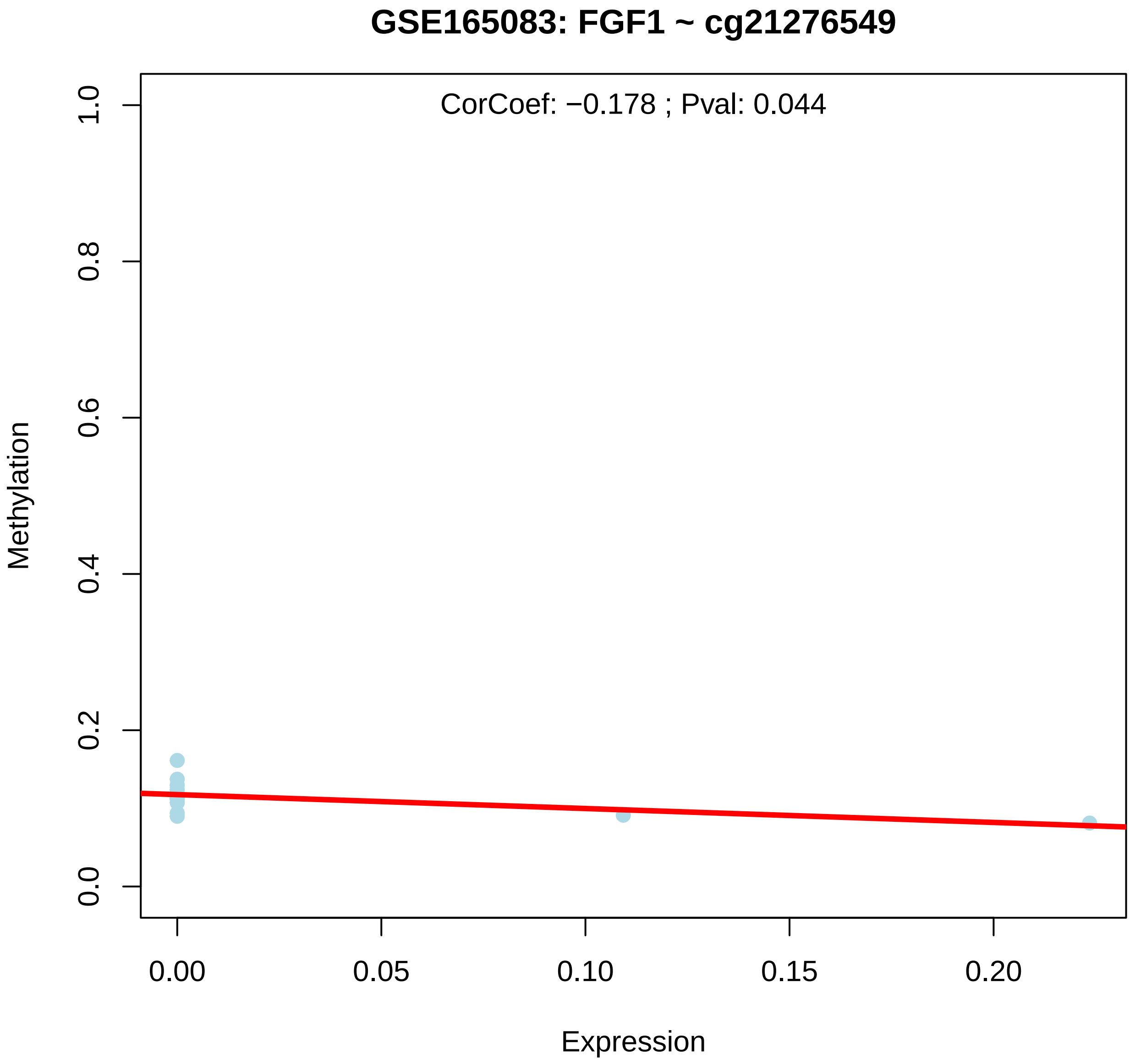

Figure T14. Correlation between DNA methylation and RNA expression in public dataset.

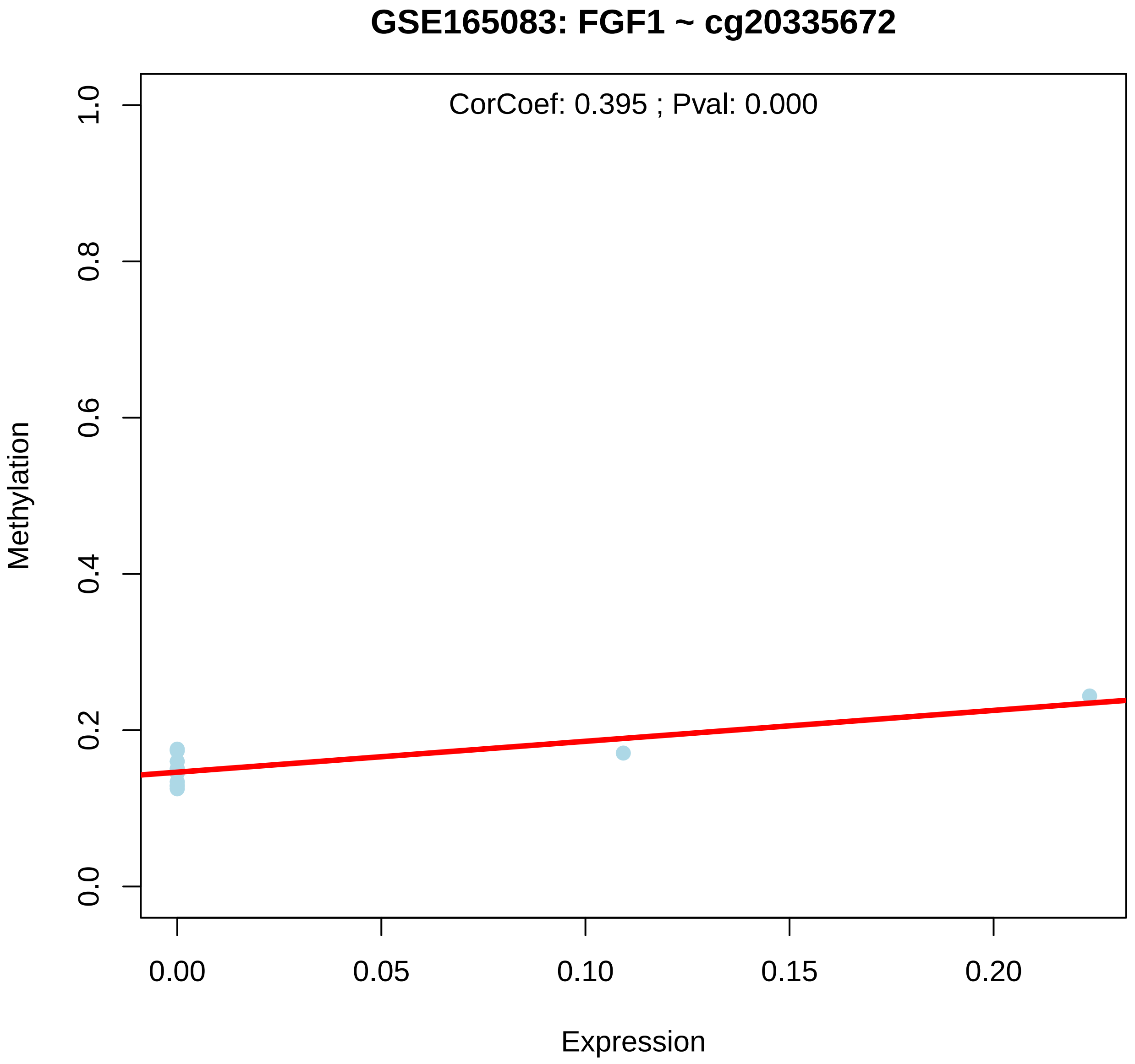

Figure T15. Correlation between DNA methylation and RNA expression in public dataset.

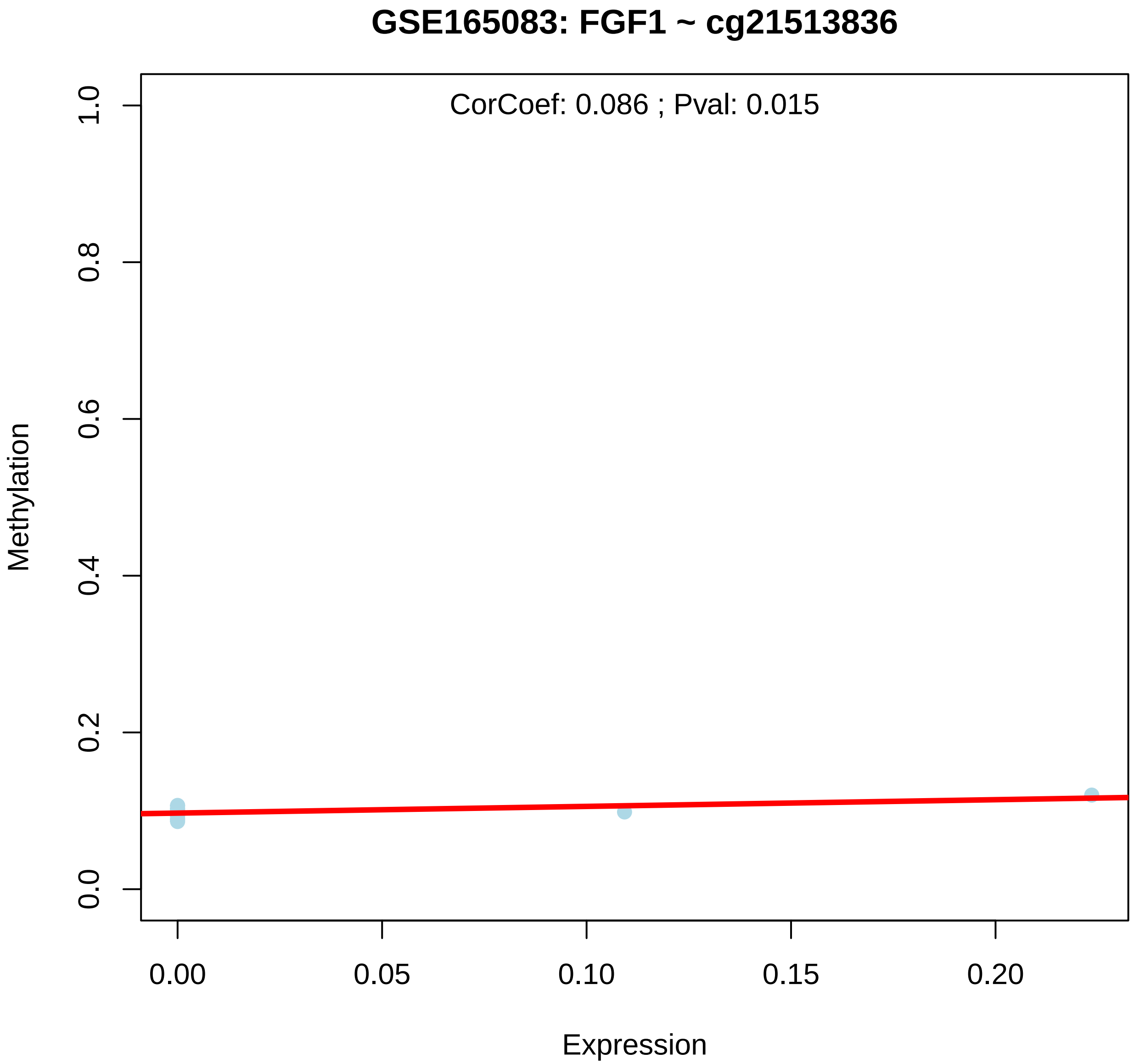

Figure T16. Correlation between DNA methylation and RNA expression in public dataset.

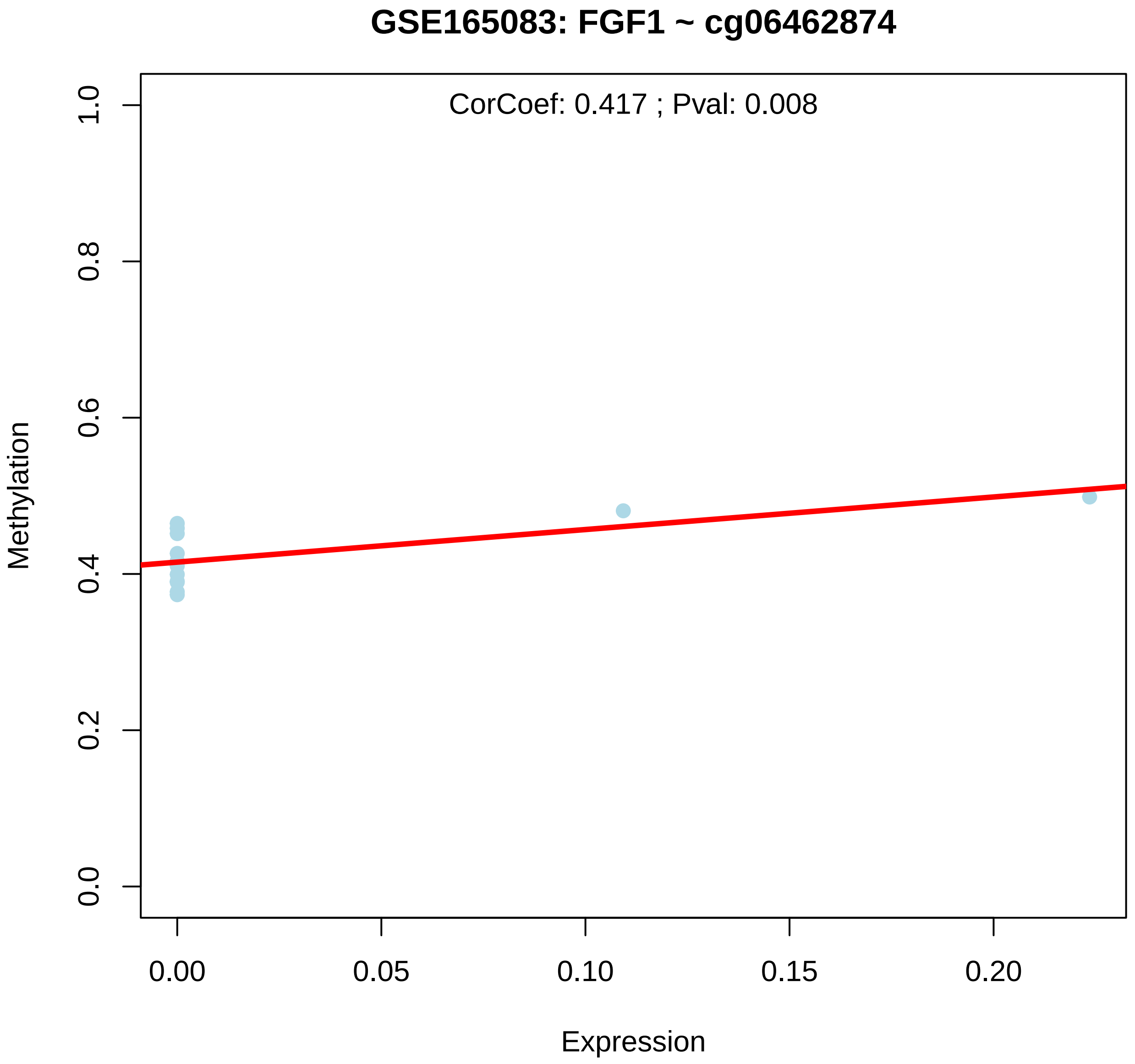

Figure T17. Correlation between DNA methylation and RNA expression in public dataset.

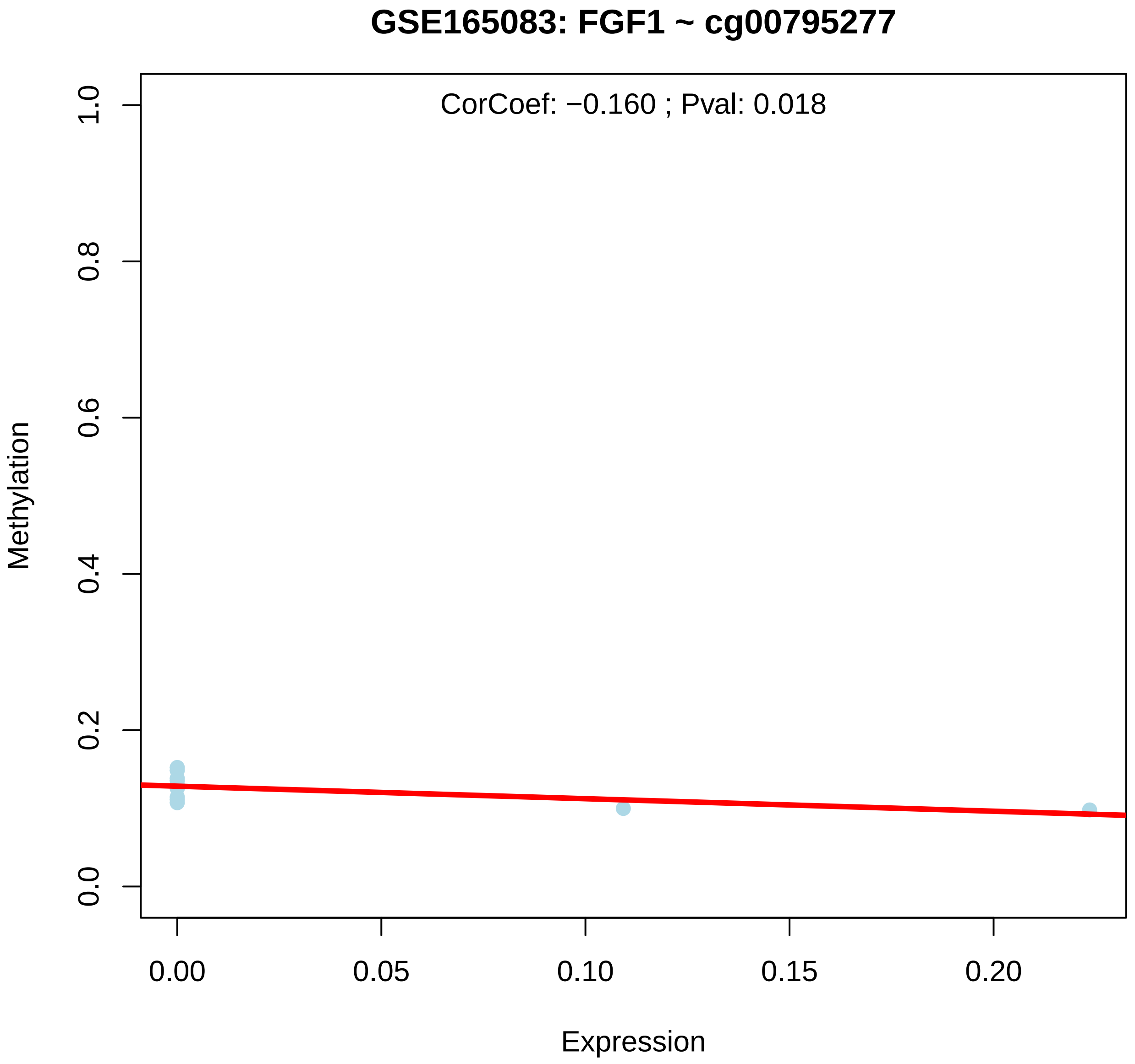

Figure T18. Correlation between DNA methylation and RNA expression in public dataset.

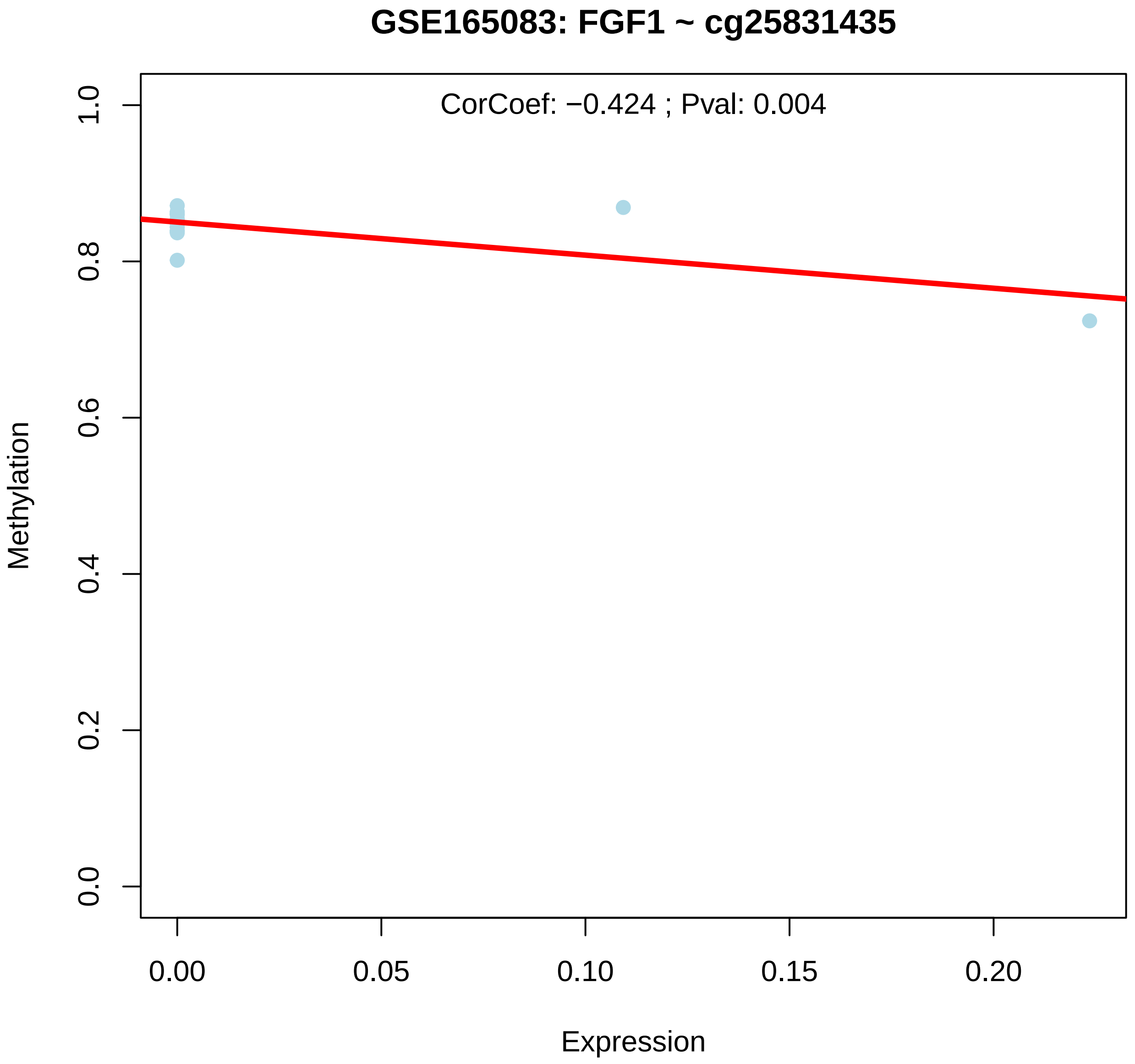

Figure T19. Correlation between DNA methylation and RNA expression in public dataset.

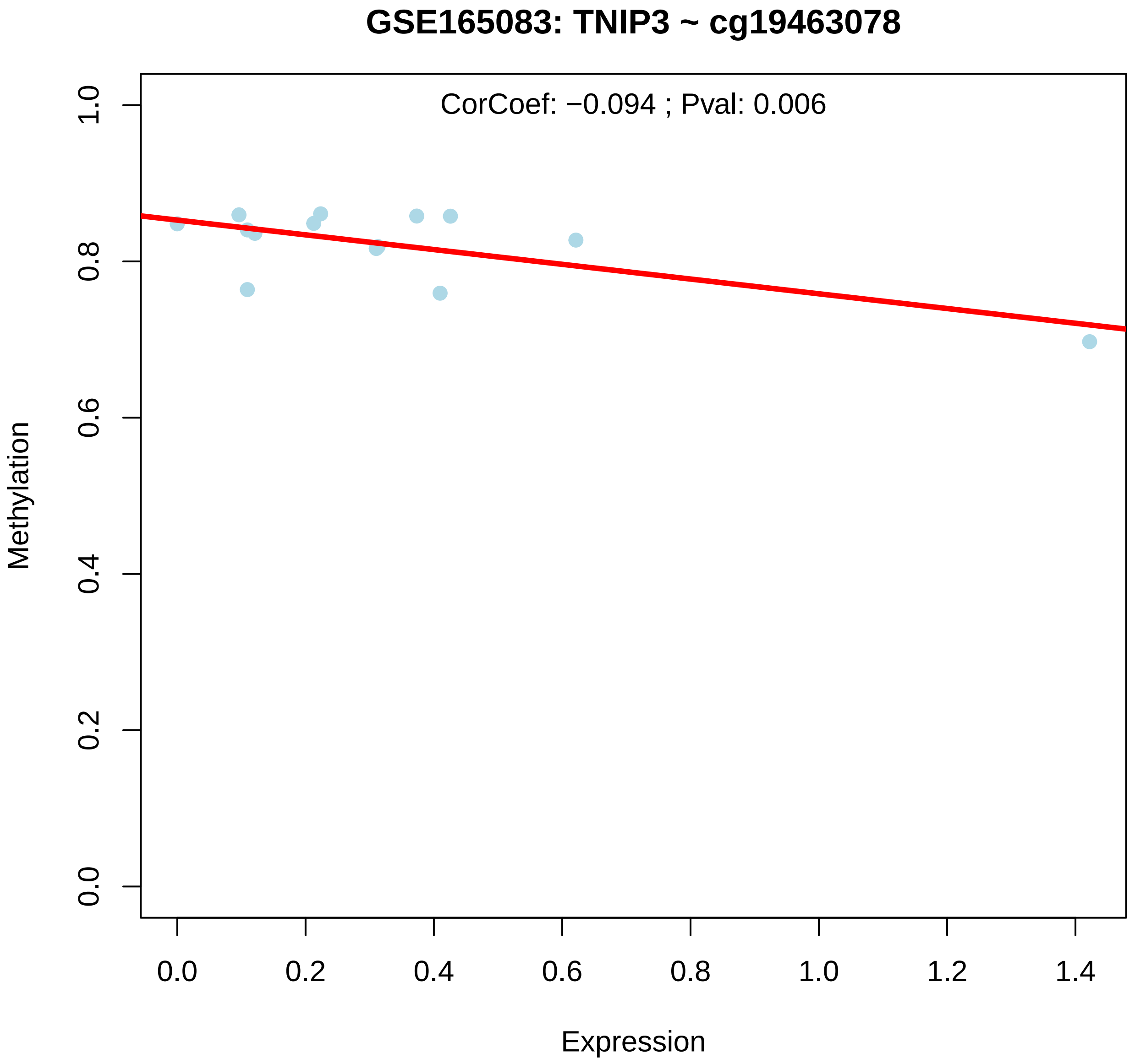

Figure T20. Correlation between DNA methylation and RNA expression in public dataset.

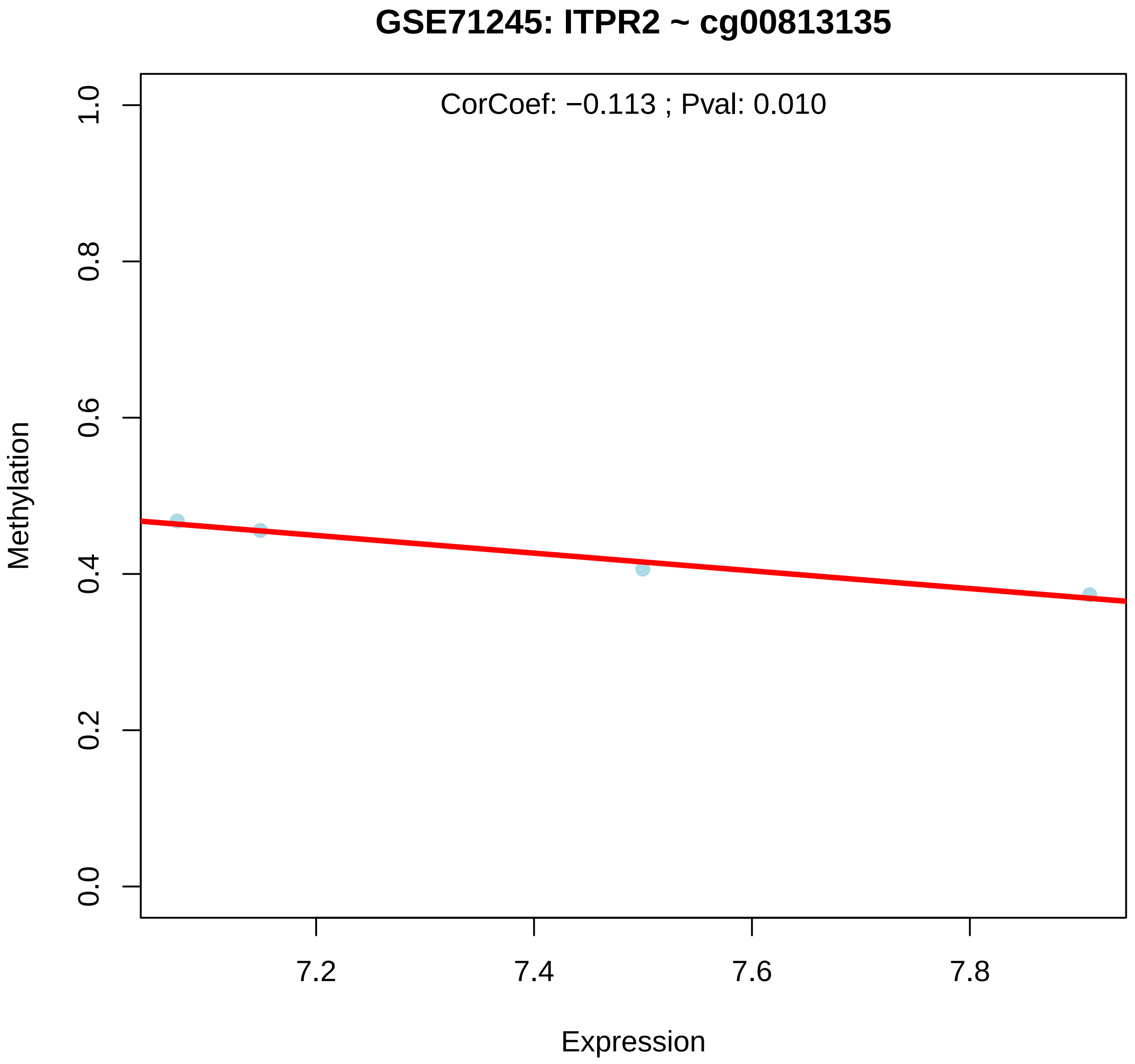

Figure T21. Correlation between DNA methylation and RNA expression in public dataset.

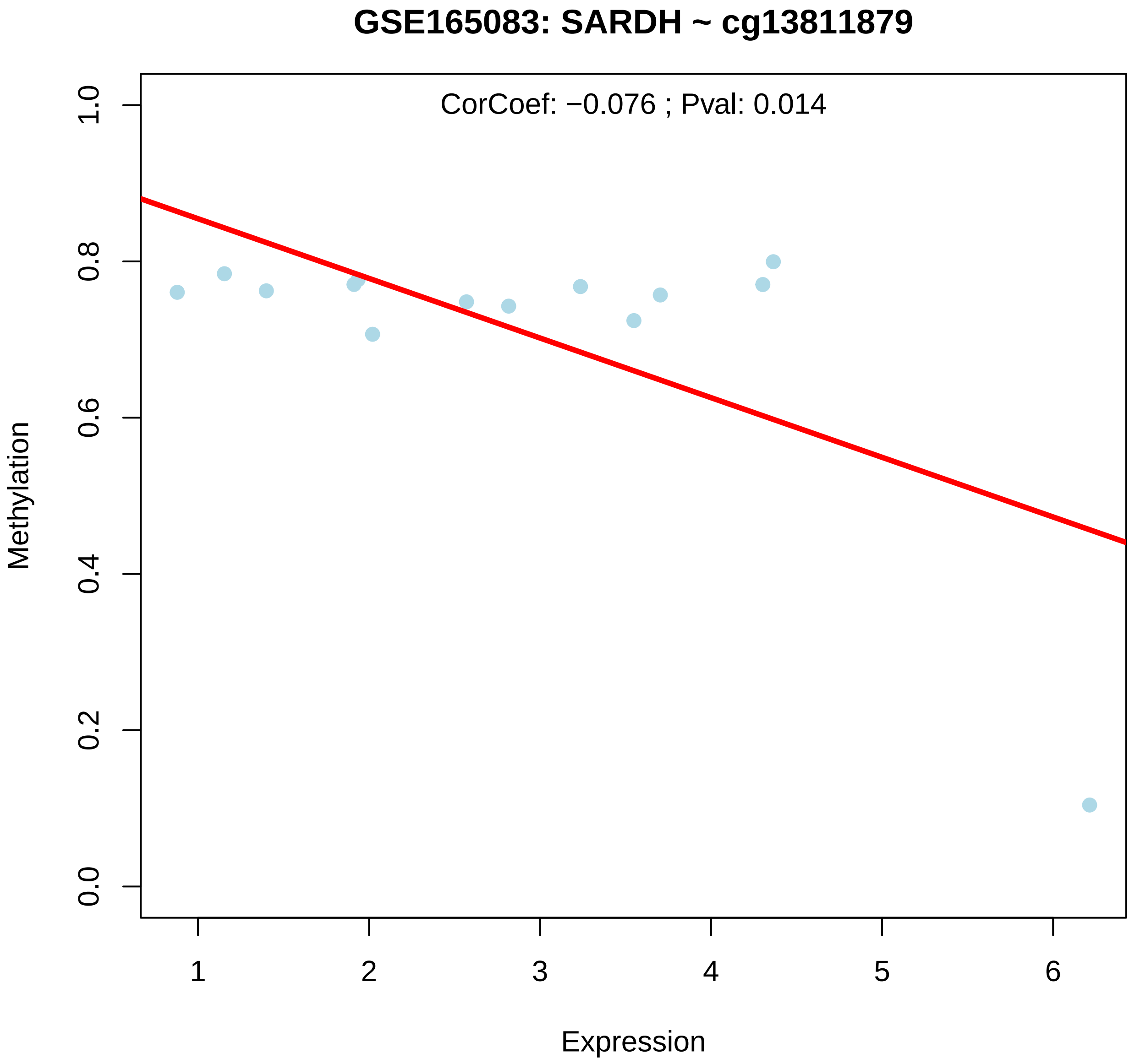

Figure T22. Correlation between DNA methylation and RNA expression in public dataset.

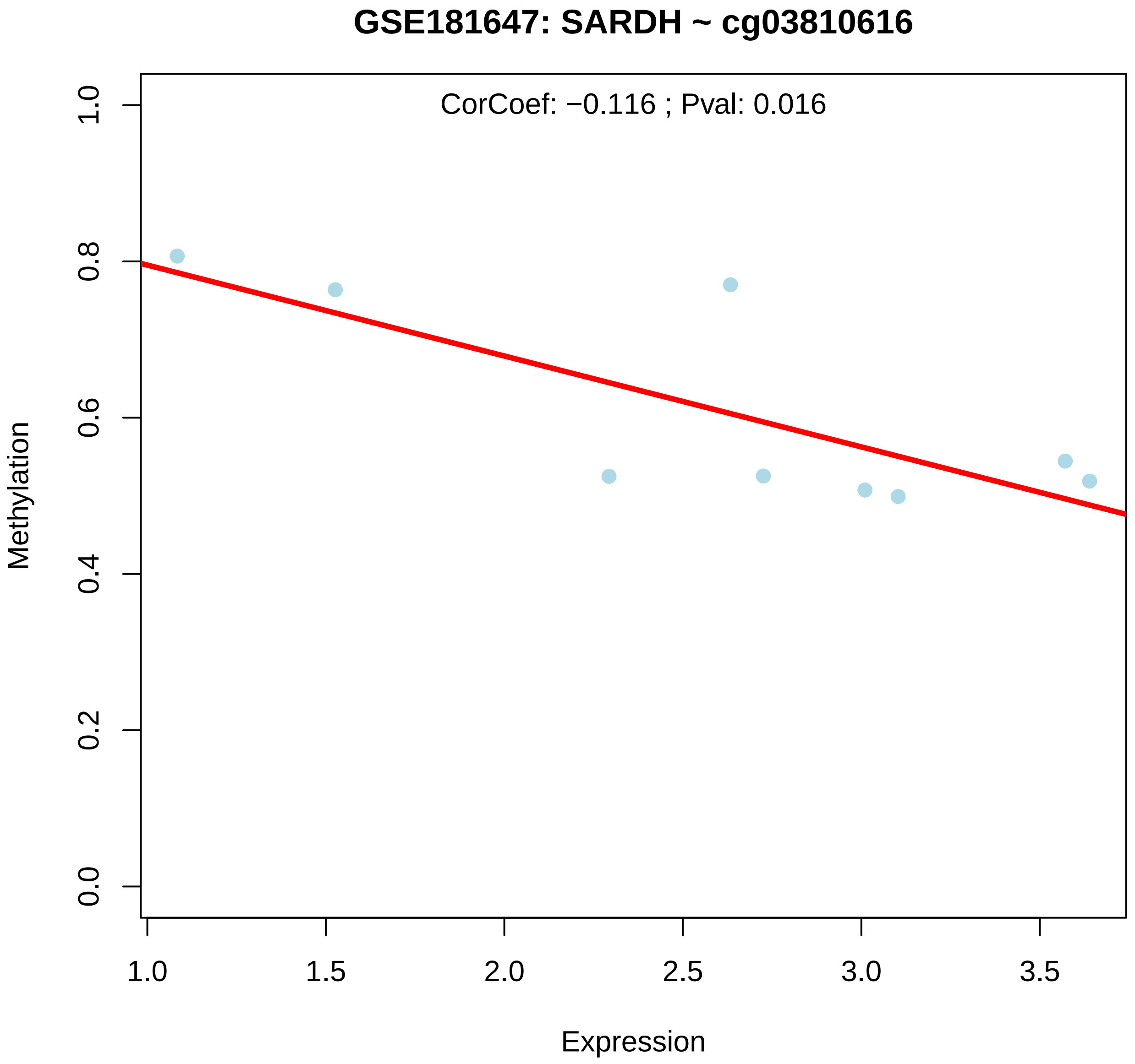

Figure T23. Correlation between DNA methylation and RNA expression in public dataset.
