# Supplementary material for: B-cell DNA methylation signature in response to hepatitis B virus vaccination in females and males
Source: Front Immunol. 2026 Apr 10;17:1734384. doi: 10.3389/fimmu.2026.1734384 (PMC13105942; doi:10.3389/fimmu.2026.1734384)
Supplement: Supplementary file 4 [file DataSheet4.pdf]

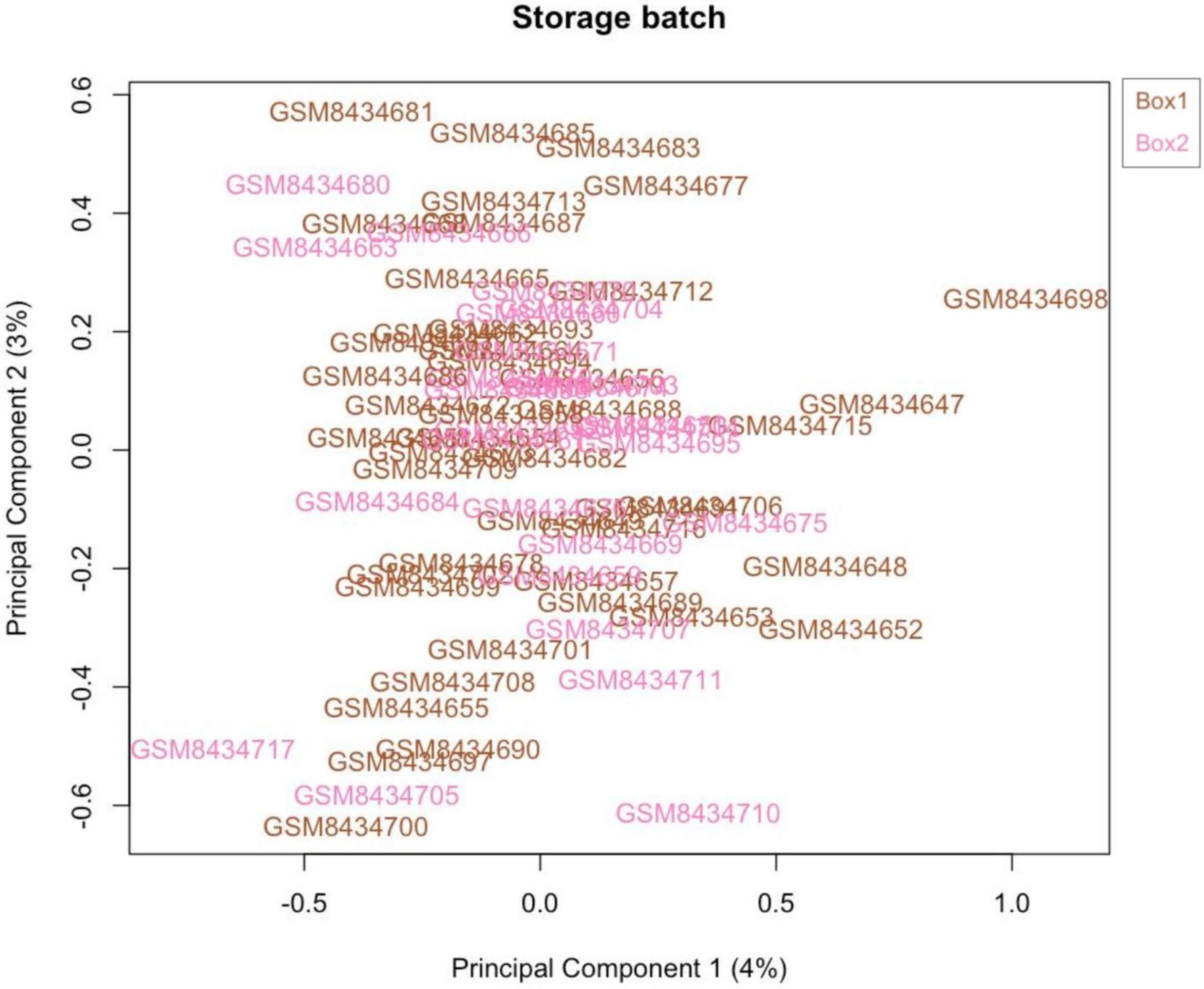

Figure S1. Results of multidimensional scaling analysis. In the analysis normalized, filtered and transformed to M-values data was used. Two principal components were visualized including 1000 top genes whose variances contributed the most to the selected components. Label colors indicate DNA storage batches.

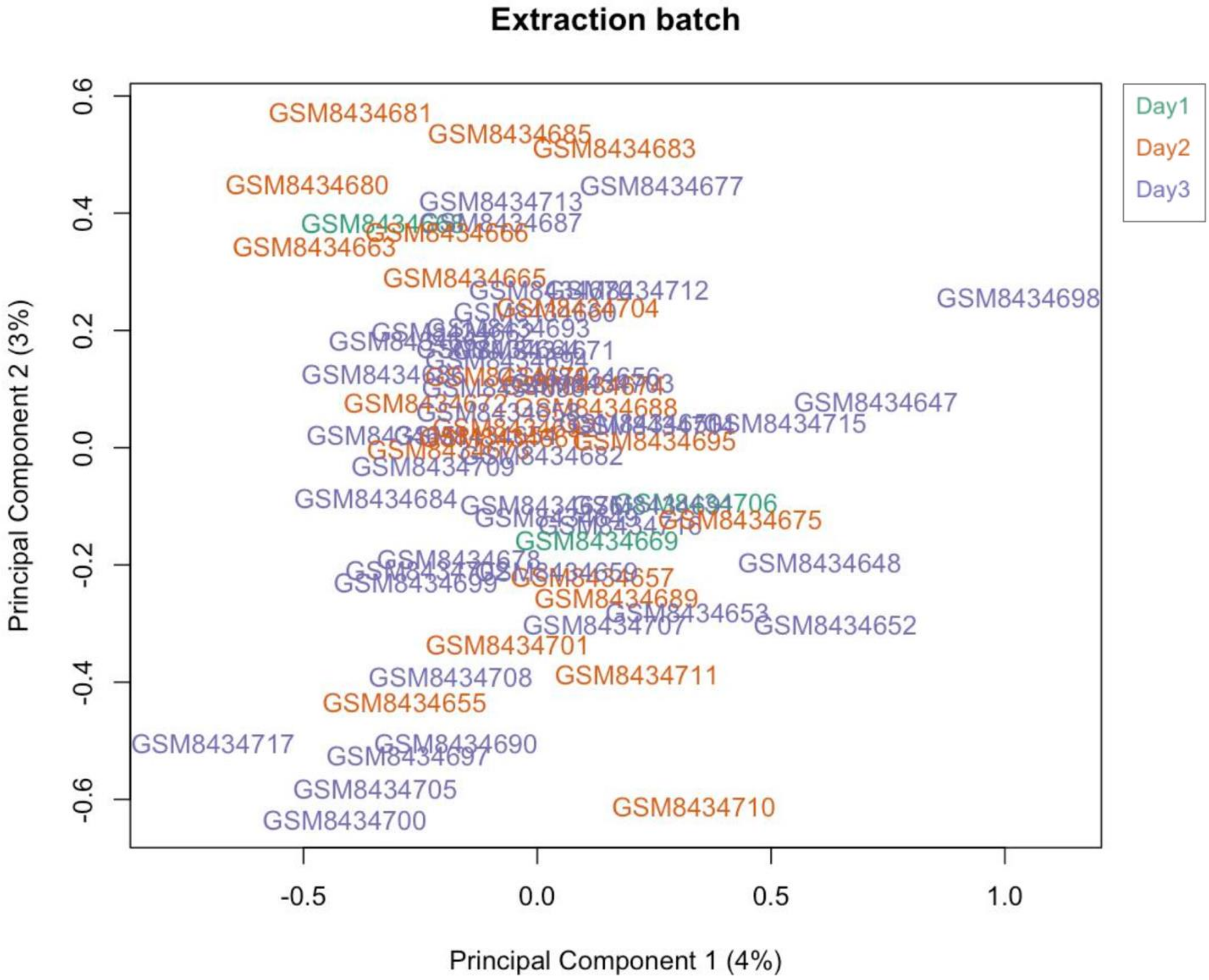

Figure S2. Results of multidimensional scaling analysis. In the analysis normalized, filtered and transformed to M-values data was used. Two principal components were visualized including 1000 top genes whose variances contributed the most to the selected components. Label colors indicate DNA extraction batches.

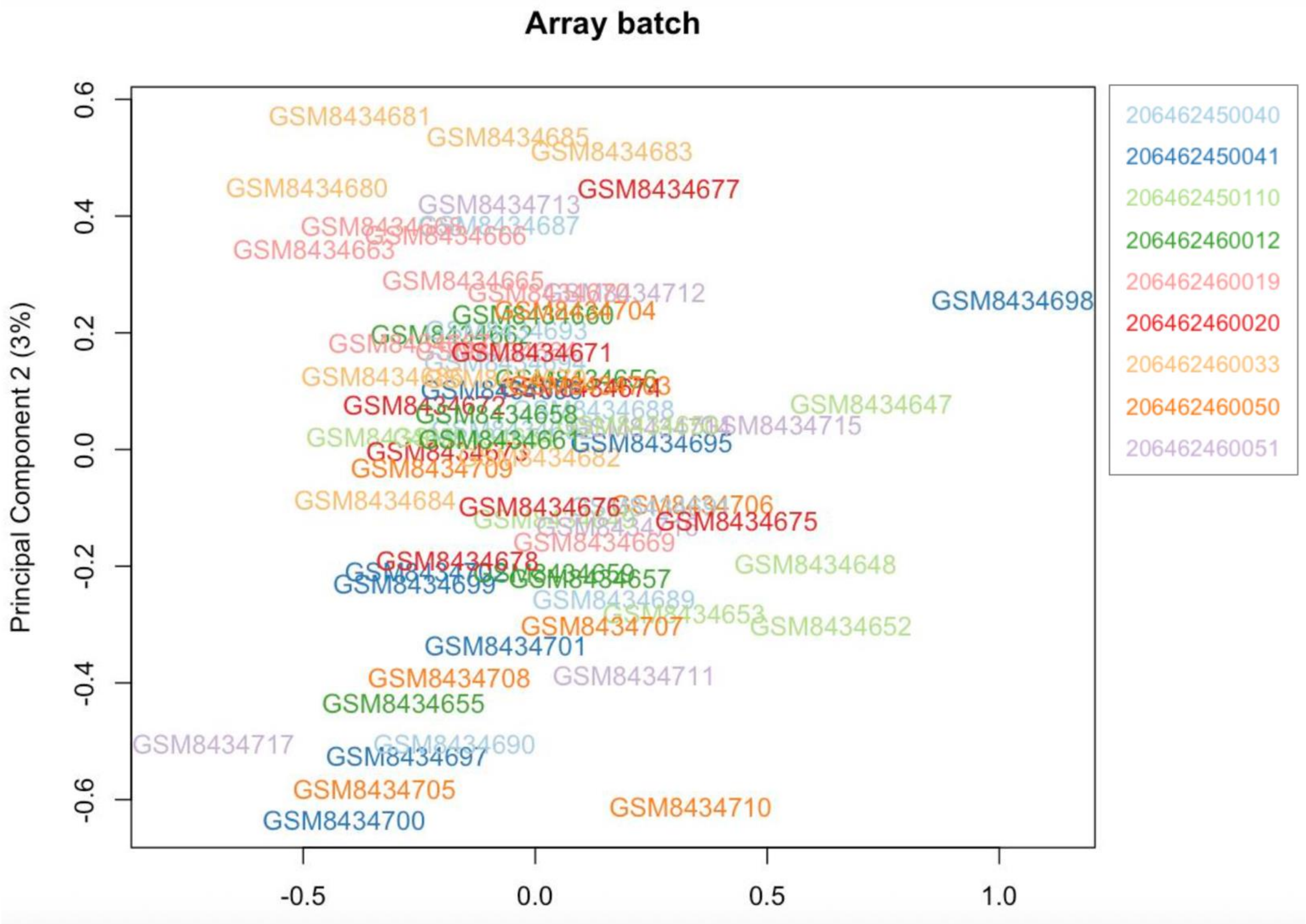

Figure S3. Results of multidimensional scaling analysis. In the analysis normalized, filtered and transformed to M-values data was used. Two principal components were visualized including 1000 top genes whose variances contributed the most to the selected components. Label colors indicate methylation array batches.

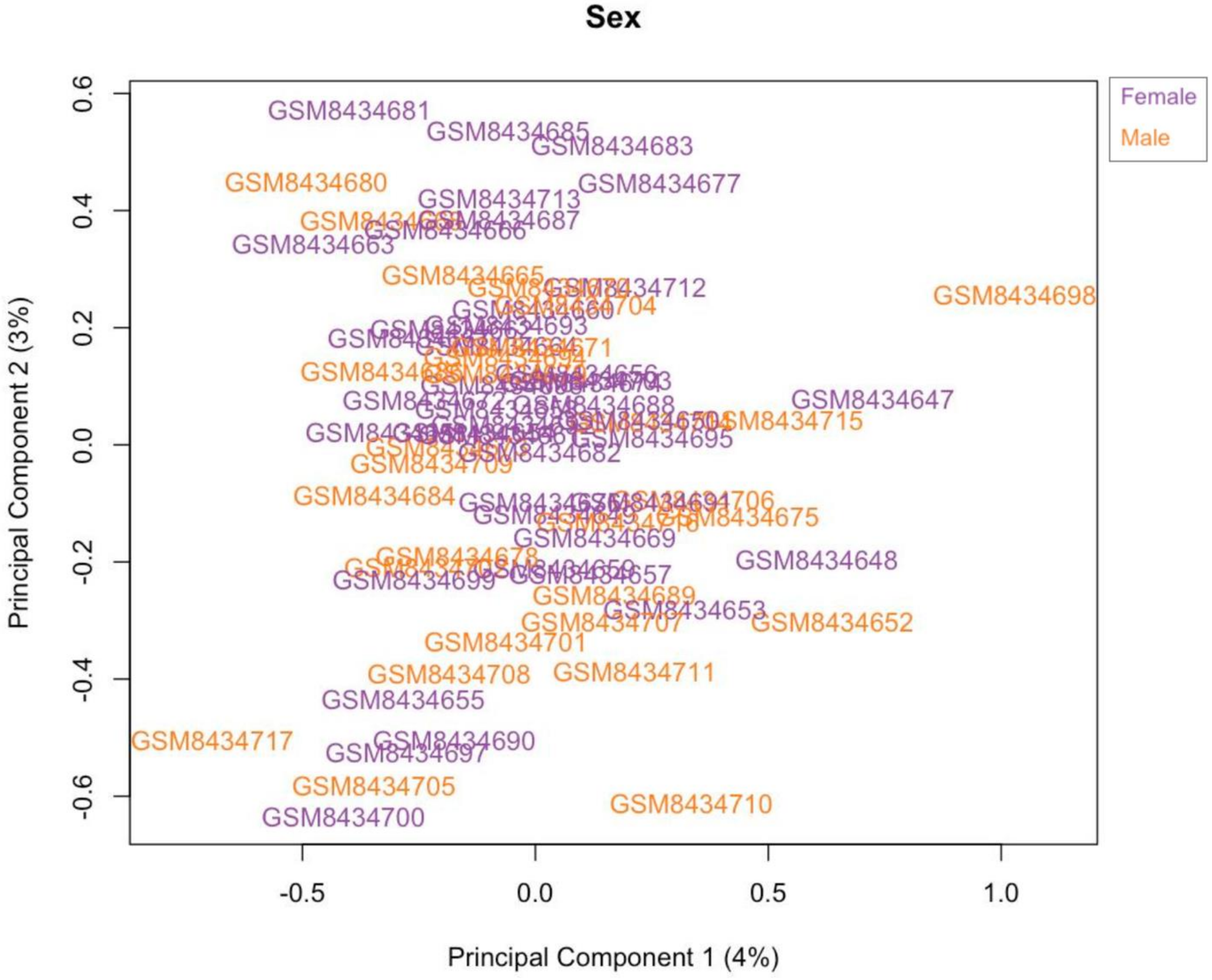

Figure S4. Results of multidimensional scaling analysis. In the analysis normalized, filtered and transformed to M-values data was used. Two principal components were visualized including 1000 top genes whose variances contributed the most to the selected components. Label colors correspond to sample sex.

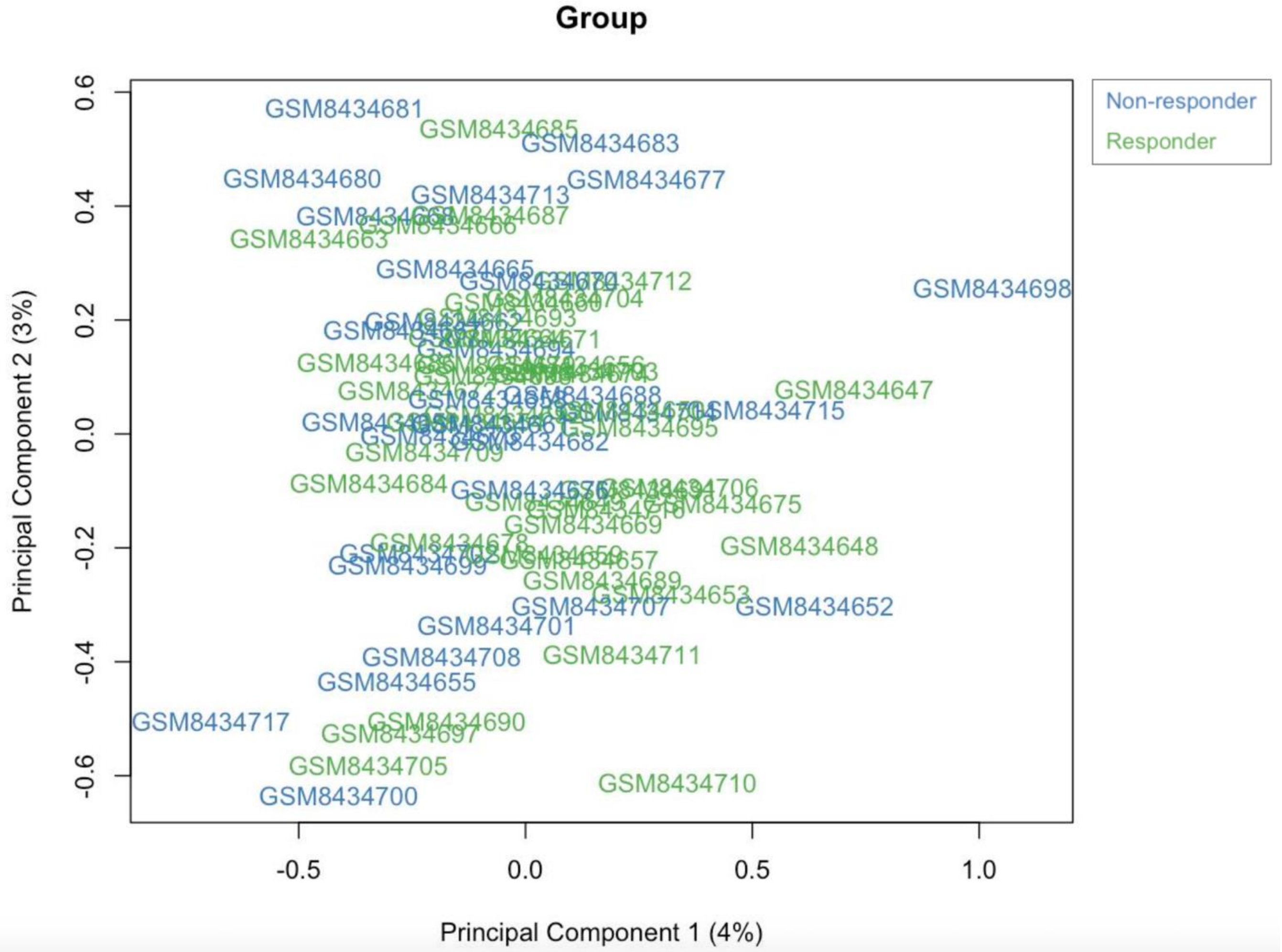

Figure S5. Results of multidimensional scaling analysis. In the analysis normalized, filtered and transformed to M-values data was used. Two principal components were visualized including 1000 top genes whose variances contributed the most to the selected components. Label colors correspond to sample phenotype group.

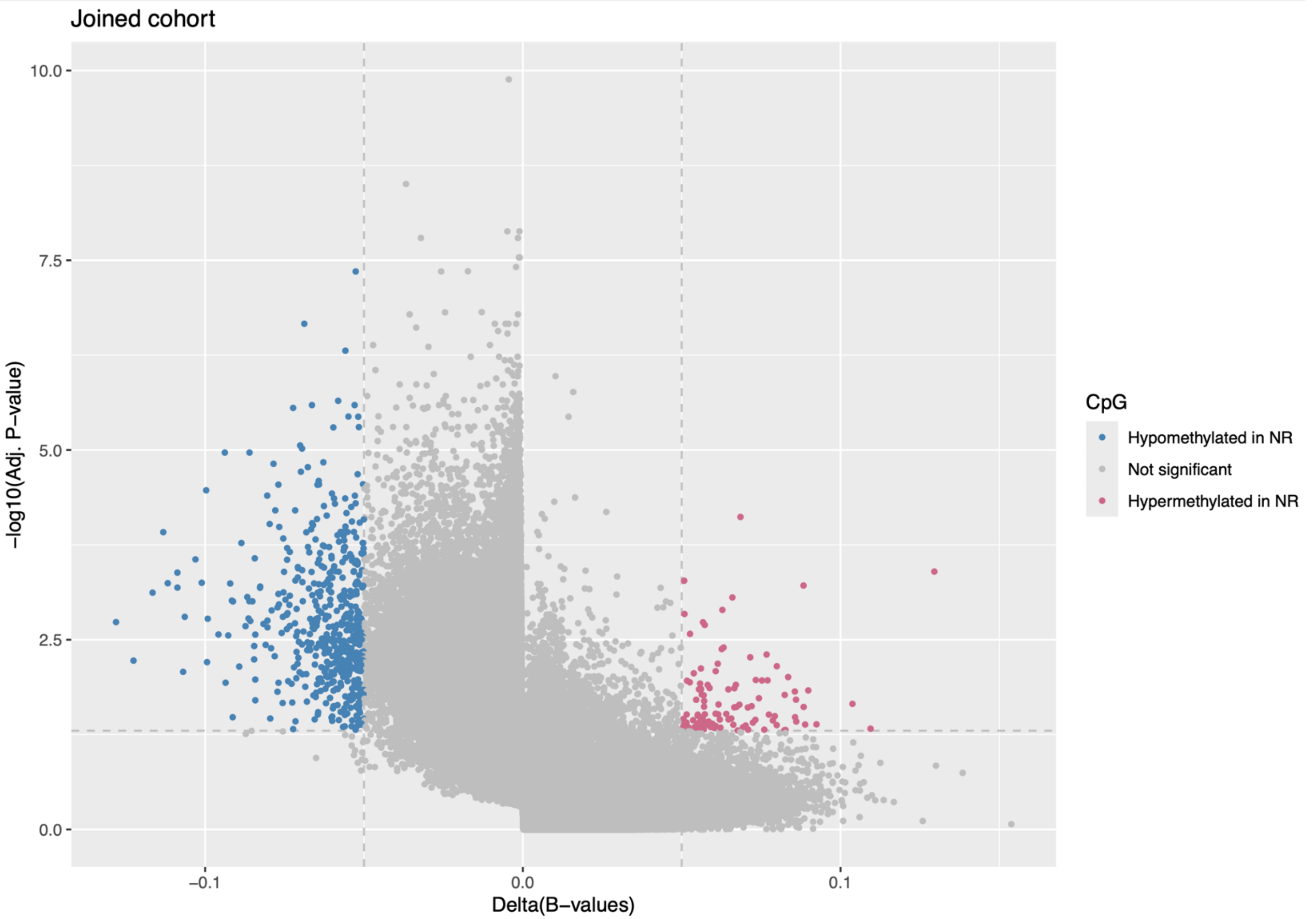

Figure S6. Volcano plot representing BH-adjusted p-values and methylation difference Delta(B-values) in all CpG sites compared between NR and R groups in aggregated cohort of females and males. Colored dots represent differentially methylated CpG sites (with methylation difference > 5% and BH-adjusted p-value < 0.05).

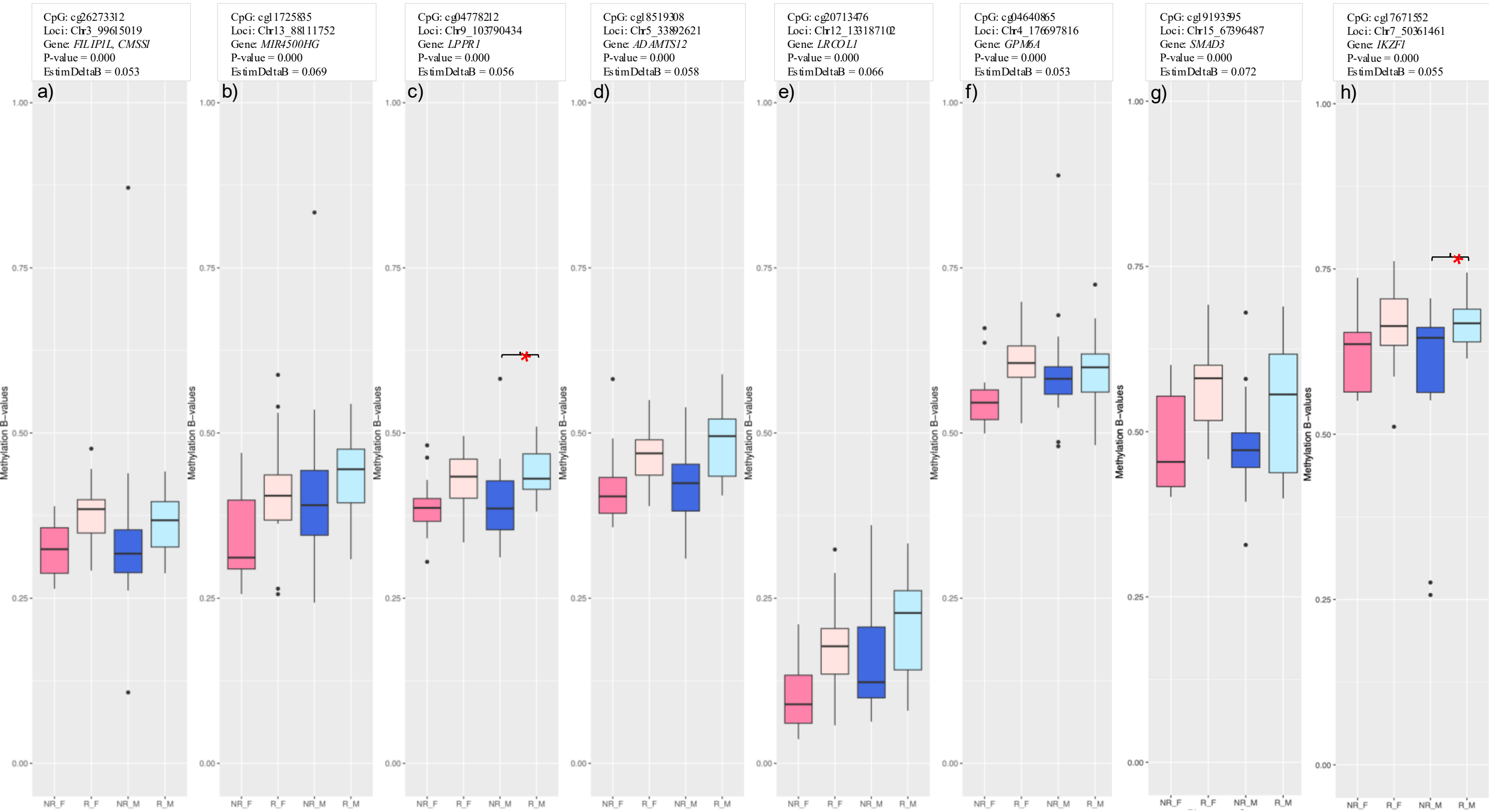

Figure S7. Methylation box plots of top genic CpG sites from sex-joined analysis. Figure visualizes a) cg26273312, b) cg11725835, c) cg04778212, d) cg18519308, e) cg20713476, f) cg04640865, g) cg19193595, h) cg17671552. Plots are ordered from left to right by increasing adjusted p-value. Asterisks indicate significant differences between R and NR groups.

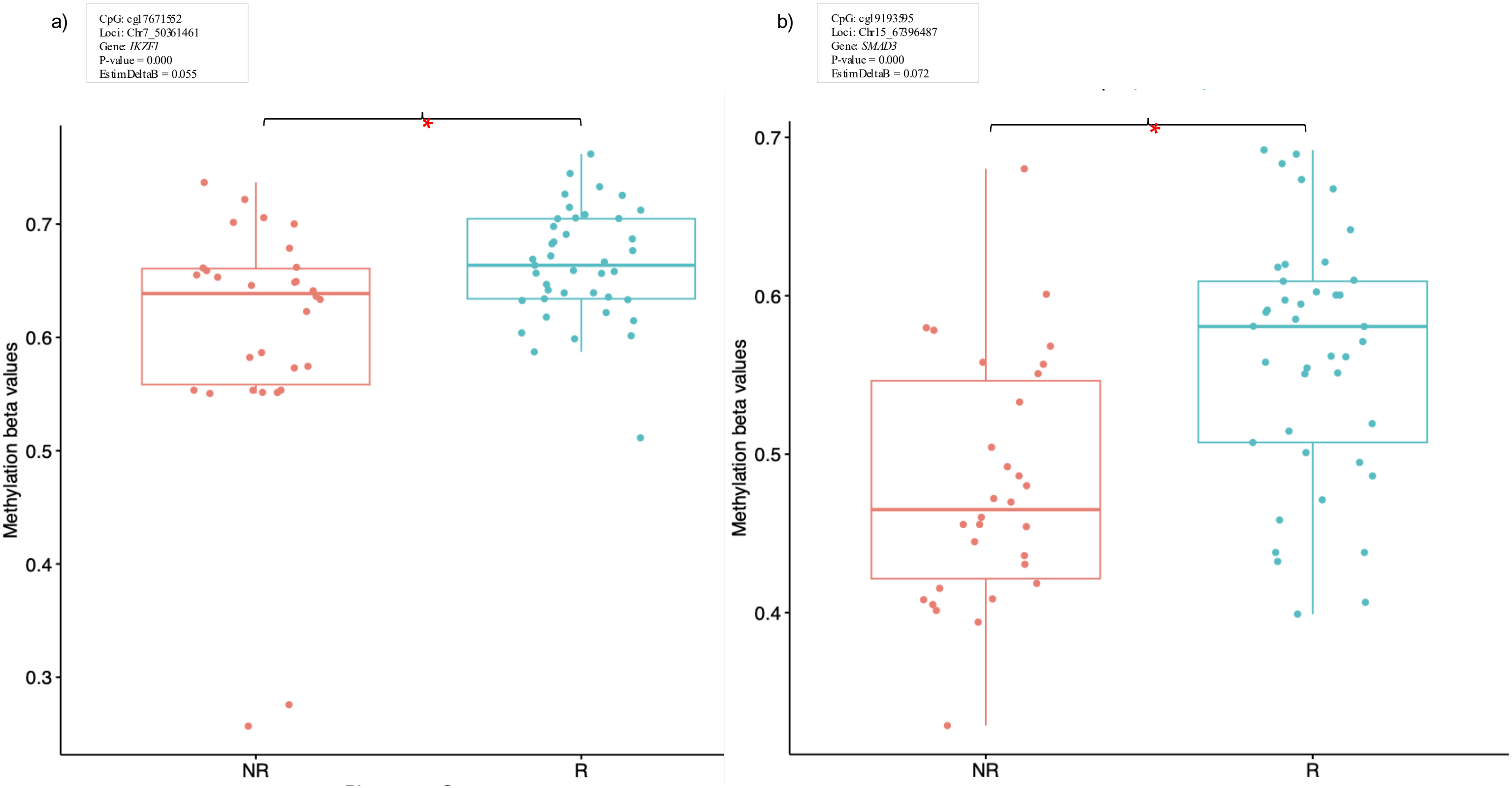

Figure S8. Methylation box plot. Figure visualizes a) cg17671552 CpG site within *IKZF1* gene and b) cg19193595 CpG site within *SMAD3* gene in studied cohort. Asterisks indicate significant differences between R and NR groups.

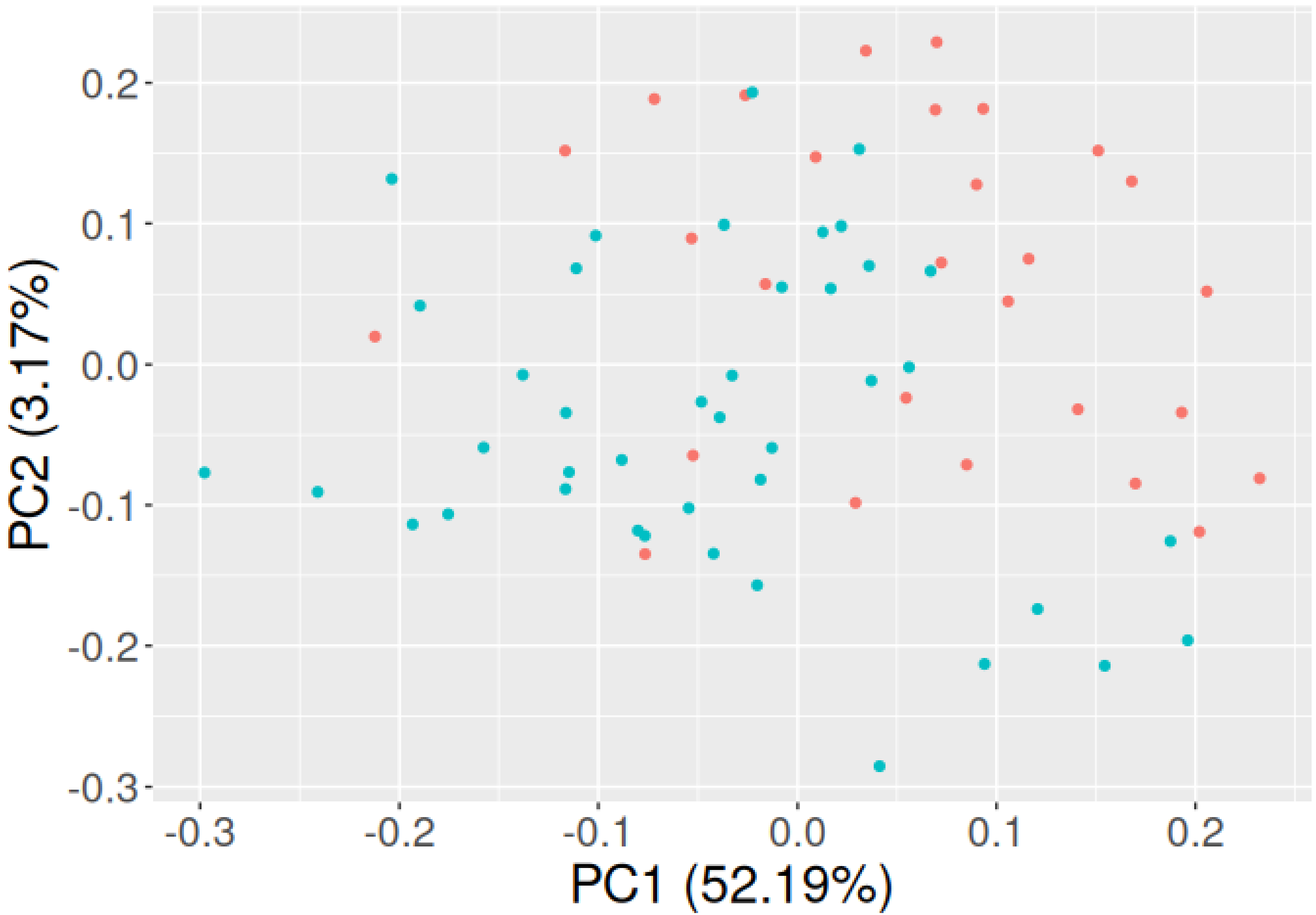

Figure S9. Summary of principal component analysis results. In the analysis we used normalized β-values of 631 DMPs emerged as significantly differentially methylated between R (turquoise dots) and NR (red dots) in sex-joined cohort.

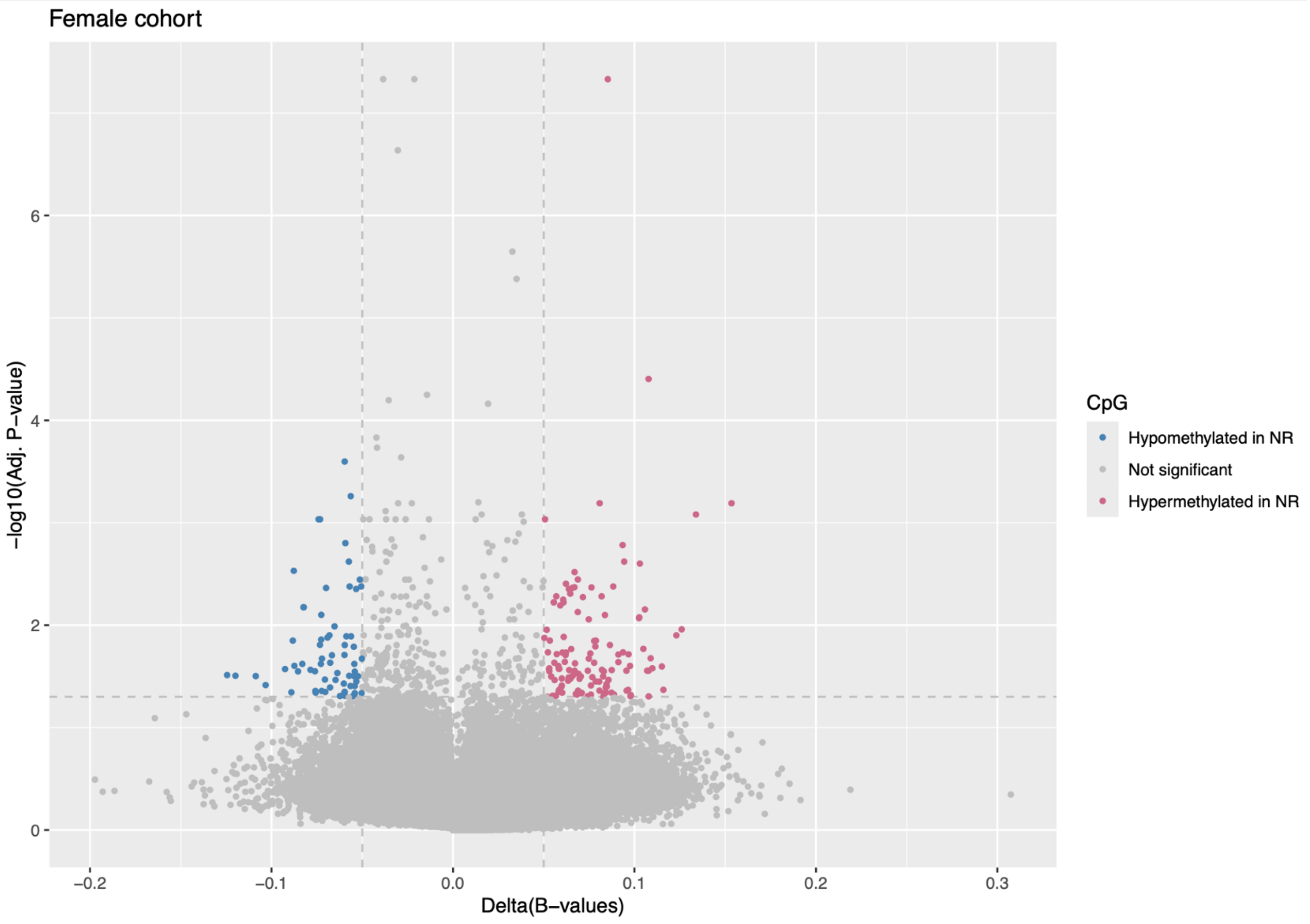

Figure S10. Volcano plot representing BH-adjusted p-values and methylation difference Delta(B-values) in all CpG sites compared between NR and R groups in female cohort. Colored dots represent differentially methylated CpG sites (with methylation difference > 5% and BH-adjusted p-value < 0.05).

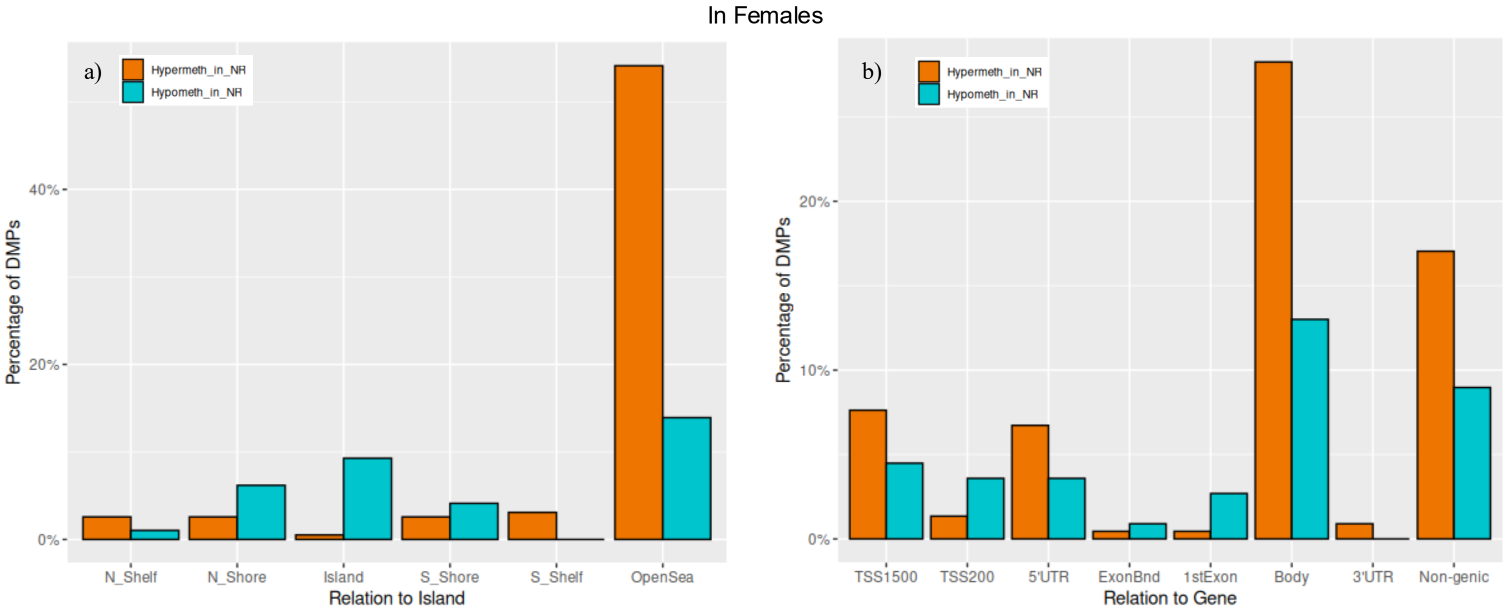

Figure S11. Distribution of DMPs emerged from DMA of female cohort (hyper and hypo-methylated). Barplots visualize distribution in relation to a) CpG islands and b) gene regions. Y-axis indicates percentage of significant CpGs belonging to a particular subregions.

Similar to the trends observed in the analysis of the entire cohort (Figure 1), the differential methylation signal tended to accumulate in the open sea of CpG islands (68% of all DMPs) and in the body of genes (56% of genic DMPs). However, unlike sex-independent DMPs, the gene body and open sea regions were mostly hypermethylated in the NR group.

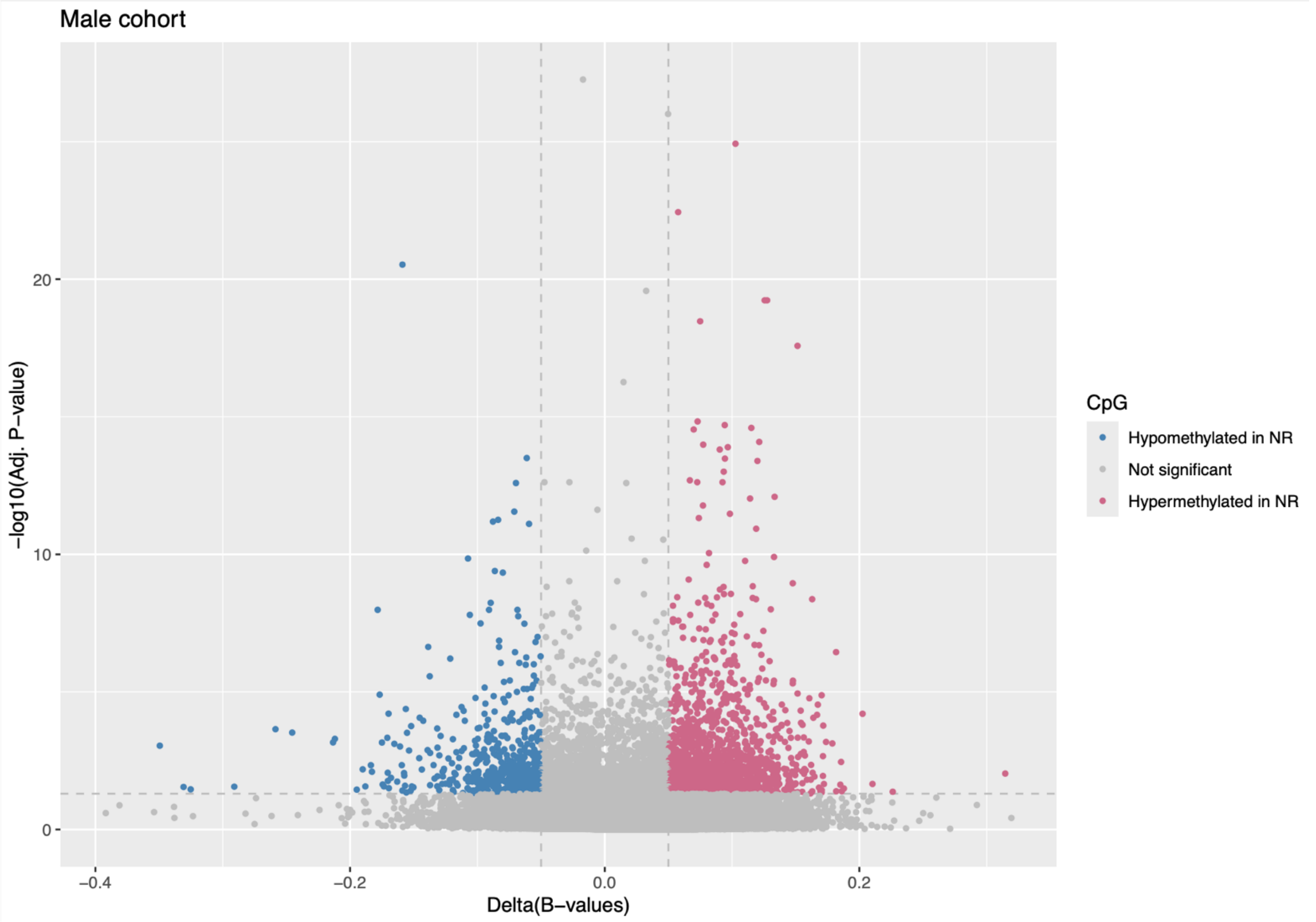

Figure S12. Volcano plot representing BH-adjusted p-values and methylation difference Delta(B-values) in all CpG sites compared between NR and R groups in female cohort. Colored dots represent differentially methylated CpG sites (with methylation difference > 5% and BH-adjusted p-value < 0.05).

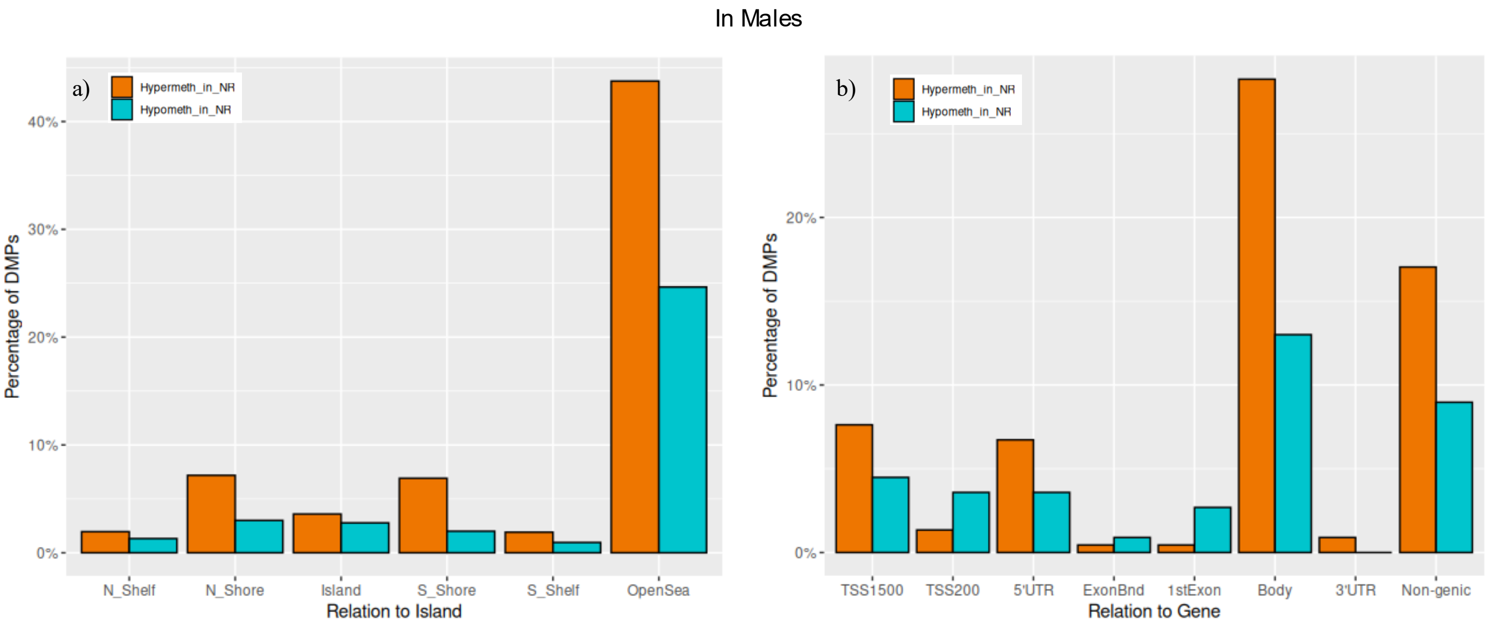

Figure S13. Distribution of DMPs emerged from DMA of male cohort (hyper and hypo-methylated). Barplots visualize distribution in relation to a) CpG islands and b) gene regions. Y-axis indicates percentage of significant CpGs belonging to a particular subregions.

Most (68%) of the total DMPs were spread along the open sea of CpG islands and 57% of genic DMPs were located in the gene body. Both overrepresented subregions were predominantly hypermethylated in NR.

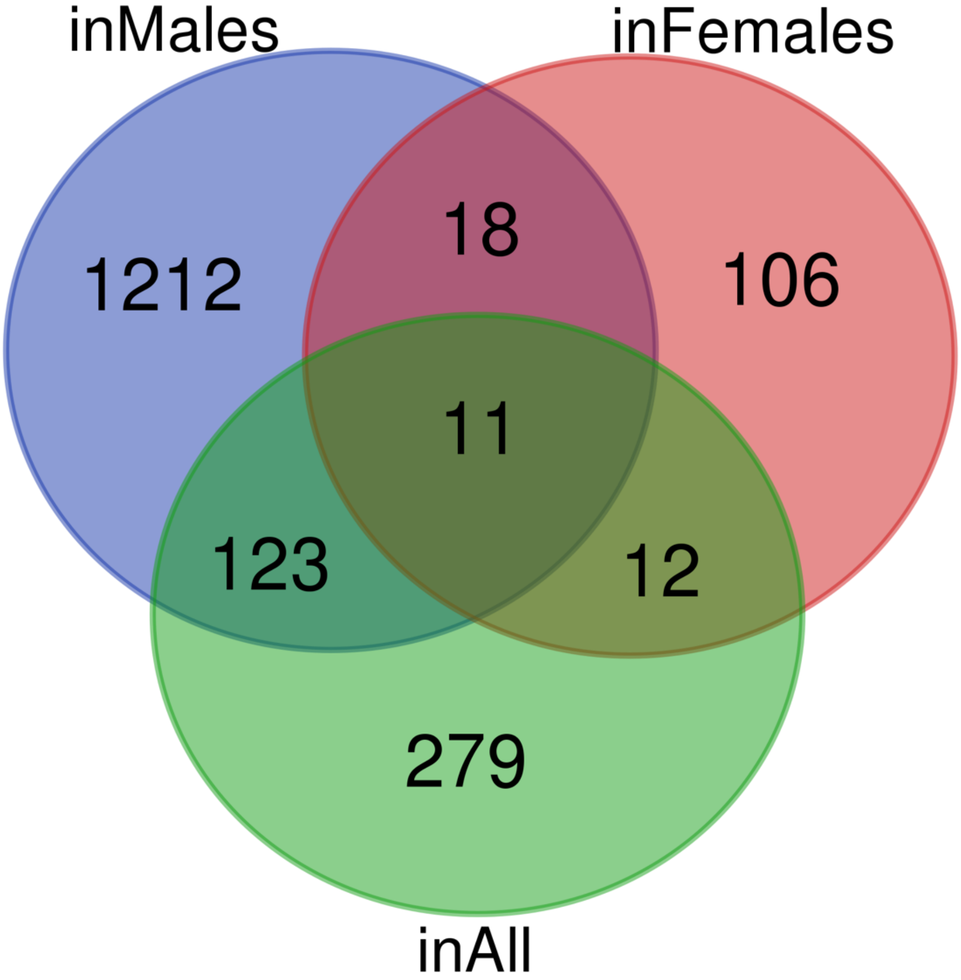

Figure S14. Overlaps between gene lists emerged from joined and sex-specific DMP analysis.

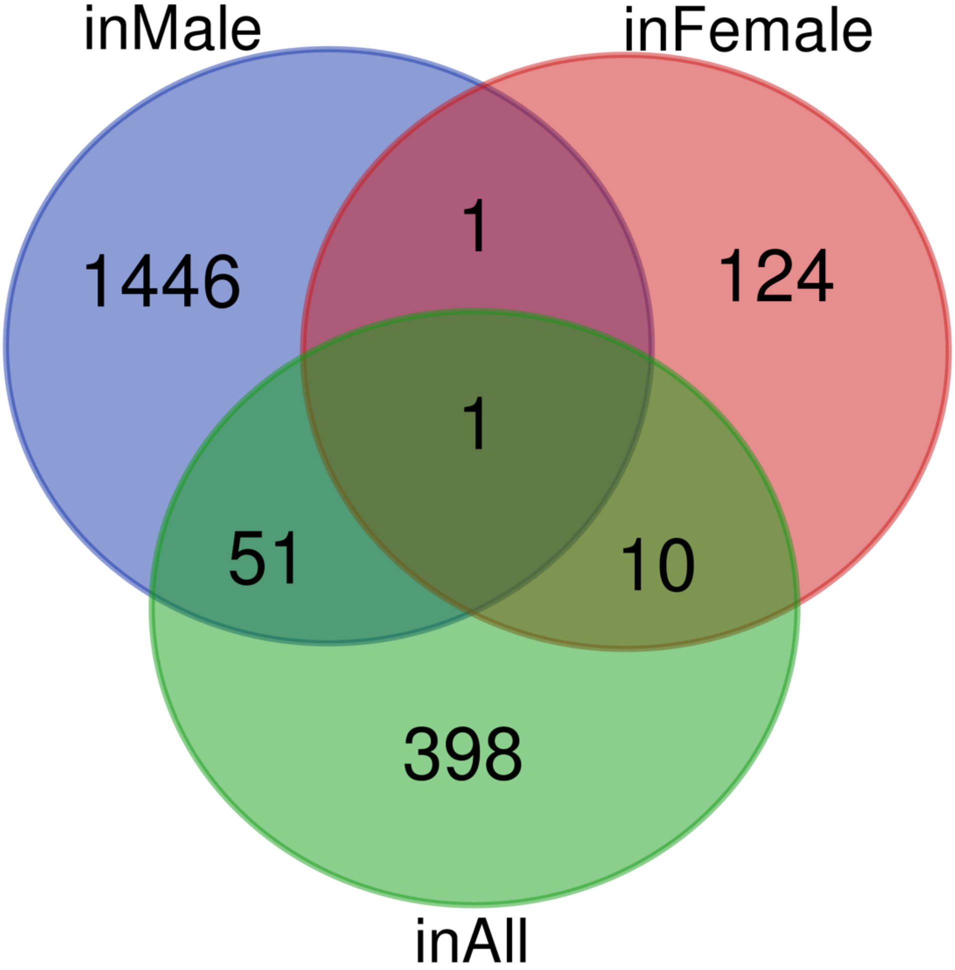

Figure S15. Overlaps between CpG sites lists emerged from joined and sex-specific DMP analysis.
