# Supplementary material for: B-cell DNA methylation signature in response to hepatitis B virus vaccination in females and males
Source: Front Immunol. 2026 Apr 10;17:1734384. doi: 10.3389/fimmu.2026.1734384 (PMC13105942; doi:10.3389/fimmu.2026.1734384)
Supplement: Supplementary file 5 [file DataSheet5.pdf]

Table W1. Results of effect size analysis.

CpGs with Abs(Delta(B-values)) > 5%	**Joined cohort**	**Female cohort**	**Male cohort**
Total number	7150	34162	48094
with Cohen's D >= 0.3 < 0.5 (small effect)	2532 (35%)	413 (1%)	2791 (6%)
with Cohen's D >= 0.5 < 0.8 (medium effect)	4069 (57%)	2907 (9%)	9162 (19%)
with Cohen's D >= 0.8 (large effect)	422 (6%)	30563 (89%)	32809 (68%)

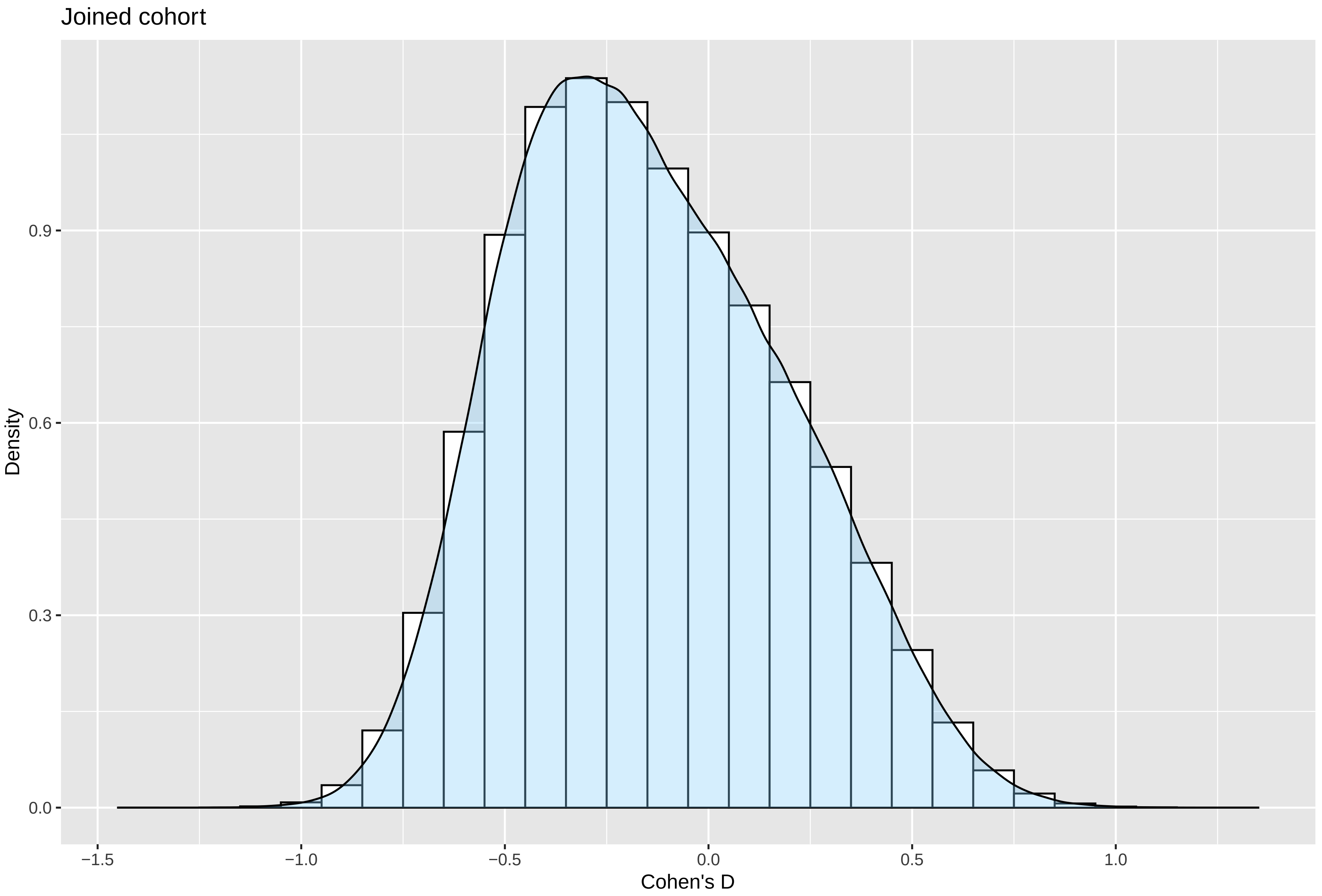

Figure W1. Distribution of Cohen's D coefficient calculated between R and NR groups.

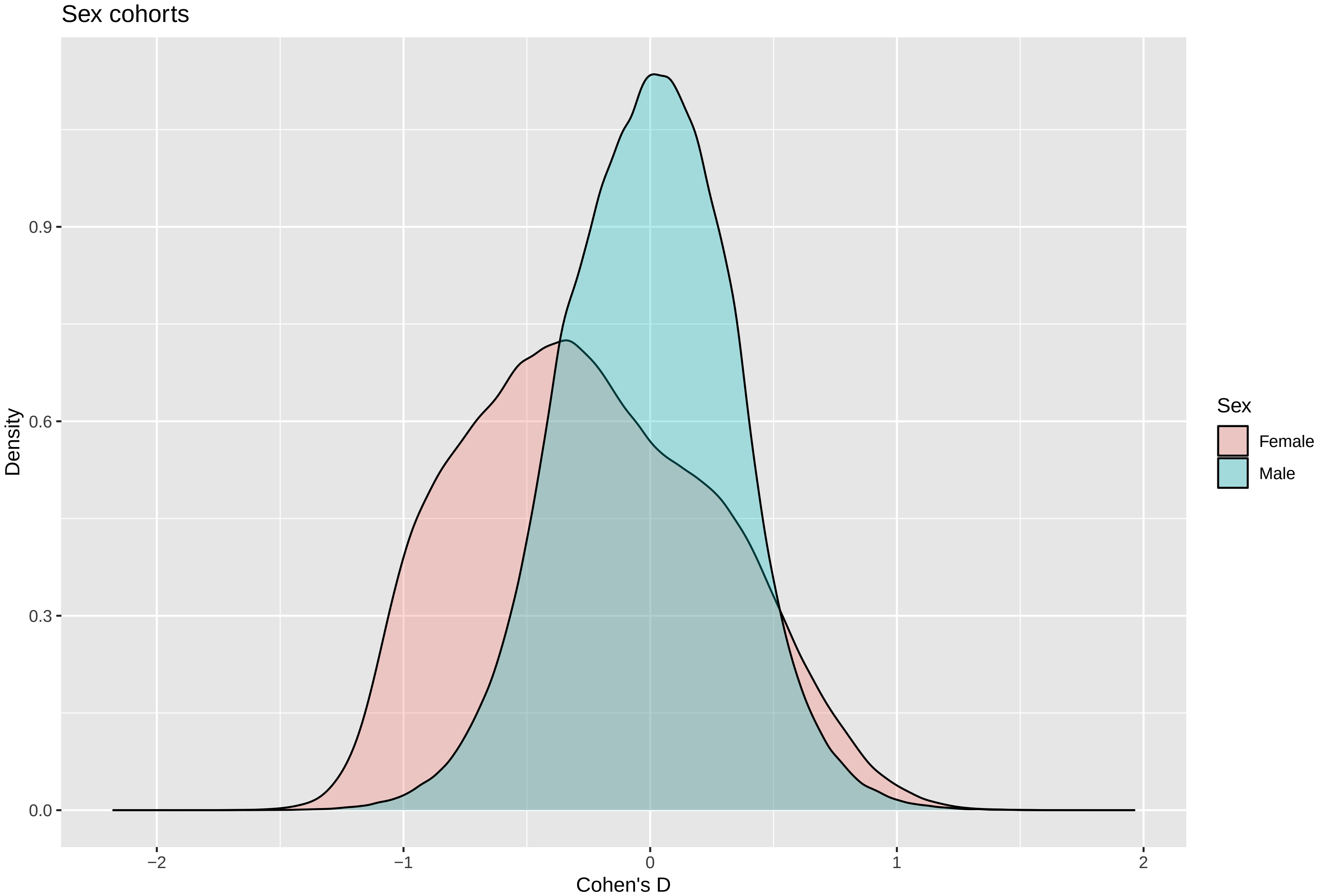

Figure W2. Distribution of Cohen's D coefficient calculated between R and NR groups.

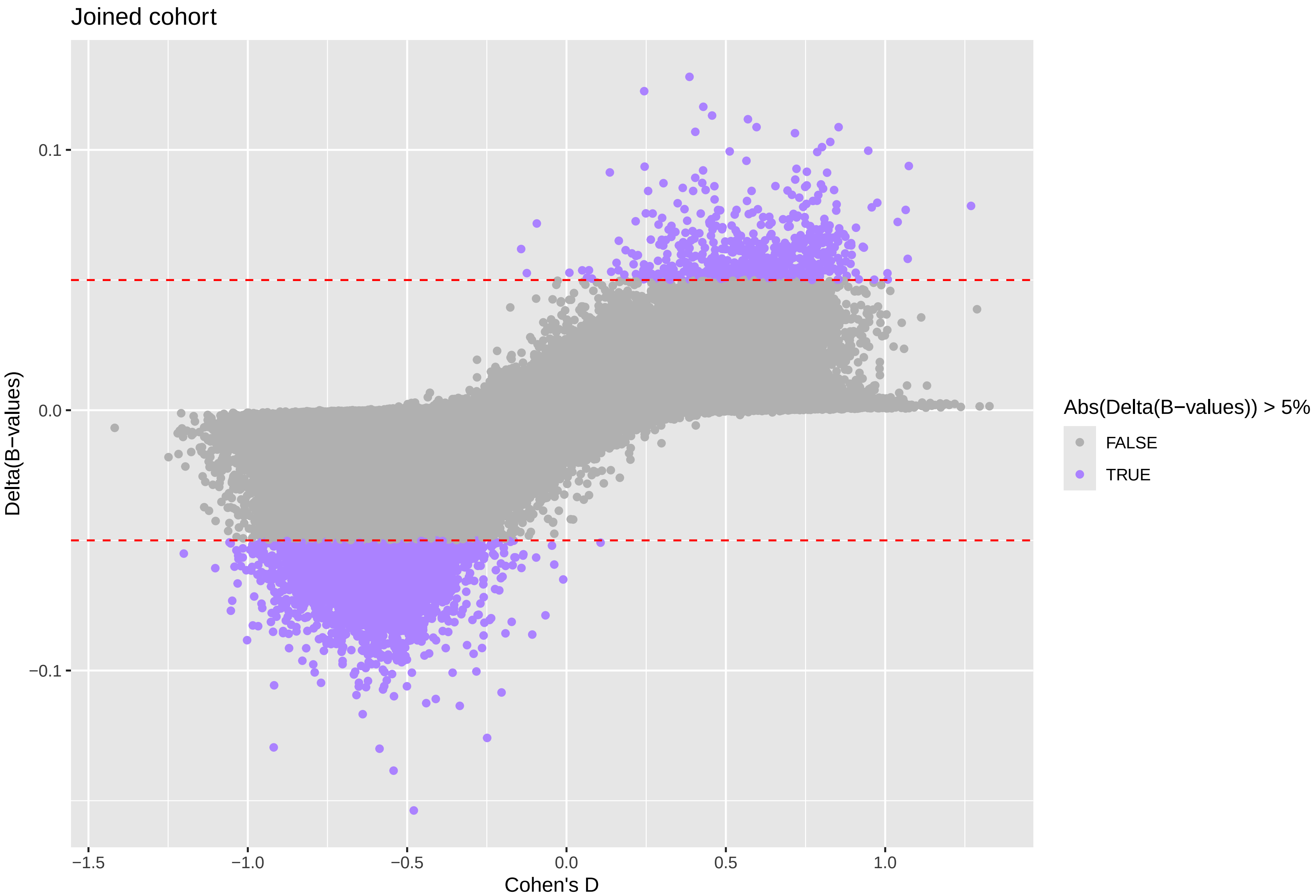

Figure W3. Difference in methylation levels versus Cohen's D coefficient calculated between R and NR groups.

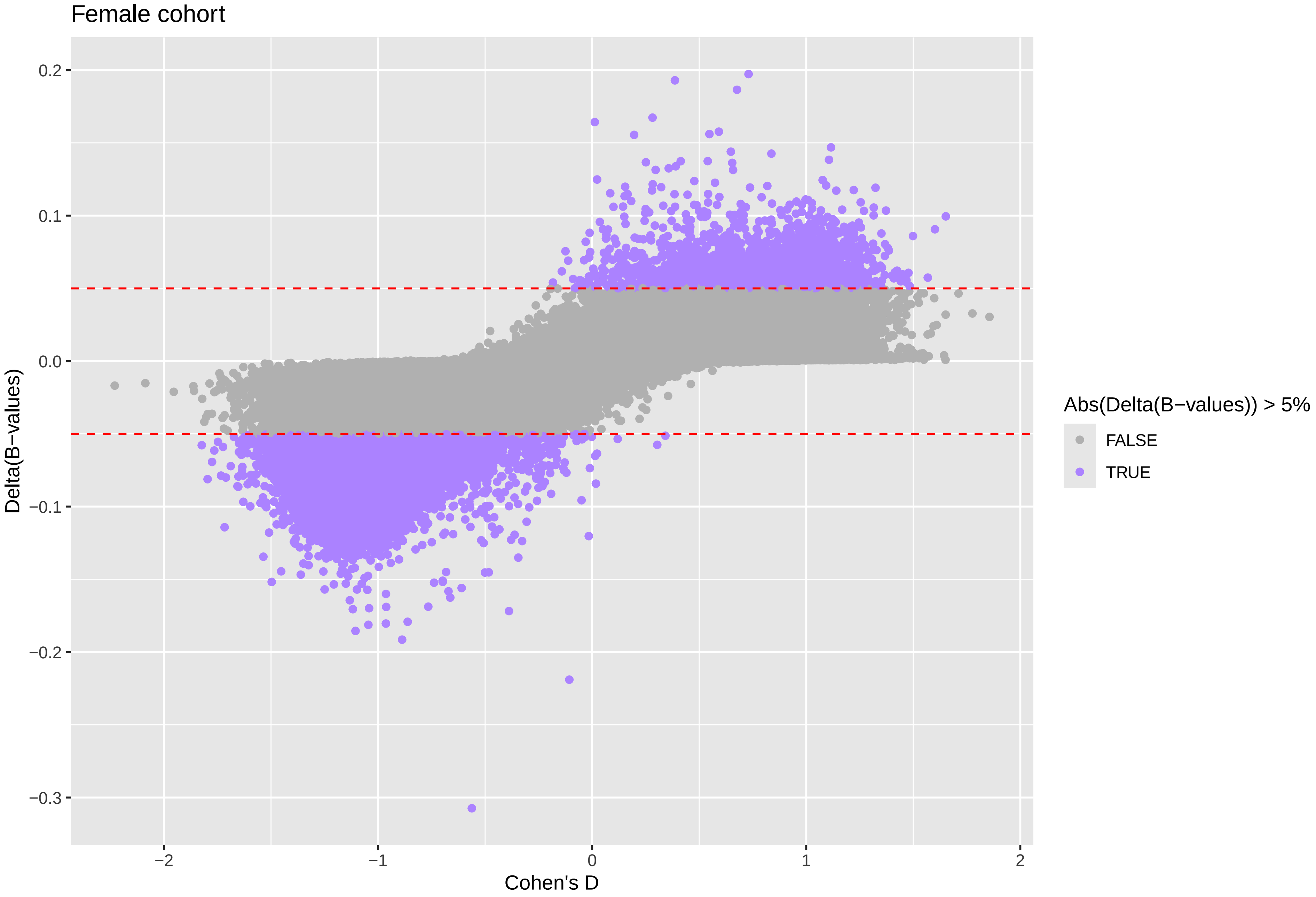

Figure W4. Difference in methylation levels versus Cohen's D coefficient calculated between R and NR groups.

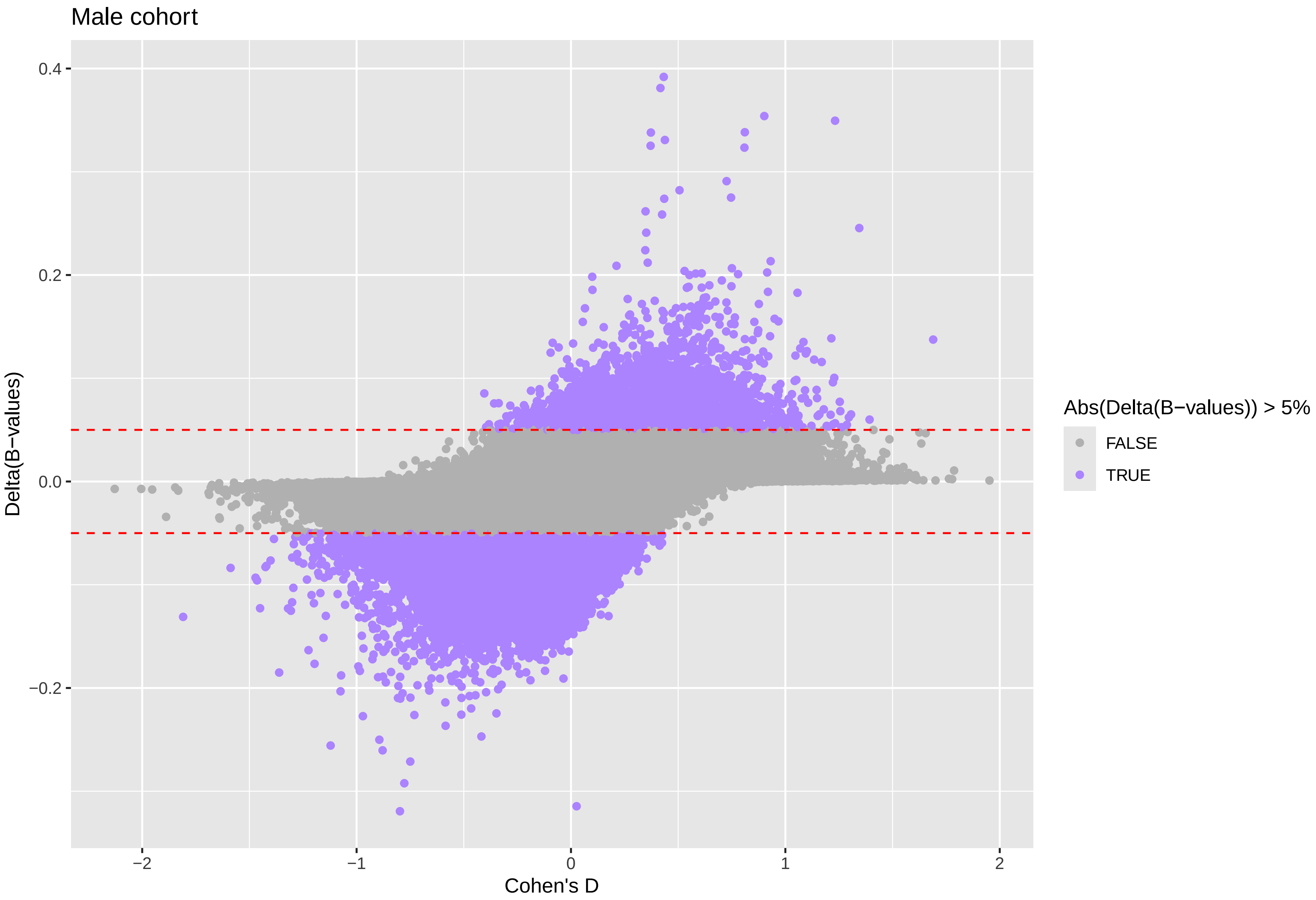

Figure W5. Difference in methylation levels versus Cohen's D coefficient calculated between R and NR groups.
